# Transcriptomics of cardiac biopsies reveals differences in patients with or without diagnostic parameters for heart failure with preserved ejection fraction

**DOI:** 10.1038/s41598-019-39445-2

**Published:** 2019-02-28

**Authors:** Sarbashis Das, Christoffer Frisk, Maria J. Eriksson, Anna Walentinsson, Matthias Corbascio, Camilla Hage, Chanchal Kumar, Michaela Asp, Joakim Lundeberg, Eva Maret, Hans Persson, Cecilia Linde, Bengt Persson

**Affiliations:** 1 Department of Cell and Molecular Biology, Science for Life Laboratory, Uppsala University, S-751 24 Uppsala, Sweden; 2 Karolinska University Hospital, Department of Clinical Physiology, S-171 76 Stockholm, Sweden; 3 Karolinska Institutet, Department of Molecular Medicine and Surgery, S-171 77 Stockholm, Sweden; 4 Translational Sciences, Cardiovascular, Renal and Metabolic Diseases, IMED Biotech Unit, AstraZeneca, S-431 83 Gothenburg, Sweden; 5 Karolinska University Hospital, Department of Thoracic Surgery, S-171 76 Stockholm, Sweden; 6 Karolinska Institutet, Department of Medicine, S-171 77 Stockholm, Sweden; 7 Karolinska University Hospital, Heart and Vascular Theme, S-171 76 Stockholm, Sweden; 8 Integrated Cardio Metabolic Center (ICMC), Department of Medicine, Karolinska Institutet, S-141 57 Huddinge, Sweden; 9 Science for Life Laboratory, Royal Institute of Technology, S-171 21 Stockholm, Sweden; 10 Karolinska Institutet, Department of Clinical Sciences, Danderyd Hospital, S-182 88 Stockholm, Sweden; 11 Danderyd Hospital, Department of Cardiology, S-182 88 Stockholm, Sweden; 12 Department of Medical Biochemistry and Biophysics, Science for Life Laboratory, Karolinska Institutet, S-17177 Stockholm, Sweden

## Abstract

Heart failure affects 2–3% of adult Western population. Prevalence of heart failure with preserved left ventricular (LV) ejection fraction (HFpEF) increases. Studies suggest HFpEF patients to have altered myocardial structure and functional changes such as incomplete relaxation and increased cardiac stiffness. We hypothesised that patients undergoing elective coronary bypass surgery (CABG) with HFpEF characteristics would show distinctive gene expression compared to patients with normal LV physiology. Myocardial biopsies for mRNA expression analysis were obtained from sixteen patients with LV ejection fraction ≥45%. Five out of 16 patients (31%) had echocardiographic characteristics and increased NTproBNP levels indicative of HFpEF and this group was used as HFpEF proxy, while 11 patients had Normal LV physiology. Utilising principal component analysis, the gene expression data clustered into two groups, corresponding to HFpEF proxy and Normal physiology, and 743 differentially expressed genes were identified. The associated top biological functions were cardiac muscle contraction, oxidative phosphorylation, cellular remodelling and matrix organisation. Our results also indicate that upstream regulatory events, including inhibition of transcription factors *STAT4*, *SRF* and *TP53*, and activation of transcription repressors *HEY2* and *KDM5A*, could provide explanatory mechanisms to observed gene expression differences and ultimately cardiac dysfunction in the HFpEF proxy group.

## Introduction

Coronary artery disease and hypertension are the most common etiologic factors for heart failure (HF). HF is characterised according to left ventricular ejection fraction (LVEF) as preserved (HFpEF, LVEF ≥ 45%) or reduced (HFrEF < 45%), as defined in our PREFERS design paper^1^. Heart failure affects 2–3% of the adult Western population^2,3^ and prevalence increases with age^4,5^. In particular, the proportion of HFpEF is increasing^6,7^, with poor prognosis^8^ comparable to HFrEF. In contrast to HFrEF, there is currently no available evidence based therapy for HFpEF^9^, which may be explained by different pathophysiology between these HF conditions. It was proposed that co-morbidities such as ischemia, hypertension and diabetes may be the drivers of disease progression in HFpEF^10^ through diverse mechanisms more specific for HFpEF than HFrEF involving coronary microvascular inflammation and endothelial dysfunction leading to intra- and extracellular rearrangements. This has been demonstrated in incomplete relaxation of myocardial strips^11^ and increased passive cardiac stiffness by titin changes and increased interstitial diffuse fibrosis^10,12^. Patients undergoing coronary bypass grafting (CABG) commonly have disturbances in LV function and thus may serve as models to study HF particularly since myocardial biopsies obtained during surgery provide a unique opportunity to study the gene expression in the different types of heart failure.

At the molecular level, gene expression programs are systematically regulated by transcription factors, chromatin regulators and other factors that are important for the establishment and maintenance of the cell state. Dysregulation of these programs can result in different diseases^13^. Studies of gene expression in heart tissue has a great potential to uncover the underlying molecular mechanisms leading to HF. In this regard, previous few studies have primarily attempted to identify gene expression differences between failing hearts (HFrEF) and normal hearts using RNA-seq analyses with limited number of samples^14–16^. There are also reports on RNA-seq expression in a mouse model for cardiac hypertrophy^17^.

In this study, we hypothesised that patients with HFpEF characteristics would show distinctive gene expression compared to patients with Normal physiology. Myocardial biopsies were obtained from patients undergoing elective CABG who all had LVEF ≥ 45%^1^. We performed RNA-seq based transcriptomics analysis in order to identify genes that are dysregulated in HFpEF compared to Normal.

## Results

### Patients

From a total of 16 patients, 5 were prospectively classified as HFpEF proxy and 11 as Normal physiology. The clinical patients’ characteristics are summarised in Table 1. The majority were male with a median age of 75 years in the HFpEF proxy group and 65 years in the Normal physiology group (p-value = 0.089). Three patients had a clinical diagnosis of heart failure, all in the HFpEF proxy group (patients 9, 11 and 15, Table 2). A history of hypertension (100% and 82%) and diabetes (60% and 45%) was present in HFpEF proxy and Normal physiology groups, respectively. One patient with Normal physiology had a history of myocardial infarction. Echocardiographic variables revealing HFpEF proxy or Normal physiology are shown in Table 2.

**Table 1:** Patients' characteristics. Data are expressed as median and quartiles (Q1;Q3) or number (%).

	All patients (n = 16)	HFpEF pathophysiology (n = 5)	Normal (n = 11)	p-value
Age (years) (median (Q1;Q3))	68 (63;73)	75 (72;77)	65 (61;68)	0.089
Sex Men/Women (n (%))	15/1 (94%/6%)	4/1 (80%/20%)	11/0 (100%/0%)	0.312
Smoking Current (n (%))	1 (7%)	0	1 (10%)	0.592
Smoking Previous (n (%))	10 (67%)	3 (60%)	7 (70%)	
**Previous**				
Heart failure	3 (19%)	3 (60%)	0	0.018
Atrial fibrillation	4 (25%)	2 (40%)	2 (18%)	0.547
Myocardial infarction	1 (7%)	0	1 (10%)	1.000
Percutaneous coronary intervention	3 (19%)	2 (40%)	1 (10%)	0.524
CABG	0 (0%)	0	0	1.000
Stroke/TIA	1 (7%)	0	1 (10%)	1.000
Peripheral artery disease	2 (13%)	2 (40%)	0	0.083
Hypertension	14 (88%)	5 (100%)	9 (82%)	1.000
Diabetes type 1 type 2	8 (50%) 1 (7%) 7 (44%)	3 (60%) 0 3 (60%)	5 (45%) 1 (10%) 4 (36%)	1.000
COPD	0 (0%)	0	0	
Anemia	0 (0%)	0	0	
Cancer	1 (7%)	0	1 (10%)	1.000
**At enrolment** (median (Q1;Q3))				
BMI	27 (24;30)	27 (26;28)	28 (23;32)	0.739
Systolic blood pressure (mmHg)	135 (130;145)	148 (138;156)	130 (125;140)	0.112
Diastolic blood pressure (mmHg)	80 (75;80)	83 (73;86)	80 (75;80)	0.537
Heart rate (beats/minute)	66 (57;77)	66 (51;81)	66 (63;72)	0.847
Creatinine (μmol/L)	82 (75;102)	84 (76;104)	79 (74;100)	0.867
Hb (g/L)	144 (137;153)	151 (136;159)	144 (137;149)	0.609
Sodium (mmol/L)	140 (138;141)	140 (140;141)	140 (136;141)	0.733
Potassium (mmol/L)	4.0 (3.8;4.3)	3.9 (3.3;4.3)	4.0 (3.8;4.3)	0.592
Troponin T (ng/L)	10 (7;15)	16 (10;17)	9 (5;11)	0.052
NT-proBNP (pmol/L)	205 (127;367)	298 (190;697)	181 (84;338)	0.090
LDL (mmol/L)	2.0 (1.5;2.8)	1.5 (1.4;2.0)	2.3 (1.5;3.0)	0.408
Triglycerides (mmol/L)	1.3 (1.0;2.0)	1.2 (1.0;1.3)	1.4 (1.1;2.0)	0.413
HbA1c (mmol/mol)	41 (37;46)	41 (40;46)	43 (35;46)	0.780
Urate (μmol/L)	359 (261;423)	405 (249;496)	355 (273;419)	0.579
**Treament** (n (%))				
Nitrates (long standing)	8 (50%)	3 (60%)	5 (45%)	1.000
Antiplatelets	13 (81%)	5 (100%)	10 (91%)	1.000
Anticoagulants	2 (13%)	2 (40%)	0	0.083
Betablockers	15 (94%)	4 (80%)	11 (100%)	0.313
ACE inhibitors	8 (50%)	3 (60%)	5 (45%)	1.000
ARBs	10 (63%)	4 (80%)	6 (55%)	0.588
Statins	16 (100%)	5 (100%)	11 (100%)	1.000
Loop diuretics	0 (0%)	0	0	
Tiazide diuretics	4 (25%)	1 (20%)	3 (27%)	1.000

ACE = Angiotensin converting enzyme; ARB = Angiotensin II receptor blocker; BMI = Body Mass Index; COPD = Chronic Obstructive Pulmonary Disease; CABG = Coronary Artery Bypass Surgery; NT-proBNP = N-Terminal pro-Brain Natriuretic Peptide; TIA = Transient Ischemic Attack.

**Table 2:** List of patients included in this study with notations of disease status (HFpEF/Normal), age, gender, comorbidities, and echocardiographic parameters.

Patient nr	Group	Age	Gender	Previous medical history						LVEF (%)	LAVI (mL/m2)	E/e′	E′sept (m/s)	E′lat (m/s)	TR_Vmax(m/s)	NTproBNP (ng/L)	LVGLS %	LVMI (g/m′)	RWT	LVDed (mm)	IVSTed (mm)	PWTed (mm)
				Hyper-tension	Diabetes type I	Diabetes type II	Atrial fibri-llation*	Stroke/TIA	Peri-pheral vascular disease													
1	HFpEF	81	M	x			x			68	43.5	10.6	0.060	0.070	2.4	697	−21.1	102	0.38	48	11	9
2	HFpEF	58	F	x		x				63	52.1	13.1	0.062	0.075	na	161	−20.4	94	0.45	44	13	10
9	HFpEF	71	M	x						54	40.3	8.8	0.070	0.080	2.5	190	−12.9	107	0.42	52	11	11
11	HFpEF	77	M	x		x	x		x	49	44.1	11.4	0.064	0.098	2.9	2160	−15.2	89	0.49	45	11	11
15	HFpEF	75	M	x		x			x	48	37.1	12.0	0.048	0.060	2.8	298	−15.8	121	0.32	56	13	9
3	Normal	67	M	x				x		57	31.5	5.5	0.050	0.070	na	338	−16.6	83	0.44	45	11	10
4	Normal	68	M	x						60	25.0	5.4	0.070	0.140	na	na	−16.8	101	0.39	46	14	9
5	Normal	73	M				x			57	33.1	5.8	0.065	0.120	na	396	−18.4	76	0.33	48	9	8
6	Normal	49	M	x		x				48	32.0	7.6	0.098	0.100	2.0	181	−11.9	84	0.32	50	12	8
7	Normal	64	M	x						56	27.9	9.1	0.082	0.104	na	147	−17.6	80	0.42	43	14	9
8	Normal	65	M							60	27.3	7.8	0.080	0.100	na	262	−17.0	58	0.33	43	10	7
10	Normal	60	M	x	x					66	33.5	8.0	0.072	0.124	na	219	−19.5	77	0.36	45	10	8
12	Normal	67	M	x		x	x			53	25.5	8.7	0.056	0.047	na	77	−18.5	103	0.36	50	12	9
13	Normal	61	M	x		x				65	30.0	6.8	0.092	0.116	na	107	−20.0	100	0.38	47	12	9
14	Normal	63	M	x		x				59	40.6	6.8	0.097	0.099	na	66	−19.9	84	0.37	49	11	9
16	Normal	68	M	x						61	35.8	7.6	0.094	0.113	na	84	−22.0	117	0.44	50	11	11

LVEF – left ventricular ejection fraction, LAVI – left atrial volume index, E – early mitral inflow velocity, e – early diastolic tissue velocity, sept – septal, lat – lateral, TR_Vmax - tricuspid regurgitation maximal velocity, LVGLS –left ventricular global longitudinal strain, LVMI - left ventricular mass index, RWT – relative wall thickness, LVDed – left ventricular diameter, enddiastolic, IVSTed – intraventricular septum thickness, enddiastolic, PWTed – posterior wall thickness, venddiastolic. *Only patient 11 had atrial fibrillation at echocardiography examination. Patients 9, 11 and 15 had a clinical diagnosis of heart failure.

### Gene expression profiles of LV tissue in HFpEF proxy and Normal physiology groups

**Figure 1:**
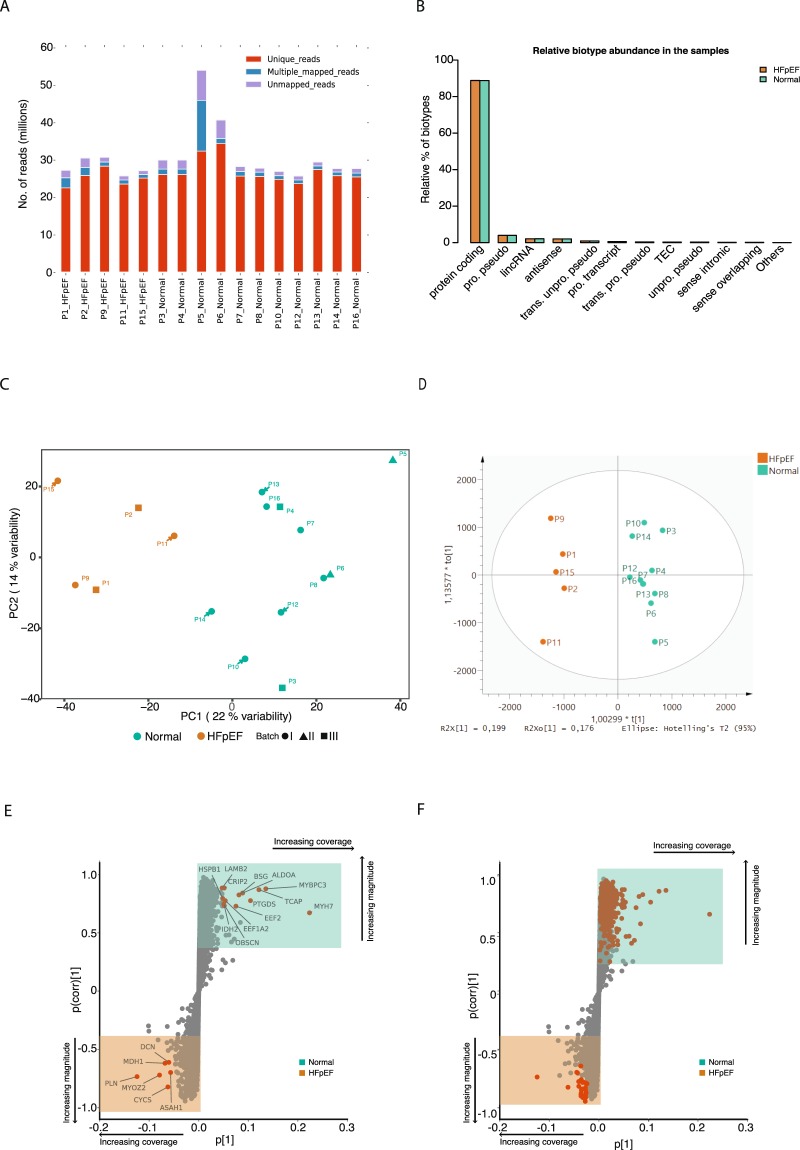
Gene expression profiles of left ventricle tissues discriminate HFpEF proxy from Normal physiology. (**A**) Stack barplot showing uniquely mapped, multiple mapped and unmapped reads. The x axis shows the samples and the y axis the number of reads. (**B**) Bar plot showing the relative abundance of each biotype (y axis) in the samples (x axis). (**C**) Principal component analysis (PCA) score plot with the two principal components (PC1 and PC2) plotted on the x- and y-axis, respectively. Each data point represents one sample, which is colour-coded according to the condition and shaped according to the sequencing batch. Green and orange colours correspond to Normal physiology and HFpEF proxy, respectively. (**D**) Orthogonal projections to latent structures discriminant analysis (OPLS-DA) score plot for the groups HFpEF proxy (orange) and Normal physiology (green). (**E**) S-plot of the OPLS-DA data showing the magnitude of each gene’s contribution to the separation, p[1], in relationship to its significance, p(corr)[1]. Genes contributing to the highest magnitude of the separation for the respective groups are highlighted in red/orange. Shaded boxes indicate up-regulated genes in the HFpEF proxy group (bottom orange box) and in the Normal physiology group (top green box). (**F**) The same plot as in “E” but genes overlapping with differentially expressed genes are highlighted with in red/orange.

The transcriptome sequencing resulted in an average of 26 million paired-end reads per biopsy sample. Number of mapped reads ranged from 17 to 30 million with an average of 24 million (Fig. 1A) which covered more than 85% of the sequenced reads. Normalisation and batch correction was performed using Trim mean of the M-values (TMM) and ARSyNseq, respectively, after filtering out lowly expressed genes (Supplementary Fig. S1). The majority (~90%) of the expressed genes were protein coding while less than 5% were detected as long noncoding RNA (lncRNA) or antisense RNA (Fig. 1B, Supplementary Fig. S2).

We first characterised differences between the gene expression profiles of the 16 samples, utilising an unsupervised classification method – Principal Component Analysis (PCA). PCA using batch corrected normalised gene expression revealed two clusters, corresponding to HFpEF proxy and Normal physiology along the first principal component 1 (PC1). The PCA model revealed that the largest variation of the PC1 could explain 22% of the variation while principal component 2 explained 14%. Additionally, any bias between these groups and sequencing batches was investigated and no batch effect contributing to this clustering was found (Fig. 1C).

The Orthogonal Projections to Latent Structures Discriminant Analysis (OPLS-DA) model with 7-fold cross-validation depicted in Fig. 1B distinguished HFpEF proxy from Normal physiology using the first predictive component. An S-plot was generated to identify signature genes in both groups (Fig. 1D). In the S-plot, the magnitude of the contribution of each gene to the OPLS-DA model (p[1]) was plotted against significance of the corresponding genes (p(corr)[1]). As can be seen in Fig. 1E, genes (marked in red) contributing most to the discrimination of HFpEF proxy were *MYH7*, *MYBPC3*, *TCAP*, *PTGDS*, *BSG*, *ALDOA*, *EEF2*, *IDH2*, *CRIP2*, *EEF1A2*, *PLN*, *CYCS*, *MYOZ2*, *MDH1* and *DCN*. Several of these genes were also found among the significantly differentially expressed genes (Fig. 1F; see below).

### Genes dysregulated in HFpEF proxy patients

**Figure 2:**
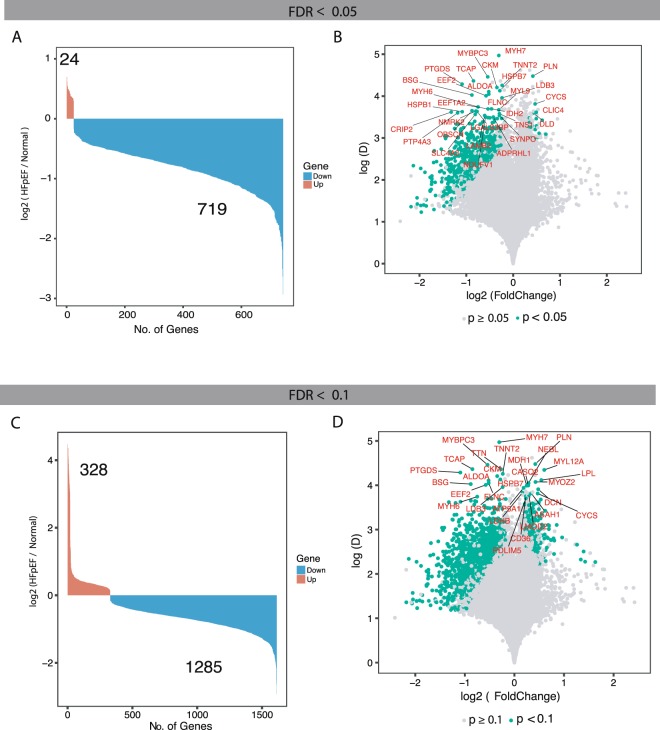
Identification and annotation of the dysregulated genes in HFpEF proxy versus Normal physiology. **(A**) Bar plot showing number of differentially expressed genes between HFpEF proxy and Normal physiology predicted using NOISeq with false discovery rate (FDR) adjusted p-value < 0.05. The x axis represents significantly dysregulated genes and the y axis showing the fold change in log2 scale of the corresponding genes. Down-regulated genes in blue and up-regulated genes in orange. (**B**) Volcano plot of the differentially expressed genes. The x axis represents fold change in log2 scale of HFpEF proxy versus Normal physiology while the y axis indicates the differences of mean expression between HFpEF proxy and Normal physiology. Each point represents a gene, and significantly expressed genes are highlighted in green. Genes that are significantly expressed (FDR-adjusted p-value < 0.05) and with a difference of mean expression above 3.5 are labelled with gene symbols. (**C**) Similar to plot “A”, but FDR-adjusted p-value < 0.1. (**D**) Similar to plot “B” but FDR-adjusted p-value < 0.1. Genes that are significantly expressed and with a difference of mean expression above 3.7 are labelled with gene symbols.

Differentially expressed genes (DEGs) were identified using NOIseqbio with a false discovery rate (FDR) < 0.05. Our analysis identified 743 DEGs discriminating between HFpEF proxy and Normal physiology whereof the majority were down-regulated in HFpEF proxy samples compared with Normal physiology samples (Fig. 2A). The distribution of fold change as a function of mean expression difference in all the cases revealed that more than 90% of significant DEGs had fold changes ranging from 1.30 to –2.60 (Fig. 2A,B). For comparison, we also calculated DEGs using FDR < 0.1 (Fig. 2C,D), resulting in 328 up-regulated and 1285 down-regulated genes in HFpEF proxy samples. The functional analysis (cf. below) of these DEGs showed similar results to those using FDR < 0.05, and we therefore kept the more stringent FDR < 0.05 DEGs in the current analysis.

Among the 743 DEGs, 69 genes were transcriptional regulators, of which 67 were down- and 2 up-regulated. A comparison between the predicted DEGs with the genes in the S-plot derived from OPLS-DA showed that most DEGs with few exceptions are located at largest distances from the axes centre. Thus, the dysregulated genes were predicted by two independent methods (Fig. 1F). The complete list of up- and down-regulated genes is given in the Supplementary Table S1.

**Figure 3:**
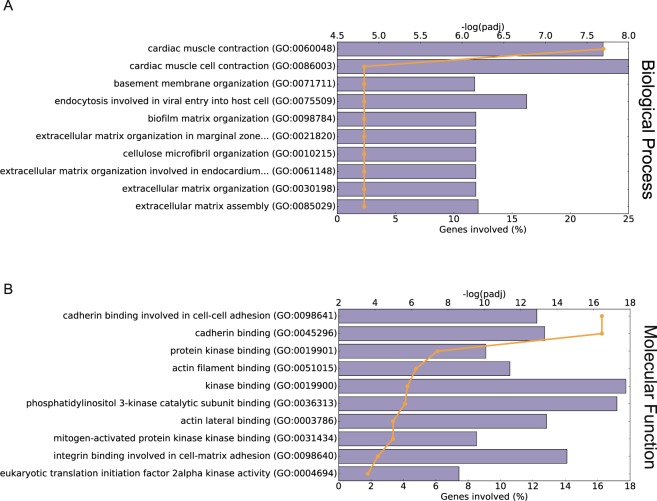
Functional classification of the differentially expressed genes. (**A**,**B**) GO annotations of biological process and molecular function, respectively, of the down-regulated genes in HFpEF proxy group. The horizontal bars show percentage of the down-regulated genes with the corresponding GO annotations (scale at bottom x axis), The orange lines represent significance of the corresponding GO annotations (scale at top x axis) as calculated by Enrichr^53^.

The top biological processes associated with down-regulated genes in the HFpEF proxy group were cardiac muscle contraction and extracellular matrix assembly/organization (Fig. 3A, Supplementary Table S2). Enriched Gene Ontology (GO) terms reflecting molecular function revealed genes for cadherin binding, kinase binding, actin filament and integrin binding (Fig. 3B, Supplementary Table S2).

We found down-regulation of genes involved in cardiac muscle contraction: myosin heavy chain 6 and 7 (*MYH6*,*MYH7*), cardiac myosin-binding protein C (*MYBPC3*), cardiac troponin T2 (*TNNT2)*, titin cap (*TCAP*) and two potassium voltage-gated channels (*KCNH2*,*KCNQ1*).

Matrix-related genes that were down-regulated in the HFpEF proxy group *LAMA5*, *LAMB2*, coding for two laminin subunits (α5 and β2), *SPARC* coding for secreted protein acidic and rich in cysteine, and a number of collagens – Iα1, IIIα1, IVα, IVβ1, VIα1, VIβ1 and XVIIIα1.

Among the up-regulated genes we identified lumican (*LUM*), phospholamban (*PLN*), myozenin 2 (*MYOZ2)*, and cytochrome c (*CYCS)*.

### Upstream regulators and regulatory effect networks

**Figure 4:**
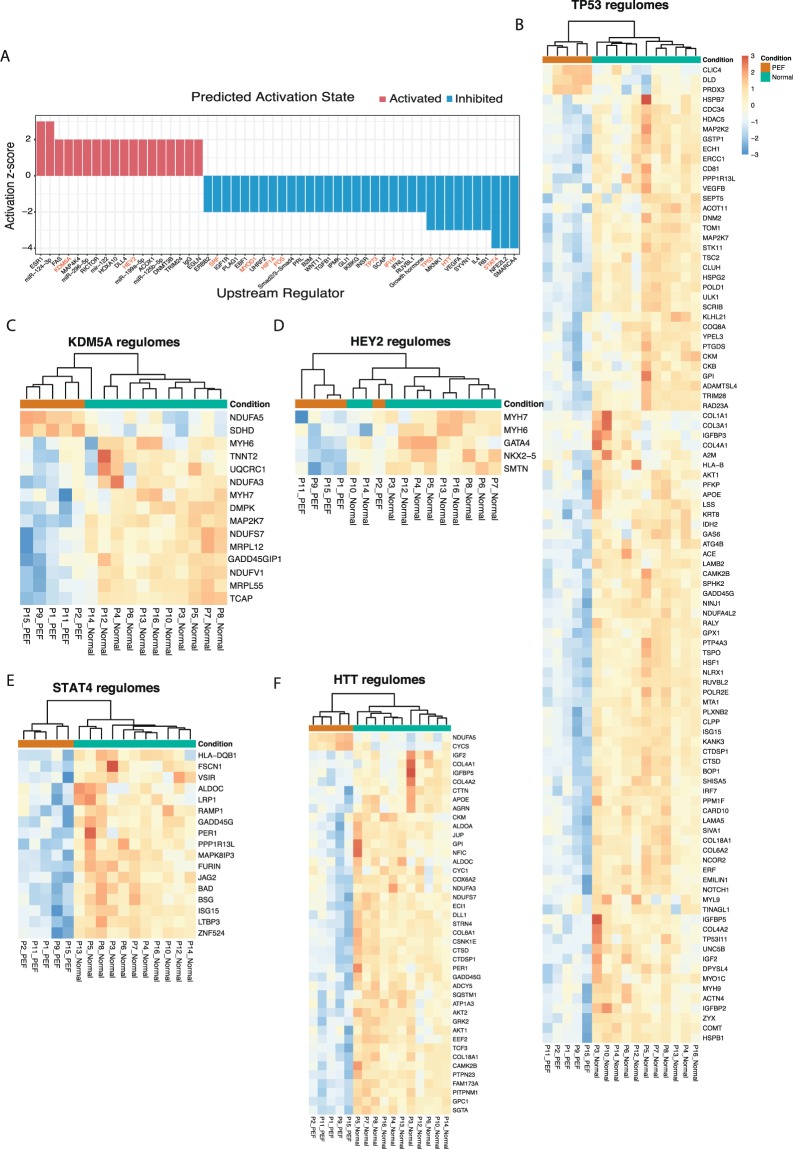
Predicted upstream regulators and gene expression of their regulomes. (**A**) Bar plot showing activation or inhibition scores of the upstream regulators. Transcription factors are highlighted in red. (**B**–**F**) Heat maps showing the expression profiles of the genes regulated by predicted transcription factors from A. Additional heat maps are shown in Supplementary Fig. S3. Samples in the HFpEF proxy group are coloured orange in Condition, and samples in the Normal physiology group are coloured green. The regulators genes were identified using IPA. Patient numbers and conditions are shown at the bottom of each heatmap.

The Ingenuity Pathways Analysis (IPA) analysis suggested that out of the total set of 736 DEGs (7 Ensembl gene IDs were not recognised by IPA and hence excluded from this analysis), 381 DEGs (52%) belong to known upstream regulators predicted to be activated or inhibited (activation Z-score ≥ ±2), at a Fisher’s Exact test p-value ≤ 0.05^18^. A comprehensive list of 52 upstream regulators along with predicted activation states is found in Supplementary Table S3. Of these regulators, 34 were predicted to be inhibited and 18 were to be activated (Fig. 4A).

Among the upstream regulators predicted in our analysis to be inhibited in the HFpEF proxy group were transcription factor *STAT4*, *HTT* and tumour suppressor *TP53* (Fig. 4), the latter regulating a total of 96 DE genes (13% of the DE gene set). Additional predicted inhibited upstream regulators included specific transcription factors (*SRF*, *MYOD1*, *FOS* and *HIF1A*; Supplementary Fig. S3). Furthermore, histone demethylase *KDM5A* and the basic helix–loop–helix (bHLH) type transcription factor *HEY2* (Fig. 4), both transcriptional repressors involved in Notch signalling, were among the identified activated upstream regulators.

**Figure 5:**
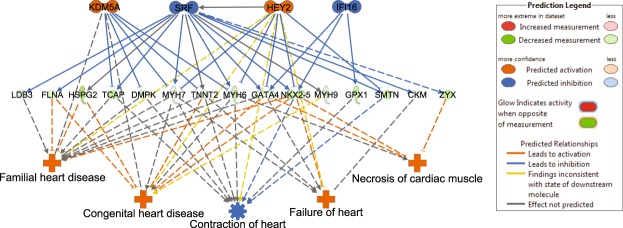
Transcription factor regulatory effect network identified using Ingenuity Pathway Analysis (IPA). In the network nodes, the upper panel shows transcription factors, the middle panel shows differentially expressed genes, and the lower panel shows biological functions and diseases. For the network edges, a solid line indicates direct interaction, while a dashed line indicate indirect interaction. Node colours in upper and lower panels: predicted activation in orange; predicted inhibition in blue. Node colours in middle panel: downregulated in data set coloured green; upregulated in data set coloured red (not represented in this network). Edge colours; predicted activation in orange; predicted inhibition in blue, findings inconsistent with state of downstream node in yellow; effect not predicted in grey.

Based on the predicted upstream regulator, a total of 8 regulatory effect networks were identified (Supplementary Table S4). These networks depict potential paths by which activation or inhibition of specific transcription factors lead to impaired cardiac function, heart failure and other heart diseases (networks 1–4,7), as well as impaired vessel formation/endothelial cell function (networks 3,5,6,8). The most causally consistent and densely connected network is shown in Fig. 5.

## Discussion

**Figure 6:**
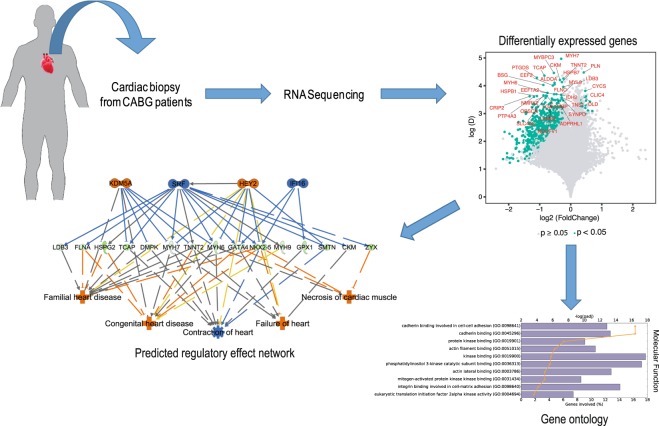
Schematic summary of the current study. Cardiac biopsies from CABG patients were submitted to RNA sequencing to detect differentially expressed genes between HFpEF and Normal. These differentially expressed genes were characterised using gene ontology and predicted transcription factor regulatory effect network.

To our knowledge, this is the first study using RNA-seq to identify dysregulated genes in patients with HFpEF characteristics, as schematically summarised in Fig. 6. In this exploratory translational study of elective CABG patients undergoing perioperative myocardial biopsies, we found that patients in the HFpEF proxy group displayed distinctive gene expression compared to patients with Normal physiology. The top biological functions associated with down-regulated genes in HFpEF proxy patients were cardiac muscle contraction, oxidative phosphorylation, endocytosis/cell remodelling, matrix organization and fibrosis. Further, genes regulated by transcription factor *STAT4* and tumour suppressor *TP53* were found to be down-regulated.

### Patients

The patients investigated in this study were the initial group in whom the myocardial biopsies were obtained within the ongoing CABG-PREFERS study^1^. They represent patients with a clinical indication for elective CABG. Hence, very few had a previous myocardial infarction or coronary intervention and few had a previous HF diagnosis. The three patients who had a HF diagnosis were all in the HFpEF proxy group.

### HFpEF

HFpEF is more frequent today, which may be due to increasing life span of the population, improved survival after myocardial infarction and increasing rates of HF risk factors like hypertension, overweight, and diabetes. However, the pathophysiology of this disease in not well understood at the transcriptome level. Already in the 1980s, it was recognised that ischemia might lead to diastolic dysfunction. We identified HFpEF characteristics in 31% of the group of patients planned for elective CABG, implying that other prevalent comorbidities except coronary artery disease, such as hypertension and diabetes may also play a role for development of HFpEF suggesting a link to microvascular dysfunction^19^.

### Imaging

HFpEF constitutes a diagnostic challenge and in an individual patient, there may be problematic measure overlaps and grey zones. Poor echocardiographic windows, tachyarrhythmias and atrial fibrillation makes measurements more difficult. The present guidelines advocate the use of at least 4 up to 8 parameters of structural LV dysfunction and diastolic dysfunction for diagnosis and risk prediction, some of these parameters may be used interchangeably^5,20^. In summary, the number of altered variables may increase the precision of the HFpEF diagnosis. In the current study we therefore used state-of-the-art guideline criteria for HFpEF and a majority of the 4–8 criteria achieved in an individual patient should be positive for rendering a HFpEF proxy diagnosis. Our definition is further strengthened by the fact that we used a consensus method in ambiguous patients. Although the five HFpEF proxy patients fulfilled the defined criteria they only had mild dysfunction and low NT-proBNP levels. However, we believe that such early stage HFpEF is highly relevant since these patients seem more sensitive to treatment with RAAS blockade^21^. Thus, RNA sequencing may be of particular value in early or mild HFpEF patients for finding new pathophysiologic translational mechanisms.

### Genes dysregulated in HFpEF proxy patients

To get an overview of the gene expression profiles, we performed PCA and OPLS-DA to distinguish HFpEF proxy patients from those with Normal physiology. Our results (Fig. 1C,D) suggest that the two groups have different gene expression profiles. The downregulated genes (Supplementary Table S1) were analysed with respect to enrichment of GO terms (Fig. 3A,B), revealing that cardiac muscle contraction, oxidative phosphorylation, endocytosis and extracellular matrix organization were associated with the dysregulated genes (Fig. 3A, Supplementary Table S2). These first two GO terms were also found to be enriched in a mouse model of pathological hypertrophy^17^.

Downregulated cardiac contraction genes may correlate with impaired systolic pump function in HFpEF patients as has been described in previous studies^22^. The gene *TNNT2* encodes for the tropomyosin binding subunit in the troponin complex which is located in the thin filament of the striated muscle and regulates muscle contraction in response to alteration in the intracellular calcium ion concentration^23^. However, we found no differences between the two groups for measures of systolic function at rest. EF and global longitudinal strain (GLS) were similar in the two groups. The described downregulated genes for myocardial contraction may be related to more long-term changes caused by passive cardiac stiffness by fibrosis or oxidative titin changes^12^. Short-time adaptations of systolic function, cardiac relaxation and diastolic function may counteract long-term adaptations and could be caused by other regulatory physiological mechanisms rather than transcriptome proliferative changes.

Our study shows that *RASD1*, coding for ras-related dexamethasone-induced protein 1, is downregulated in HFpEF proxy patients. *RASD1* is a modulator of cardiac endocrine function in response to volume overload underlying anti-natriuretic peptide excretion such as atrial natriuretic peptide (ANP) and brain natriuretic peptide (BNP)^24^. *RASD1* has also been shown to be downregulated in volume overloaded rat hearts^24^.

Furthermore, *PLN*, coding for phospholamban was upregulated in HFpEF proxy patients. PLN is a pentamer and a major substrate for the c-AMP dependent kinase in cardiac muscle. Phospholamban modulates the activity of the sarcoplasmatic reticulum ATPase type 2 (SERCA 2a), which in turn modulates Ca^2+^ handling by the sarcoplasmatic reticulum and increases both contractility and relaxation, at least in studies of electric cardiac contractility modulation (CCM) therapy^25^. In contrast, these genes have been seen to be downregulated in HFpEF^26^.

During the cardiac cycle there is an active coupling between contractility during systole and relaxation during early diastole. Cardiac contraction and relaxation are both closely linked active energy dependent processes, i.e. in the ischemic cascade. The finding of downregulated genes in the HFpEF proxy group for oxidative phosphorylation and energy supply may thus be considered crucial for development for HFpEF as regards both systolic and diastolic cardiac function and in relation to ischemia in these patients. Further, transcriptional changes may develop more slowly and be counteracted by short-term alterations of contractility and relaxation by changes in neurohormonal activation and sympathetic tone, calcium fluxes, and ischemia^27^. Sympathetic tone is increased in both aging and heart failure, by increasing circulating catecholamines but also decreased β-adrenoreceptor sensitivity^28^. Calcium handling in diastole is essential to remove Ca^2+^ from the cytosol to ensure cardiomyocyte relaxation by SERCA back into the Sarcoplasmatic reticulum^29^. Traditional RAAS- and betablockade does not show benefits in HFpEF, but new knowledge of role of β-arrestins and G-protein coupled receptor kinases (GRKs) may open new targets for treatment of HFpEF^30^.

Our results show several downregulated genes involved in extracellular matrix assembly, which may influence remodelling and dilatation of the heart rather than increasing passive myocardial stiffness as shown for HFpEF^12^ due to increased synthesis of collagens typically with predominance for collagen I and III in the myocardium. This finding is therefore surprising and counterintuitive. However, the process of myocardial fibrosis is complex and involves dynamics of fibrous tissue turnover including sevveral steps; active synthesis, crosslinking and active degrading of collagen^31^. The genes *LAMA5* and *LAMB2*, coding for two laminin subunits (α5 and β2), were also downregulated in HFpEF proxy patients. The secreted protein acidic and rich in cysteine (*SPARC*) is a matrix-cellular collagen binding protein serving a key role in collagen assembly into the extracellular matrix. Recent studies demonstrated that disruption of the *SPARC* gene is associated with decreased capacity to generate organised, mature collagen fibres^32^.

*ANKRD1* is a transcription factor known to interact with sarcomeric proteins in the myofibrillar stretch sensor system^33^. It has been observed that the expression of *ANKRD1* in both transcription and protein levels were increased in failing heart^15,34^. In our data we also see a trend of upregulated mRNA expression in the HFpEF proxy patients.

We find that the gene *LUM* coding for lumican is among the most up-regulated genes in the HFpEF proxy group compared to Normal physiology. Lumican is an extracellular matrix localised proteoglycan associated with inflammatory conditions known to bind collagen. In a recent study, cardiac lumican was increased in experimental and clinical HF^35^. This study also indicated that inflammatory and mechanical stimuli induce lumican production by cardiac fibroblasts indicating a role in HF development. However, we are unaware of whether lumican has previously been shown to be up-regulated in CABG and HFpEF patients.

### Upstream regulators and regulatory effect networks

The tumour suppressor *TP53* has recently been described as an important regulatory factor in the heart^36^. In our study, 96 DE genes (13%) belong to those regulated by *TP53* (Fig. 3B). It has been described to be increased in human dilated cardiomyopathy (DCM)^37^, suggesting that elevation of *TP53* also plays a key role in the common path toward heart failure. There are data showing that TP53 inhibits angiogenesis by suppressing HIF-1 resulting in myocardial hypoxia and cardiac dysfunction which may be a novel molecular mechanism underlying transition of cardiac hypertrophy to HF^38^.

*STAT4* is important in both innate and adaptive immune responses^39,40^. However, *STAT4* did not show transcriptional repression in the HFpEF proxy group, indicating that additional mechanisms, e.g. post-translational modifications, protein–protein interactions, might be involved in providing the inhibitory effect.

We further hypothesised a regulatory effect network model for mechanistic understanding of the disease and dysregulated genes using the IPA regulatory effect tool. The network illustrates potential mechanism(s) by which transcription regulator activation (*KDM5A*, *HEY)* and inhibition *(SRF*, *IFI16*) may lead to impaired cardiac function (Fig. 5).

*KDM5A* (retinoblastoma-binding protein 2/RBP2) encodes a histone demethylase that is part of the core Notch–RBP-J repressor complex^41^ and has implicated in transcriptional regulation of Hox genes and cytokines^42^. It also plays a role in tumour progression and selective inhibition blocks cancer cell growth^43^. Little is known regarding the role of this gene in heart. In a recent study, likely disease-causal *KDM5A* variants were uncovered in whole exome sequencing in patients with congenital heart disease (CHD)^44^.

*HEY2* encodes a basic helix-loop-helix (bHLH)-type transcription factor that is preferentially expressed in the developing and adult cardiovascular system^45^. It acts as a transcriptional repressor downstream of Notch signalling pathway^46^ and likely plays a central role in the cardiac transcriptional machinery^47^. For example, *HEY2* expression levels influence cardiac hypertrophy and the progression to heart failure in response to pressure overload through modulation of apoptosis and GATA4 activity^48^. Our network analysis results suggest that activation of *HEY2* may have contributed to cardiac dysfunction in HFpEF proxy via transcriptional repression of key cardiac transcription activators *GATA4* and *NKX2*.*5*, among others.

Serum response factor (*SRF*) is a central cardiac transcription factor required for appearance of beating sarcomeres in the heart^49^. Based on our analysis, inhibition of *SRF* with accompanied downregulation of its target genes (*GATA4*, *NKX2*.*5*, *MYH6*, *MYH7*, *TNNT2*, *TCAP* and others) could be one additional explanatory mechanism underlying cardiac dysfunction in the HFpEF proxy group.

Although the exact function of p53 and IFN-inducible gene *IFI16* is not currently known, it has been proposed to act as transcriptional repressor and tumour suppressor via activation of p53 signals and inflammasome^50,51^.

In addition to IPA, we also explored network-based approaches for transcription factors enrichment; ChEA-db which is transcription factor targets database inferred from integrating literature curated Chip-X data^52^ and transcription factor protein-protein interaction networks^53^. The enrichment analysis revealed *TP53*, *SMAD2*, *SMAD3* and *ESR1* among the enriched transcription factors which were also predicted by IPA as upstream regulators.

### Limitations and strengths

The strength of this study consists of the revealed gene expression differences between HFpEF and Normal groups found in myocardial biopsies. However, there are some limitations in the study, such as relatively small number of patients used and unequal distribution between HFpEF and Normal. In order to obtain myocardial biopsies, we have chosen patients undergoing elective CABG enabling us to safely obtain tissue samples. Based on data from the SWEDEHEART (Swedish Web-system for Enhancement and Development of Evidence-based care in Heart disease Evaluated According to Recommended Therapies) registry we knew that around 20–30% of elelective CABG patients had LVEF ≥ 45%. To identify the HFpEF group, we used echocardiographic evaluation according to current international Guidelines. We could confirm that a proportion of patients indeed had HFpEF characterstics. Due to limited amounts of biopsy material, we had to use the entire biopsy to get deeper RNA sequencing for better sensitivity. Consequently, we were unable to perform any validation experiments which may be considered a limitation. A larger cohort of patients, which we aim at in the future, will add more insight into differences in gene expression between these two groups.

### Conclusions

This exploratory study could confirm our hypothesis that patients undergoing CABG with HFpEF characteristics compared to patients with Normal physiology had distinctive gene expression in cardiac biopsies with downregulated genes for myocardial contraction, energy supply, remodelling and fibrosis. We consider differences within these functional areas relevant for possible pathophysiological mechanisms in HFpEF. However, down-regulation of these functions in patients with HFpEF characteristics is complex to describe and understand. Our findings lend to support our further studies in a larger patient cohort to find pathophysiological mechanisms that can explain and ultimately lead to treatment for HFpEF.

## Methods

### Patients

Patients enrolled were scheduled for elective CABG without concomitant valve surgery and with preserved LVEF. They all had angina pectoris with or without a previous myocardial infarction. Cardiac biopsies were obtained during CABG for analysis of mRNA expression in the myocardial tissue. All patients were assessed at a baseline visit 4–8 weeks prior to CABG by clinical characteristics, echocardiography and blood sampling including natriuretic peptides. From the ongoing study CABG-PREFERS^1^ we now report data from the initial patients.

*Descriptive data* are presented as median and quartiles (Q1;Q3) or number (%), and comparisons between groups were performed by Wilcoxon rank sum test and Fisher’s exact test as appropriate.

### Definitions

Preserved LVEF was defined at the time of study design as LVEF ≥ 45%^1^. The patients were divided into two groups according to echocardiography, NTproBNP levels and HF guidelines definitions^5^. The group with echocardiographic characteristics and increased NTproBNP levels indicative of HFpEF^1,5^ was called *HFpEF proxy* for the purpose of this study and was used as a representative for HFpEF even when not showing signs or symptoms of heart failure. The *Normal physiology* group had LVEF ≥ 45% and no echocardiographic signs of HFpEF^54^.

The HFpEF proxy definition was based on LVEF ≥ 45% and the combination of the following five echocardiographic criteria; 1. left atrial volume indexed for body surface area (LAVI) > 34 ml/m^2^, 2. LV mass index ≥115 g/m^2^ for males or ≥95 g/m^2^ for females, 3. ratio of early mitral inflow wave velocity (E) to myocardial tissue early diastolic wave velocity (e′) defined as E/e′ ≥ 8, 4. e′ septal <0.07 m/s or e′ mean septal/lateral <0.09 m/s, 5. tricuspid regurgitation velocity >2.8 m/s, and additionally NTproBNP > 125 ng/L. Three abnormal criteria of these five were required to fulfil the definition (majority rule). In equivocal cases, classification was performed by consensus of two experts (M.J.E. and H.P., blinded for clinical characteristics) in line with previous experiences from the CHARM substudy^20^.

### Echocardiography

Transthoracic Doppler echocardiography was performed according to guidelines as previously reported^1^. A Vivid 9 ultrasound system (Vingmed-General Electric, Horten, Norway) was used in all studies. Images were digitally stored on a dedicated server, and data analysis was performed offline on the EchoPAC workstation (GE EchoPAC sw only, Norway) by one experienced sonographer. The mean value of 3 cardiac cycles was calculated for each variable.

### Tissue collection

From patients undergoing CABG, core needle biopsies were taken from the lateral wall of the left ventricle before initiation of cardiac arrest and stored in −70 °C as previously described^1^ and used for mRNA analysis. Patients were prepared for surgery according to standard clinical routines with placement of a central venous line in the internal jugular vein, an arterial line in the distal radial artery and a peripheral venous line in the brachial vein. A midline sternotomy was performed and one or two mammary arteries were procured for usage as conduits^55^.

### RNA extraction and sequencing

Total RNA was extracted using the RNeasy Fibrous Tissue Mini Kit (#74704, Qiagen). RNA libraries for sequencing were prepared using poly-A selection and the Illumina RNA strand-specific TruSeq Stranded mRNA Sample prep kit with 96 dual indexes (Illumina, CA, USA) according to the manufacturer’s instructions with the following changes. The protocols were automated using an Agilent NGS workstation (Agilent, CA, USA) using purification steps as described^56,57^.

Clustering was done by ‘cBot’ and samples were sequenced on HiSeq. 2500 (HiSeq Control Software 2.2.58/RTA 1.18.64) with a 2 × 125/2 × 150 setup using ‘HiSeq SBS Kit v4’ chemistry. The Bcl to FastQ conversion was performed using bcl2fastq-v2.17.1.14 from the CASAVA software suite. The quality scale used was Sanger/phred33/Illumina 1.8 + .

### Analysis of transcriptome (RNA-Seq) data

Whole transcriptome sequencing was performed for each biopsy. Initial quality checking of the sequencing raw reads was performed to identify potential outliers before doing further analysis using FastQC. Sequencing paired-end reads were mapped towards the human reference genome (version GR38) using Star Aligner^58^ with default options, and Ensembl genome annotation (version 37) was used for subsequent analysis. Reads that mapped to the exons of the coding genes were counted using HTSeq.^59^. Genes with count values of zero (i.e. no read detected) in all samples were filtered out before further analysis. Genes were then categorised into different biotypes and distribution over the reference genomes was calculated. Count data was also investigated for several potential biases such as RNA-degradation, GC content etc. using NOISeq.^60^ package in R (http://www.R-project.org). Before normalisation, lowly expressed genes were filtered out using proportion test per condition and multiple testing correction as per NOIseq manual^60^. Normalisation of the count data was performed using the Trim mean of M-values (TMM) approach^61^.

*Batch correction* of the data was performed using ARSyNseq function of the NOISeq package^60^. Differential gene expression analysis was done using the function noiseqbio with parameters k = 0.5, norm = ‘n’, lc = 0, r = 20, adj = 1.5, a0per = 0.9, which is recommended for clinical RNAseq samples^60^. For NOISeq, we set the parameter ‘q’ to 0.95, corresponding to a false discovery rate (FDR) < 0.05. Analysis and plots were generated in R environment using ggplot2.

*Principal component analysis* (PCA) was performed on the log_2_ transformed values of normalised batch corrected expression values using “princomp” function in R environment.

*Orthogonal projections to latent structures discriminant analysis* (OPLS-DA) was performed using SIMCA v14.1 (Umetrics, Umeå, Sweden) on the already normalised and batch corrected data to identify genes showing high variation in a pairwise manner. The Pareto scaling method was used in this case, since it reduces the relative importance of large values, but keeps data structure partially intact, and we wanted to detect small to medium feature differences.

Clustering analysis of the differentially expressed genes (DEGs) was performed using unsupervised hierarchical clustering. Normalised expression data were standardised before plotting as heatmap using pheatmap package for R environment.

### Functional analysis of the dysregulated genes

Gene set enrichment analysis for Gene Ontology (GO) terms with focus on biological process (BP) and cellular component (CC) was performed for the DEGs (using gene symbols as input) using Enrichr^53^ with the probability density function as p-value model. The enrichment was tested using Fisher’s exact test with corrected p-value < 0.05. The DEGs were further analysed through the use of IPA^18^ (Ingenuity Pathways Analysis; QIAGEN Inc., https://www.qiagenbioinformatics.com/products/ingenuity-pathway-analysis). This tool uses the information in the Ingenuity® Knowledge Base to assess signalling and metabolic pathways, upstream regulators, regulatory effect networks, and disease and biological functions that are likely to be perturbed based on a data set of interest (in our case, the DEGs). The IPA upstream analysis^18^ was performed to predict which regulators (i.e. any gene, protein or miRNA) that are activated or inhibited based on a calculated Activation Z-score, to explain the observed DEG changes in HFpEF proxy group vs. Normal physiology group. The IPA regulatory effect network analysis generated hypotheses for how a phenotype, function or disease is regulated by activated or inhibited upstream regulators. In our study, regulatory effect network analysis was used to specifically study the impact of the identified upstream transcription factor regulators on downstream heart disease functions, given the observed gene expression changes in HFpEF proxy group vs. Normal physiology group.

### Ethics statement

This study was conducted according to the Declaration of Helsinki and approved by the regional Ethics Committee Stockholm. All patients were included following oral and written informed consent.

## Supplementary information

Supplementary Information

## Data Availability

RNA-seq data have been deposited in the EMBL-EBI ArrayExpress database (www.ebi.ac.uk/arrayexpress) under accession number E-MTAB-7454.
