# Supplementary material for: Transcriptomics of cardiac biopsies reveals differences in patients with or without diagnostic parameters for heart failure with preserved ejection fraction
Source: Sci Rep. 2019 Feb 28;9:3179. doi: 10.1038/s41598-019-39445-2 (PMC6395693; doi:10.1038/s41598-019-39445-2)
Supplement: Supplementary file 1 — Supplementary Information [file 41598_2019_39445_MOESM1_ESM.pdf]

Supplementary Materials for

Transcriptomics of cardiac biopsies reveals differences in patients with or without diagnostic parameters for heart failure with preserved ejection fraction

Sarbashis Das[1,*], Christoffer Frisk[1,*], Maria J Eriksson[2,3], Anna Walentinsson[4], Matthias Corbascio[3,5], Camilla Hage[6,7], Chanchal Kumar[4,8], Michaela Asp[9], Joakim Lundeberg[9], Eva Maret[2,3], Hans Persson[10,11], Cecilia Linde[6,7] and Bengt Persson[1,12]

[1]Department of Cell and Molecular Biology, Science for Life Laboratory, Uppsala University, S-751 24 Uppsala, Sweden; [2]Karolinska University Hospital, Department of Clinical Physiology, S-171 76 Stockholm, Sweden; [3]Karolinska Institutet, Department of Molecular Medicine and Surgery, S-171 77 Stockholm, Sweden; [4]Translational Sciences, Cardiovascular, Renal and Metabolic Diseases, IMED Biotech Unit, AstraZeneca, S-431 83 Gothenburg, Sweden; [5]Karolinska University Hospital, Department of Thoracic Surgery, S-171 76 Stockholm, Sweden; [6]Karolinska Institutet, Department of Medicine, S-171 77 Stockholm, Sweden; [7]Karolinska University Hospital, Heart and Vascular Theme, S-171 76 Stockholm, Sweden; [8]Integrated Cardio Metabolic Center (ICMC), Department of Medicine, Karolinska Institutet, S-141 57 Huddinge, Sweden; [9]Science for Life Laboratory, Royal Institute of Technology, S-171 21 Stockholm, Sweden; [10]Karolinska Institutet, Department of Clinical Sciences, Danderyd Hospital, S-182 88 Stockholm, Sweden; [11]Danderyd Hospital, Department of Cardiology, S-182 88 Stockholm, Sweden; [12]Department of Medical Biochemistry and Biophysics, Science for Life Laboratory, Karolinska Institutet, S-17177 Stockholm, Sweden

*These authors contributed equally to the work.

Supplementary Figure S1

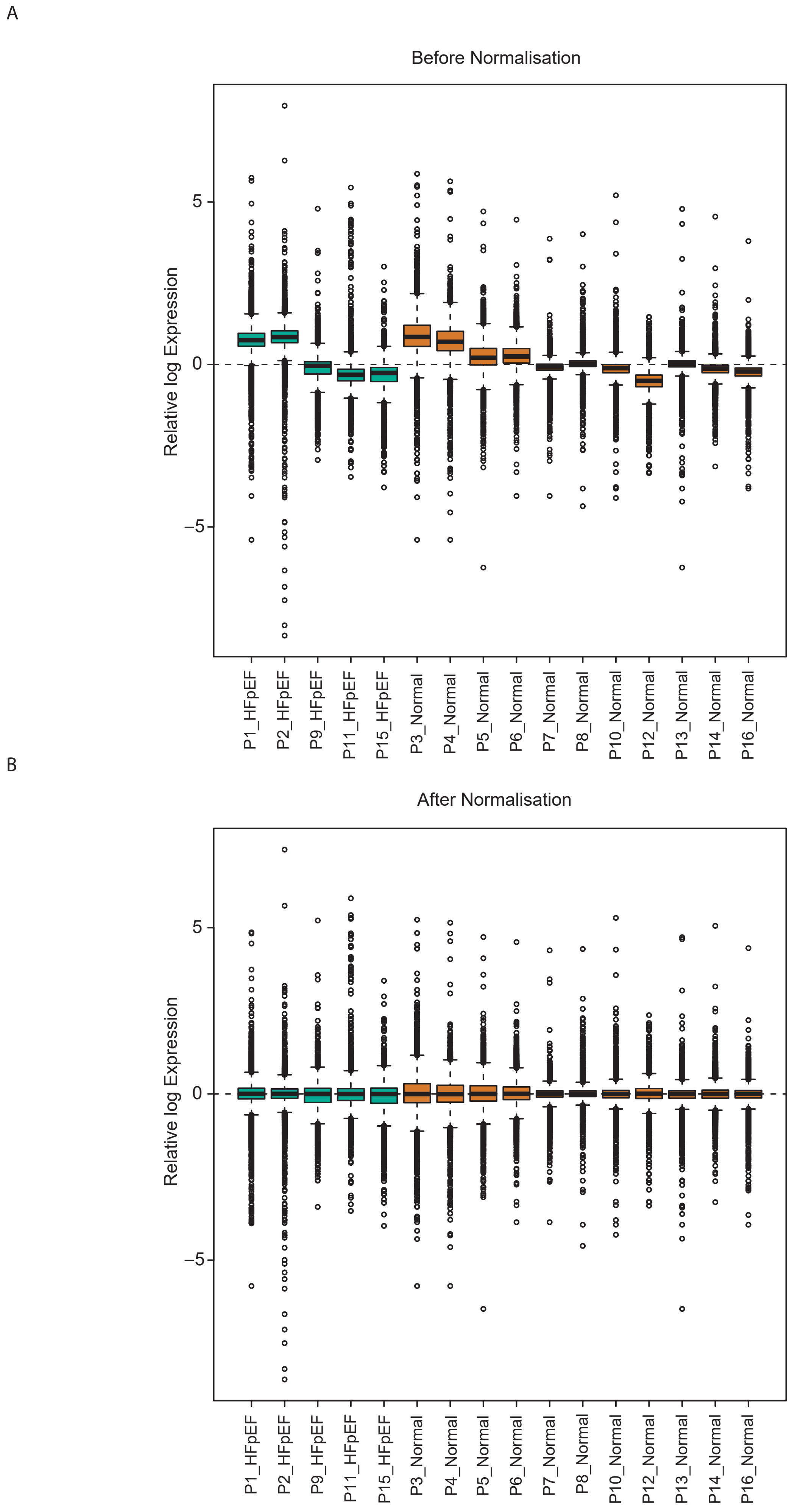

Supplementary Figure S1. Normalization of the raw count of the gene expression values. The graphs show before (top) and after (bottom) normalization. The x axis shows the samples and the y axis relative expression in log2 scale.

Supplementary Figure S2

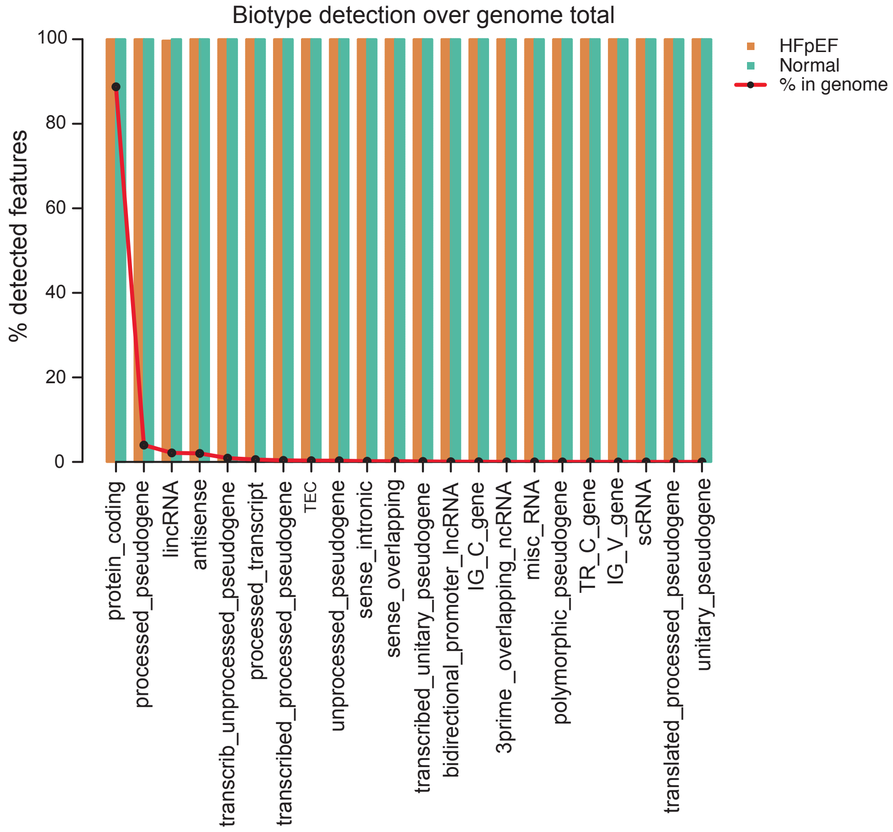

Supplementary Figure S2. Annotation overview and statistics of the genes identified by read alignment. Bar plot showing percentage of each biotype in the genome being detected in the sample. The x axis represents different biotypes and the y axis represents percentage of biotypes detected. The red line shows percentage of biotypes in the genome detected.

Supplementary Figure S3

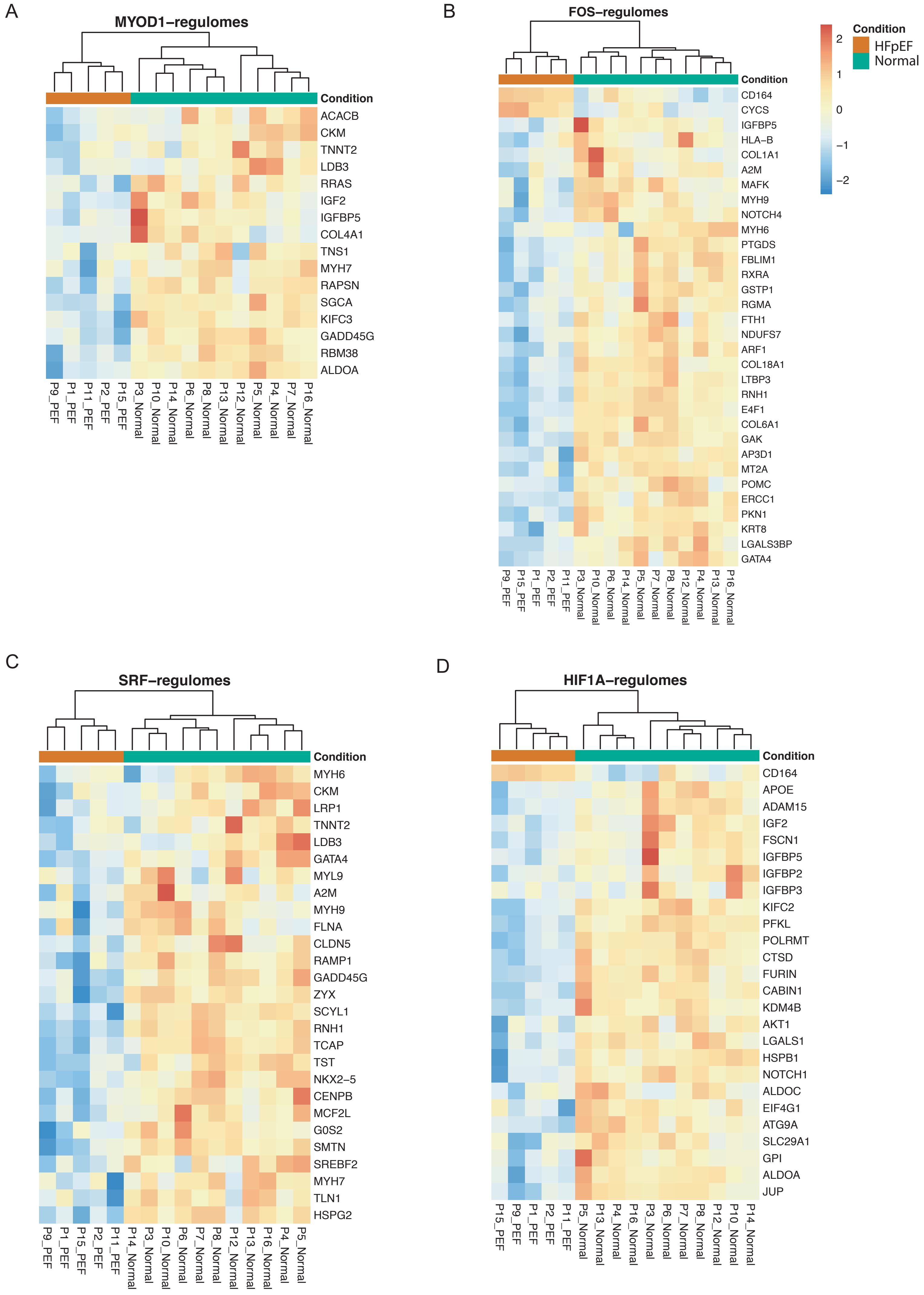

Supplementary Figure S3. Gene expression profiles of selected transcription factor regulons detected in differentially expressed genes. Heat maps showing the expression profiles of the genes regulated by predicted transcription factors by IPA similarly to those plotted in Figure 4B–F. A. *MYOD1*, B. *FOS*, C. *SRF*, and D. *HIF1A*. Samples were clustered using unsupervised hierarchical clustering approach.

Supplementary Tables

Supplementary Table S1. List of differentially expressed genes in HFpEF proxy compared with Normal

Row.names	HFPEF_mean	Normal_mean	theta	prob	log2FC	length	GC	Chrom	GeneStart	GeneEnd	Biotype	wikigene_description	percentage_gene_gc_content	hgnc_symbol
ENSG00000002330	21.80993167	74.51161079	-1.788930853	0.981277793	-1.772480001	14874	53.92	11	64269830	64284704	protein_coding	BCL2 associated agonist of cell death	53.92	BAD
ENSG00000004399	805.0686262	1260.536272	-1.493456863	0.976093705	-0.64685396	51643	58.39	3	129555175	129606818	protein_coding	plexin D1	58.39	PLXND1
ENSG00000005007	777.1690456	1152.935443	-1.554896963	0.977039249	-0.569011388	36298	55.16	19	18831938	18868236	protein_coding	UPF1, RNA helicase and ATPase	55.16	UPF1
ENSG00000007080	200.2813766	410.0623653	-1.894577307	0.987965885	-1.033815065	10975	55.84	19	17933016	17943991	protein_coding	coiled-coil domain containing 124	55.84	CCDC124
ENSG00000007376	34.18974402	93.50464307	-1.479599388	0.975927021	-1.451474384	3423	67.17	16	784974	788397	protein_coding	RNA pseudouridylate synthase domain containing 1	67.17	RPUSD1
ENSG00000007520	233.9440961	398.0809099	-1.292722978	0.954841918	-0.766897867	2671	62.57	16	1349240	1351911	protein_coding	TSR3, acp transferase ribosome maturation factor	62.57	TSR3
ENSG00000007541	108.5946677	218.197624	-1.403545454	0.967429404	-1.006682128	17141	64.36	16	566995	584136	protein_coding	phosphatidylinositol glycan anchor biosynthesis class Q	64.36	PIGQ
ENSG00000008710	691.2358133	1467.899421	-2.578550692	0.997359513	-1.086503248	47188	63.1	16	2088710	2135898	protein_coding	polycystin 1, transient receptor potential channel interacting	63.1	PKD1
ENSG00000010256	18200.37496	20575.46302	-1.30533203	0.956272831	-0.176956723	11974	53.04	3	48599002	48610976	protein_coding	ubiquinol-cytochrome c reductase core protein I	53.04	UQCRC1
ENSG00000010322	619.5853794	883.721521	-1.254584033	0.951235062	-0.512288717	37953	53.28	3	52455118	52493071	protein_coding	nischarin	53.28	NISCH
ENSG00000010327	229.9692945	462.8783141	-1.398690521	0.96688314	-1.009191729	29157	60.65	3	52495338	52524495	protein_coding	stabilin 1	60.65	STAB1
ENSG00000011009	258.3331941	434.44376	-1.360059662	0.963618684	-0.749936396	4569	62.39	1	23790970	23795539	protein_coding	lysophospholipase II	62.39	LYPLA2
ENSG00000011132	47.80072094	102.603018	-1.275955387	0.953003537	-1.101968885	10880	56.64	19	3750819	3761699	protein_coding	amyloid beta precursor protein binding family A member 3	56.64	APBA3
ENSG00000011451	274.4137391	564.0767808	-1.949962433	0.98709866	-1.039538838	29971	58.16	19	15419980	15449951	protein_coding	widely interspaced zinc finger motifs	58.16	WIZ
ENSG00000012061	1143.975582	1568.856501	-1.513255927	0.976091889	-0.45565714	71495	52.1	19	45407333	45478828	protein_coding	ERCC excision repair 1, endonuclease non-catalytic subunit	52.1	ERCC1
ENSG00000013306	496.8773748	785.7111742	-1.400065299	0.967030664	-0.661109228	5245	61.11	17	44319625	44324870	protein_coding	solute carrier family 25 member 39	61.11	SLC25A39
ENSG00000014164	77.43204448	159.5092809	-1.268203532	0.952239444	-1.042637728	103798	58.38	8	143437655	143541453	protein_coding	zinc finger CCCH-type containing 3	58.38	ZC3H3
ENSG00000014216	904.457128	1368.089783	-1.779388587	0.980166516	-0.597038887	31440	51.18	11	65180566	65212006	protein_coding	calpain 1	51.18	CAPN1
ENSG00000019144	1085.149618	1535.382915	-1.272555939	0.952656004	-0.500704528	51598	54.3	11	118606440	118658038	protein_coding	pleckstrin homology like domain family B member 1	54.3	PHLDB1
ENSG00000020129	407.4051048	696.3523708	-1.700337202	0.979086438	-0.773353472	9801	57.71	1	35557473	35567274	protein_coding	neurochondrin	57.71	NCDN
ENSG00000021762	121.4672354	229.9812262	-1.35721824	0.963308757	-0.920948883	79623	57	11	3087116	3166739	protein_coding	oxysterol binding protein like 5	57	OSBPL5
ENSG00000023191	796.5160855	1811.774459	-3.205423224	0.999875591	-1.185627969	12788	60.36	11	494512	507300	protein_coding	ribonuclease/angiogenin inhibitor 1	60.36	RNH1
ENSG00000024422	667.0388822	1022.354859	-1.4487681	0.973938537	-0.616053277	29791	52.96	19	47713343	47743134	protein_coding	EH domain containing 2	52.96	EHD2
ENSG00000030582	1267.659363	2205.871655	-2.639933568	0.989309819	-0.799181726	8016	57.64	17	44345086	44353102	protein_coding	granulin precursor	57.64	GRN
ENSG00000031823	546.5924173	781.1410037	-1.283208682	0.953784587	-0.515117547	62003	52.74	19	5916139	5978142	protein_coding	RAN binding protein 3	52.74	RANBP3
ENSG00000032444	217.1737774	411.4503628	-1.596471983	0.979688913	-0.92186848	27760	55.88	19	7534004	7561764	protein_coding	patatin like phospholipase domain containing 6	55.88	PNPLA6
ENSG00000039523	504.943722	821.0019814	-1.707707515	0.978935529	-0.701263101	28370	57.05	16	67518418	67546788	protein_coding	RHO family interacting cell polarization regulator 1	57.05	RIPOR1
ENSG00000050820	287.1381536	660.1705904	-2.026517964	0.988122745	-1.201093828	39866	59.7	16	75228187	75268053	protein_coding	BCAR1, Cas family scaffolding protein	59.7	BCAR1
ENSG00000053918	409.2266946	704.534876	-1.664730827	0.980948559	-0.783770867	404425	51.55	11	2444684	2849109	protein_coding	potassium voltage-gated channel subfamily Q member 1	51.55	KCNQ1
ENSG00000054148	1591.140211	2280.481281	-1.637711964	0.981501106	-0.519277356	2303	66.23	9	136848724	136851027	protein_coding	phosphohistidine phosphatase 1	66.23	PHPT1
ENSG00000055118	1200.030369	1922.292749	-1.927705649	0.988610202	-0.679757147	33354	59.57	7	150944961	150978315	protein_coding	potassium voltage-gated channel subfamily H member 2	59.57	KCNH2
ENSG00000058404	1677.8866	2440.859892	-1.828110292	0.985399428	-0.540744271	117427	55.17	7	44217150	44334577	protein_coding	calcium/calmodulin dependent protein kinase II beta	55.17	CAMK2B
ENSG00000058453	35.27328922	90.61235569	-1.283408265	0.953806555	-1.361131673	232706	49.14	1	16740273	16972979	protein_coding	ciliary rootlet coiled-coil, rootletin	49.14	CROCC
ENSG00000059122	256.3798074	505.3979481	-1.578436355	0.978527355	-0.979137168	39271	52.33	16	2911937	2951208	protein_coding	FLYWCH-type zinc finger 1	52.33	FLYWCH1
ENSG00000062822	29.62370708	66.68274823	-1.341261835	0.96120321	-1.170561375	33814	55.06	19	50384204	50418018	protein_coding	DNA polymerase delta 1, catalytic subunit	55.06	POLD1
ENSG00000063176	21.13079902	59.78918792	-1.468350267	0.975527635	-1.500537294	11426	56.78	19	48619291	48630717	protein_coding	sphingosine kinase 2	56.78	SPHK2
ENSG00000063245	656.0051527	1251.672359	-2.410375883	0.985134077	-0.932077916	34632	54.85	19	55675226	55709858	protein_coding	epsin 1	54.85	EPN1
ENSG00000063660	1648.553148	3436.185947	-3.612987458	1	-1.05960771	32407	64.63	2	240435671	240468078	protein_coding	glypican 1	64.63	GPC1
ENSG00000065000	994.2949778	1450.765296	-1.5856094	0.979006361	-0.545068315	63477	56.41	19	2100988	2164465	protein_coding	adaptor related protein complex 3 delta 1 subunit	56.41	AP3D1
ENSG00000065054	663.0991268	1768.324868	-2.908076174	1	-1.415086884	13670	64.6	16	2025356	2039026	protein_coding	SLC9A3 regulator 2	64.6	SLC9A3R2
ENSG00000065268	92.69718257	220.5181921	-1.492316537	0.976088812	-1.250300283	14167	62.29	19	984271	998438	protein_coding	WD repeat domain 18	62.29	WDR18
ENSG00000065717	297.6664143	494.5784323	-1.345343641	0.961789323	-0.732502876	49997	54.67	19	2997638	3047635	protein_coding	transducin like enhancer of split 2	54.67	TLE2
ENSG00000066056	413.7145913	753.3355745	-1.522513064	0.9761188	-0.864656818	22115	54.59	1	43300993	43323108	protein_coding	tyrosine kinase with immunoglobulin like and EGF like domains 1	54.59	TIE1
ENSG00000066735	66.31505017	160.1152925	-1.456475461	0.974716507	-1.271702874	42171	63.77	14	104138723	104180894	protein_coding	kinesin family member 26A	63.77	KIF26A
ENSG00000067057	2063.654869	3182.414225	-1.969806996	0.985758194	-0.624919919	71379	51.45	10	3066333	3137712	protein_coding	phosphofructokinase, platelet	51.45	PFKP
ENSG00000068001	384.3130762	758.9606417	-1.61378555	0.980638852	-0.981743007	5116	61.05	3	50317790	50322906	protein_coding	hyaluronoglucosaminidase 2	61.05	HYAL2
ENSG00000068137	110.0707846	287.1503417	-1.582314809	0.978788333	-1.383374684	9080	62	17	42667914	42676994	protein_coding	pleckstrin homology, MyTH4 and FERM domain containing H3	62	PLEKHH3
ENSG00000068976	2361.094273	3206.867778	-1.367883792	0.964364396	-0.441709225	13908	56.99	11	64746389	64760297	protein_coding	glycogen phosphorylase, muscle associated	56.99	PYGM
ENSG00000070047	194.2533302	363.1438351	-1.521322339	0.976111746	-0.902601757	35736	56.31	11	576486	612222	protein_coding	PHD and ring finger domains 1	56.31	PHRF1
ENSG00000070182	1294.246016	1833.988152	-1.275440975	0.95294983	-0.502872441	133600	48.21	14	64746283	64879883	protein_coding	spectrin beta, erythrocytic	48.21	SPTB
ENSG00000070413	977.3989562	1364.458661	-1.35396302	0.962926157	-0.481309217	86172	48.62	22	19036282	19122454	protein_coding	DiGeorge syndrome critical region gene 2	48.62	DGCR2
ENSG00000071564	113.4735382	213.4666356	-1.223686777	0.950142665	-0.911654694	43315	59.57	19	1609290	1652605	protein_coding	transcription factor 3	59.57	TCF3
ENSG00000071655	157.1281018	392.9466151	-1.904574828	0.988575582	-1.3223921	19205	58.04	19	1573596	1592801	protein_coding	methyl-CpG binding domain protein 3	58.04	MBD3
ENSG00000071889	397.8662662	609.9614607	-1.259509005	0.951538731	-0.616434509	10083	55.32	X	154506159	154516242	protein_coding	family with sequence similarity 3 member A	55.32	FAM3A
ENSG00000072163	1156.091788	2169.101473	-2.582260168	0.996947272	-0.907841601	43405	56.36	2	127638381	127681786	protein_coding	LIM zinc finger domain containing 2	56.36	LIMS2
ENSG00000072195	1245.590678	1805.96344	-1.475104793	0.975803969	-0.535938635	NA	NA	NA	NA	NA	NA	NA	NA	NA
ENSG00000072958	442.9212841	643.6532506	-1.232105158	0.950486108	-0.539233362	48329	51.59	19	16197578	16245907	protein_coding	adaptor related protein complex 1 mu 1 subunit	51.59	AP1M1
ENSG00000073169	53.71043841	124.5601931	-1.386729726	0.965803466	-1.213568683	16637	55.78	22	50200979	50217616	protein_coding	selenoprotein O	55.78	SELENOO
ENSG00000074181	817.0772873	1491.393255	-1.953164366	0.986846316	-0.868116267	41943	54.88	19	15159038	15200981	protein_coding	notch 3	54.88	NOTCH3
ENSG00000074416	2650.212381	3720.221799	-1.657857345	0.981268712	-0.489280659	134188	49.32	3	127689062	127823250	protein_coding	monoglyceride lipase	49.32	MGLL
ENSG00000074660	116.6502845	263.6630511	-1.511718804	0.976092248	-1.176505584	11889	58.07	17	1633858	1645747	protein_coding	scavenger receptor class F member 1	58.07	SCARF1
ENSG00000074964	875.8512938	1404.477965	-1.89105638	0.987703102	-0.681276142	158039	54.36	1	17539835	17697874	protein_coding	Rho guanine nucleotide exchange factor 10 like	54.36	ARHGEF10L
ENSG00000075399	71.75335189	143.3225673	-1.243048787	0.950775476	-0.998147659	13852	59.54	16	89707134	89720986	protein_coding	VPS9 domain containing 1	59.54	VPS9D1
ENSG00000075618	82.16251333	227.7939507	-1.487000073	0.97604837	-1.471177216	13832	59.62	7	5592823	5606655	protein_coding	fascin actin-bundling protein 1	59.62	FSCN1
ENSG00000075702	417.294685	995.8174965	-2.004666476	0.986249795	-1.254814818	50225	51.45	19	36054881	36105106	protein_coding	WD repeat domain 62	51.45	WDR62
ENSG00000076201	276.603419	441.154844	-1.242352558	0.950756268	-0.673466137	32430	51.53	3	47381011	47413441	protein_coding	protein tyrosine phosphatase, non-receptor type 23	51.53	PTPN23
ENSG00000076555	2044.859202	2794.180403	-1.406238725	0.96776178	-0.45042366	151631	46.78	12	109116595	109268226	protein_coding	acetyl-CoA carboxylase beta	46.78	ACACB
ENSG00000076924	212.9039915	402.1335911	-1.404825754	0.96758475	-0.917471855	10040	60.82	19	7619525	7629565	protein_coding	XPA binding protein 2	60.82	XAB2
ENSG00000076928	207.6081166	382.4999384	-1.282514802	0.953708353	-0.881596668	NA	NA	NA	NA	NA	NA	NA	NA	NA
ENSG00000076984	246.1665149	412.2541476	-1.343956685	0.961592771	-0.743899477	10635	63.14	19	7903843	7914478	protein_coding	mitogen-activated protein kinase kinase 7	63.14	MAP2K7
ENSG00000077009	5999.116626	10409.6062	-2.321644898	0.993383103	-0.795093508	9313	57.79	19	3933103	3942416	protein_coding	nicotinamide riboside kinase 2	57.79	NMRK2
ENSG00000078061	901.5722856	1213.323393	-1.293229783	0.954898569	-0.428449058	10820	51.23	X	47561100	47571920	protein_coding	A-Raf proto-oncogene, serine/threonine kinase	51.23	ARAF
ENSG00000078808	1094.579612	1653.97436	-1.810214924	0.984026209	-0.595559981	15123	61.35	1	1216908	1232031	protein_coding	stromal cell derived factor 4	61.35	SDF4
ENSG00000078814	2100.24386	3413.846998	-2.006627069	0.986396919	-0.700841551	27034	57.86	20	34975403	35002437	protein_coding	myosin heavy chain 7B	57.86	MYH7B
ENSG00000078902	576.0655274	847.580666	-1.310534952	0.956893467	-0.557117752	35283	57.49	11	1274371	1309654	protein_coding	toll interacting protein	57.49	TOLLIP
ENSG00000079308	16897.06158	20701.77224	-1.356416408	0.963217152	-0.292981897	203206	52.38	2	217799789	218002995	protein_coding	tensin 1	52.38	TNS1
ENSG00000079313	73.32165462	143.5030911	-1.309312731	0.956745131	-0.968770565	33217	60.94	19	1815246	1848463	protein_coding	RNA exonuclease 1 homolog	60.94	REXO1
ENSG00000079432	243.4649065	502.6598908	-1.637737072	0.98150151	-1.045868741	27260	61.52	19	42268537	42295797	protein_coding	capicua transcriptional repressor	61.52	CIC
ENSG00000079805	1683.190398	2262.744158	-1.601027952	0.97995571	-0.426875093	115409	53.07	19	10718079	10833488	protein_coding	dynamin 2	53.07	DNM2
ENSG00000079999	978.909072	1340.461	-1.259331344	0.951526327	-0.453482482	17621	54.01	19	10486120	10503741	protein_coding	kelch like ECH associated protein 1	54.01	KEAP1
ENSG00000084207	1862.571318	2968.822134	-2.097896405	0.987878202	-0.672594994	3065	63.05	11	67583595	67586660	protein_coding	glutathione S-transferase pi 1	63.05	GSTP1
ENSG00000085733	2146.358653	2842.385738	-1.422114152	0.970073195	-0.405211187	38180	52.81	11	70398404	70436584	protein_coding	cortactin	52.81	CTTN
ENSG00000085872	84.03435975	156.4375806	-1.281932831	0.953644606	-0.89653589	24641	57.3	19	16517889	16542530	protein_coding	calcium homeostasis endoplasmic reticulum protein	57.3	CHERP
ENSG00000086504	600.0078935	979.7432713	-1.667883203	0.980772494	-0.707422279	3143	64.85	16	367384	370527	protein_coding	mitochondrial ribosomal protein L28	64.85	MRPL28
ENSG00000087077	168.433216	349.2365316	-1.412487407	0.968610173	-1.052027805	6316	56.45	7	100867138	100873454	protein_coding	thyroid hormone receptor interactor 6	56.45	TRIP6
ENSG00000087087	495.9890044	739.8022798	-1.321009186	0.958248394	-0.576831609	13553	53.17	7	100875111	100888664	protein_coding	serrate, RNA effector molecule	53.17	SRRT
ENSG00000088256	492.3122375	931.711497	-1.929259928	0.988538894	-0.920309694	29594	61.66	19	3094410	3124004	protein_coding	G protein subunit alpha 11	61.66	GNA11
ENSG00000090006	1272.772816	2772.682323	-2.393058881	0.987663482	-1.123307403	36935	56.48	19	40592883	40629818	protein_coding	latent transforming growth factor beta binding protein 4	56.48	LTBP4

ENSG00000090013	705.5796127	1183.472149	-1.708242454	0.978928147	-0.746144974	18051	49.8	19	40447789	40465840	protein_coding	biliverdin reductase B	49.8	BLVRB
ENSG00000090238	286.0904636	550.7142087	-1.456122657	0.974685888	-0.944832423	4601	58.41	16	30092314	30096915	protein_coding	yippee like 3	58.41	YPEL3
ENSG00000090316	499.0677131	788.3571482	-1.600272716	0.979912327	-0.659613787	50296	55.03	4	1289851	1340147	protein_coding	macrophage erythroblast attacher	55.03	MAEA
ENSG00000090372	427.3300314	784.5405873	-1.857925951	0.985837684	-0.876497377	27487	57.35	19	46719507	46746994	protein_coding	striatin 4	57.35	STRN4
ENSG00000090975	263.9266344	446.4594367	-1.278375286	0.953259325	-0.758392156	166535	51.35	12	122983480	123150015	protein_coding	phosphatidylinositol transfer protein membrane associated 2	51.35	PITPNM2
ENSG00000091140	15163.67303	11236.12156	1.925538864	0.951353116	0.432475117	40760	36.67	7	107890970	107931730	protein_coding	dihydrolipoamide dehydrogenase	36.67	DLD
ENSG00000092054	395285.7725	488963.4154	-1.683696801	0.979808776	-0.306830496	22980	53.09	14	23412738	23435718	protein_coding	myosin heavy chain 7	53.09	MYH7
ENSG00000093010	328.8632259	605.744566	-1.529852039	0.976195392	-0.881221865	28368	53.41	22	19941607	19969975	protein_coding	catechol-O-methyltransferase	53.41	COMT
ENSG00000095066	410.2390997	649.9075265	-1.247903839	0.950932578	-0.663769455	109737	53.64	19	12763003	12872740	protein_coding	hook microtubule tethering protein 2	53.64	HOOK2
ENSG00000095321	1285.637204	1755.667117	-1.385218412	0.96568688	-0.449535745	16379	58.45	9	129094810	129111189	protein_coding	carnitine O-acetyltransferase	58.45	CRAT
ENSG00000095370	291.7793215	535.9700688	-1.439340827	0.972727893	-0.877274793	40424	53.99	9	127738317	127778741	protein_coding	SH2 domain containing 3C	53.99	SH2D3C
ENSG00000095906	126.9741386	335.3836956	-1.707353162	0.978940409	-1.401277868	6290	62.18	16	1782901	1789191	protein_coding	nucleotide binding protein 2	62.18	NUBP2
ENSG00000099624	628.0792942	1727.60521	-2.821149718	0.999896572	-1.459754959	3080	62.97	19	1241746	1244826	protein_coding	ATP synthase, H+ transporting, mitochondrial F1 complex, delta subunit	62.97	ATP5D
ENSG00000099795	1217.846229	2881.427077	-2.997080717	0.999992281	-1.242451525	5984	57.48	19	14566078	14572062	protein_coding	NADH:ubiquinone oxidoreductase subunit B7	57.48	NDUFB7
ENSG00000099800	263.6229261	518.4507244	-1.742404076	0.978792936	-0.975731036	2270	64.99	19	2425624	2427894	protein_coding	translocase of inner mitochondrial membrane 13	64.99	TIMM13
ENSG00000099804	570.1972961	873.4644508	-1.366654802	0.964256642	-0.61528779	10380	64.75	19	531712	542092	protein_coding	cell division cycle 34	64.75	CDC34
ENSG00000099817	772.6409171	1340.849272	-2.042876594	0.98907603	-0.795277083	8801	61.47	19	1086579	1095380	protein_coding	RNA polymerase II subunit E	61.47	POLR2E
ENSG00000099821	244.420264	501.5465871	-1.781108514	0.98034661	-1.037019816	16380	61.15	19	617224	633604	protein_coding	RNA polymerase mitochondrial	61.15	POLRMT
ENSG00000099864	143.0473113	277.8316025	-1.235281417	0.950574589	-0.95771833	39390	59.66	19	708939	748329	protein_coding	paralemmin	59.66	PALM
ENSG00000099875	1307.924	2011.709732	-1.867791222	0.986137582	-0.621143443	13779	64.23	19	2037465	2051244	protein_coding	MAP kinase interacting serine/threonine kinase 2	64.23	MKNK2
ENSG00000099889	154.9007749	329.7488253	-1.458156929	0.974859247	-1.090023159	46912	58.03	22	19969896	20016808	protein_coding	ARVCF, delta catenin family member	58.03	ARVCF
ENSG00000099904	120.8617851	294.2114098	-1.620630126	0.980960291	-1.283495041	18551	63.57	22	20129456	20148007	protein_coding	zinc finger DHHC-type containing 8	63.57	ZDHHC8
ENSG00000099917	306.4214442	521.8769618	-1.42003628	0.969746793	-0.768192451	91719	50.5	22	20495913	20587632	protein_coding	mediator complex subunit 15	50.5	MED15
ENSG00000099991	449.2043961	706.0649663	-1.423812249	0.970342173	-0.652428888	167436	50.24	22	24011192	24178628	protein_coding	calcineurin binding protein 1	50.24	CABIN1
ENSG00000100034	386.7661149	654.1822776	-1.451207084	0.974206106	-0.758231272	33417	55.32	22	21919420	21952837	protein_coding	protein phosphatase, Mg2+/Mn2+ dependent 1F	55.32	PPM1F
ENSG00000100065	190.2692219	342.9432495	-1.253630379	0.951184833	-0.849927649	29180	57.09	22	37490362	37519542	protein_coding	caspase recruitment domain family member 10	57.09	CARD10
ENSG00000100097	3409.657823	5557.674045	-2.629196493	0.99136163	-0.704854259	4198	56.78	22	37675608	37679806	protein_coding	galectin 1	56.78	LGALS1
ENSG00000100106	1137.910939	1748.909605	-2.026498533	0.988121153	-0.620068076	79552	54.35	22	37697004	37776556	protein_coding	TRIO and F-actin binding protein	54.35	TRIOBP
ENSG00000100116	442.3150557	701.3820737	-1.34978528	0.962395848	-0.665126208	9271	54.21	22	37807905	37817176	protein_coding	glycine C-acetyltransferase	54.21	GCAT
ENSG00000100239	698.2804907	1056.349632	-1.549497778	0.976774856	-0.597208848	101786	51.13	22	50343304	50445090	protein_coding	protein phosphatase 6 regulatory subunit 2	51.13	PPP6R2
ENSG00000100241	519.7597719	904.9423372	-1.773908708	0.979669168	-0.79998089	30024	60.67	22	50445000	50475024	protein_coding	SET binding factor 1	60.67	SBF1
ENSG00000100243	3484.31132	5190.799383	-2.472153388	0.990111724	-0.575083198	31728	55.99	22	42617840	42649568	protein_coding	cytochrome b5 reductase 3	55.99	CYB5R3
ENSG00000100258	143.9583733	321.8177976	-1.611324517	0.980514762	-1.160592409	4742	67.15	22	50502949	50507691	protein_coding	lipase maturation factor 2	67.15	LMF2
ENSG00000100263	76.06159486	150.7351631	-1.358648352	0.963467428	-0.98677591	8357	56.01	22	29259852	29268209	protein_coding	rhomboid domain containing 3	56.01	RHBDD3
ENSG00000100284	658.9363695	1042.064468	-1.610234126	0.980458659	-0.661233472	48719	53.51	22	35299275	35347994	protein_coding	target of myb1 membrane trafficking protein	53.51	TOM1
ENSG00000100299	149.8511162	286.4962073	-1.44242246	0.973151715	-0.93498621	5419	61.2	22	50622754	50628173	protein_coding	arylsulfatase A	61.2	ARSA
ENSG00000100300	361.1808406	780.0074956	-1.977661319	0.985488491	-1.110766623	11728	57.81	22	43151514	43163242	protein_coding	translocator protein	57.81	TSPO
ENSG00000100345	3062.804849	4238.849817	-1.598955057	0.979835834	-0.468819406	106737	52.37	22	36281281	36388018	protein_coding	myosin heavy chain 9	52.37	MYH9
ENSG00000100359	329.7426745	543.2377205	-1.293706403	0.954951847	-0.720243049	39698	47.84	22	40370591	40410289	protein_coding	small G protein signaling modulator 3	47.84	SGSM3
ENSG00000100399	38.58118069	128.5686966	-1.895864935	0.988057644	-1.736570225	11424	58.1	22	41229510	41240934	protein_coding	chondroadherin like	58.1	CHADL
ENSG00000100403	1063.71753	1484.985442	-1.474433072	0.975781053	-0.481333694	58625	54.29	22	41301522	41360147	protein_coding	zinc finger CCCH-type containing 7B	54.29	ZC3H7B
ENSG00000100605	1171.563379	1711.374235	-1.705326392	0.978973785	-0.546720273	179406	53.85	14	92936914	93116320	protein_coding	inositol-tetrakisphosphate 1-kinase	53.85	ITPK1
ENSG00000100726	105.4468192	270.0346696	-1.775882437	0.979834722	-1.356629069	17113	63.09	16	1493344	1510457	protein_coding	telomere maintenance 2	63.09	TELO2
ENSG00000100994	7379.617495	9789.673628	-1.712666854	0.978882289	-0.407714724	49945	52.17	20	25248069	25298014	protein_coding	glycogen phosphorylase B	52.17	PYGB
ENSG00000101199	223.940983	395.782787	-1.221808915	0.950031358	-0.821590292	17008	59.72	20	63272785	63289793	protein_coding	ADP ribosylation factor GTPase activating protein 1	59.72	ARFGAP1
ENSG00000101210	10978.86546	15931.08743	-3.04203563	0.99933123	-0.537115771	11302	63.5	20	63488013	63499315	protein_coding	eukaryotic translation elongation factor 1 alpha 2	63.5	EEF1A2
ENSG00000101246	383.856809	660.3948988	-1.705560216	0.978969454	-0.782760737	9383	62.43	20	63698642	63708025	protein_coding	ADP ribosylation factor related protein 1	62.43	ARFRP1
ENSG00000101335	19927.76173	24643.90356	-1.524367869	0.976132568	-0.306451118	9963	58.63	20	36541484	36551447	protein_coding	myosin light chain 9	58.63	MYL9
ENSG00000101400	3285.153559	5072.797873	-2.781621892	0.99760947	-0.626820869	35937	50.41	20	33407955	33443892	protein_coding	syntrophin alpha 1	50.41	SNTA1
ENSG00000101940	1075.882396	1617.531193	-1.847308409	0.985731019	-0.588273147	18825	51.72	X	48590042	48608867	protein_coding	WD repeat domain 13	51.72	WDR13
ENSG00000101986	108.4200301	198.2049233	-1.237344278	0.950627252	-0.870361486	19894	58.36	X	153724868	153744762	protein_coding	ATP binding cassette subfamily D member 1	58.36	ABCD1
ENSG00000102858	306.7197681	504.7535316	-1.282184996	0.95367221	-0.718657948	74481	55.34	16	4616493	4690974	protein_coding	mahogunin ring finger 1	55.34	MGRN1
ENSG00000102996	1043.968943	1740.943912	-1.68720797	0.979615916	-0.737790931	21335	59.51	16	58025566	58046901	protein_coding	matrix metallopeptidase 15	59.51	MMP15
ENSG00000103024	63.27630111	166.4212831	-1.351463535	0.962614174	-1.395102774	1444	69.2	16	1770286	1771730	protein_coding	NME/NM23 nucleoside diphosphate kinase 3	69.2	NME3
ENSG00000103145	377.3275835	644.3192034	-1.243114944	0.950777404	-0.771958025	1666	63.71	16	3022620	3024286	protein_coding	host cell factor C1 regulator 1	63.71	HCFC1R1
ENSG00000103197	545.447812	862.0687277	-1.459244424	0.974946934	-0.660361724	41255	60.06	16	2047465	2088720	protein_coding	TSC complex subunit 2	60.06	TSC2
ENSG00000103245	177.3840713	330.9471071	-1.349928512	0.962414883	-0.899724195	11576	60.82	16	729753	741329	protein_coding	nuclear prelamin A recognition factor like	60.82	NARFL
ENSG00000103254	19.45829532	49.1048061	-1.555007595	0.977045119	-1.335478909	2020	71.94	16	720581	722601	protein_coding	family with sequence similarity 173 member A	71.94	FAM173A
ENSG00000103266	135.7947659	248.6104444	-1.224581676	0.950189665	-0.872459033	2646	64.94	16	680224	682870	protein_coding	STIP1 homology and U-box containing protein 1	64.94	STUB1
ENSG00000103326	51.4953064	110.396544	-1.319378134	0.95802686	-1.100182161	26919	65.01	16	527717	554636	protein_coding	calpain 15	65.01	CAPN15
ENSG00000103335	107.9065899	226.8605374	-1.331111133	0.959695623	-1.0720227	69868	61.99	16	88715343	88785211	protein_coding	piezo type mechanosensitive ion channel component 1	61.99	PIEZO1
ENSG00000103363	1328.921023	2386.808413	-1.997570898	0.985803669	-0.844827398	5883	55.52	16	2771414	2777297	protein_coding	elongin B	55.52	ELOB
ENSG00000103507	1141.336679	1639.40546	-1.645973076	0.981541514	-0.522448277	6684	57.82	16	31106107	31112791	protein_coding	branched chain ketoacid dehydrogenase kinase	57.82	BCKDK
ENSG00000104805	1924.550781	2992.390823	-2.387756237	0.988454373	-0.636776873	23322	52.81	19	48900050	48923372	protein_coding	nucleobindin 1	52.81	NUCB1
ENSG00000104823	2434.68808	4642.425964	-3.259421813	0.999999826	-0.931141949	16583	51.47	19	38815422	38832005	protein_coding	enoyl-CoA hydratase 1	51.47	ECH1
ENSG00000104879	59186.84805	75298.96141	-1.703761518	0.979004179	-0.347353337	16563	53.07	19	45306414	45322977	protein_coding	creatine kinase, M-type	53.07	CKM
ENSG00000104881	714.1432738	1337.533017	-2.00362725	0.986175448	-0.905289058	26715	53.44	19	45379634	45406349	protein_coding	protein phosphatase 1 regulatory subunit 13 like	53.44	PPP1R13L
ENSG00000104897	88.85926965	192.6386503	-1.391926164	0.966232855	-1.116303	12175	58.67	19	2236504	2248679	protein_coding	splicing factor 3a subunit 2	58.67	SF3A2
ENSG00000104903	14.9606786	38.05048759	-1.255662724	0.951295931	-1.346739325	4128	62.1	19	13099033	13103161	protein_coding	LYL1, basic helix-loop-helix family member	62.1	LYL1
ENSG00000104904	5753.672194	6863.177877	-1.339470799	0.960939819	-0.254393718	3981	64.06	19	2269509	2273490	protein_coding	ornithine decarboxylase antizyme 1	64.06	OAZ1
ENSG00000104936	1512.86753	2405.271338	-2.199371722	0.985286543	-0.668913986	12835	59.04	19	45769717	45782552	protein_coding	DM1 protein kinase	59.04	DMPK
ENSG00000104969	836.6216775	1472.919826	-2.13591746	0.989685881	-0.816031619	28657	57.8	19	2754714	2783371	protein_coding	small glutamine rich tetratricopeptide repeat containing alpha	57.8	SGTA
ENSG00000104973	276.0811993	482.5961365	-1.362732861	0.963890431	-0.80572372	20537	56.01	19	49818279	49838816	protein_coding	mediator complex subunit 25	56.01	MED25
ENSG00000105063	410.6340171	704.4694634	-1.654031244	0.981399166	-0.778684026	29215	56.47	19	55229780	55258995	protein_coding	protein phosphatase 6 regulatory subunit 1	56.47	PPP6R1
ENSG00000105135	1326.599677	1991.381529	-1.717422157	0.978856105	-0.586036574	10801	57.25	19	15114984	15125785	protein_coding	ilvB acetolactate synthase like	57.25	ILVBL
ENSG00000105220	6681.164873	7866.03473	-1.417584392	0.969367354	-0.235536895	42676	52.45	19	34359480	34402156	protein_coding	glucose-6-phosphate isomerase	52.45	GPI
ENSG00000105221	1549.806496	2237.281731	-1.869927341	0.98623694	-0.529658844	55219	53.61	19	40230317	40285536	protein_coding	AKT serine/threonine kinase 2	53.61	AKT2
ENSG00000105223	1097.962995	1707.220884	-1.876314484	0.986604556	-0.636820298	31983	52.33	19	40348456	40380439	protein_coding	phospholipase D family member 3	52.33	PLD3
ENSG00000105325	197.7898851	341.3689148	-1.24750075	0.950917456	-0.787363043	32057	60.71	19	3506273	3538330	protein_coding	fizzy and cell division cycle 20 related 1	60.71	FZR1
ENSG00000105341	1741.430313	2303.67444	-1.518595117	0.976100125	-0.403664105	9399	51.01	19	41431318	41440717	protein_coding	distal membrane arm assembly complex 2	51.01	DMAC2
ENSG00000105355	176.7965151	305.2866116	-1.222195744	0.950056018	-0.788074484	29427	52.37	19	4838341	4867768	protein_coding	perilipin 3	52.37	PLIN3
ENSG00000105357	910.5032554	1601.347342	-2.100414634	0.987995564	-0.81455019	122359	50.8	19	50188186	50310545	protein_coding	myosin heavy chain 14	50.8	MYH14
ENSG00000105364	320.5290147	512.8684514	-1.387389719	0.965855475	-0.678133871	8144	56.13	19	10251901	10260045	protein_coding	mitochondrial ribosomal protein L4	56.13	MRPL4
ENSG00000105373	873.2857936	1398.310034	-1.5259194	0.976146824	-0.679158495	11536	57.2	19	47745522	47757058	protein_coding	NOP53 ribosome biogenesis factor	57.2	NOP53
ENSG00000105397	358.0469451	622.9903468	-1.492022356	0.976087302	-0.799061051	30147	54.34	19	10350529	10380676	protein_coding	tyrosine kinase 2	54.34	TYK2
ENSG00000105401	1536.827423	2117.995692	-1.690540207	0.979450379	-0.462744487	28987	54.04	19	10391134	10420121	protein_coding	cell division cycle 37	54.04	CDC37
ENSG00000105404	144.6990042	276.2136303	-1.350777025	0.962525528	-0.93272952	2709	61.62	19	41956681	41959390	protein_coding	Rab acceptor 1	61.62	RABAC1
ENSG00000105409	4328.377501	5997.614195	-1.834759567	0.985603683	-0.470562392	30915	56.13	19	41966582	41997497	protein_coding	ATPase Na+/K+ transporting subunit alpha 3	56.13	ATP1A3
ENSG00000105429	993.0006829	1505.166625	-1.784600277	0.980741511	-0.60005659	53160	54.75	19	42325609	42378769	protein_coding	multiple EGF like domains 8	54.75	MEGF8
ENSG00000105538	125.4991933	289.883293	-1.721812535	0.978844569	-1.207794097	20134	53.68	19	48720587	48740721	protein_coding	Ras interacting protein 1	53.68	RASIP1
ENSG00000105552	778.837993	1119.669159	-1.287426701	0.954251451	-0.523677338	15967	54.54	19	48795062	48811029	protein_coding	branched chain amino acid transaminase 2	54.54	BCAT2
ENSG00000105568	6177.970476	7539.663313	-1.693906176	0.979303258	-0.287367124	39494	48.38	19	52190039	52229533	protein_coding	protein phosphatase 2 scaffold subunit Aalpha	48.38	PPP2R1A
ENSG00000105669	315.2856568	575.5108831	-1.483147823	0.975996009	-0.868183671	19883	57.06	19	18899514	18919397	protein_coding	coatomer protein complex subunit epsilon	57.06	COPE
ENSG00000105701	1053.313601	2273.989639	-3.117563036	0.99039335	-1.110290649	12326	56.64	19	18531751	18544077	protein_coding	FK506 binding protein 8	56.64	FKBP8
ENSG00000105722	100.8734477	255.5202273	-1.795493605	0.982150521	-1.340891029	7585	64.09	19	42247572	42255157	protein_coding	ETS2 repressor factor	64.09	ERF
ENSG00000106009	89.89377268	208.257058	-1.479433474	0.975923206	-1.212072308	17850	55.18	7	2537877	2555727	protein_coding	BRCA1 associated ATM activator 1	55.18	BRAT1
ENSG00000106211	3768.408461	8011.465984	-3.48342287	1	-1.088110911	1751	63.41	7	76302544	76304295	protein_coding	heat shock protein family B (small) member 1	63.41	HSPB1
ENSG00000106400	512.7820645	772.7663318	-1.276370644	0.95304697	-0.591686438	6522	54.13	7	101217668	101224190	protein_coding	zinc finger HIT-type containing 1	54.13	ZNHIT1
ENSG00000107223	1600.614641	2219.030267	-1.652863581	0.98143154	-0.471303335	4167	57.77	9	136862119	136866286	protein_coding	endothelial differentiation related factor 1	57.77	EDF1
ENSG00000107263	1580.10196	2145.093237	-1.562195472	0.977457214	-0.441022701	163304	48.49	9	131576770	131740074	protein_coding	Rap guanine nucleotide exchange factor 1	48.49	RAPGEF1

ENSG00000107281	135.6241264	293.4834099	-1.400326047	0.967059286	-1.113665107	6733	65.86	9	137039470	137046203	protein_coding	neural proliferation, differentiation and control 1	65.86	NPDC1
ENSG00000107317	17168.71668	36561.56667	-3.95627702	1	-1.090545686	6650	66.35	9	136975092	136981742	protein_coding	prostaglandin D2 synthase	66.35	PTGDS
ENSG00000107331	378.2807529	652.1422761	-1.542249264	0.976490402	-0.785729374	21695	66.66	9	137007227	137028922	protein_coding	ATP binding cassette subfamily A member 2	66.66	ABCA2
ENSG00000107404	430.9735329	969.5206604	-2.446700304	0.984166063	-1.16967237	14074	66.25	1	1335276	1349350	protein_coding	dishevelled segment polarity protein 1	66.25	DVL1
ENSG00000107731	406.7827404	669.7005099	-1.422854791	0.970190328	-0.719257599	90294	54.37	10	71212570	71302864	protein_coding	unc-5 netrin receptor B	54.37	UNC5B
ENSG00000107738	872.1495794	1380.134907	-1.615857962	0.980740267	-0.662161804	25939	53.85	10	71747559	71773498	protein_coding	V-set immunoregulatory receptor	53.85	VSIR
ENSG00000107816	358.3032849	656.9068184	-1.620410206	0.980950578	-0.87450747	11218	60.49	10	100996618	101007836	protein_coding	leucine zipper tumor suppressor 2	60.49	LZTS2
ENSG00000108107	1556.689449	2222.951596	-1.496466577	0.976101825	-0.513995371	17905	52.88	19	55385345	55403250	protein_coding	ribosomal protein L28	52.88	RPL28
ENSG00000108262	406.5270778	648.6567763	-1.299065699	0.95555407	-0.674103857	20585	60.72	17	29573469	29594054	protein_coding	GIT ArfGAP 1	60.72	GIT1
ENSG00000108528	4137.016309	5335.187345	-1.741425693	0.978795706	-0.366948287	3121	58.68	17	4937130	4940251	protein_coding	solute carrier family 25 member 11	58.68	SLC25A11
ENSG00000108551	56.10627424	207.0236091	-1.754147467	0.97882649	-1.883561285	1958	62.89	17	17494437	17496395	protein_coding	ras related dexamethasone induced 1	62.89	RASD1
ENSG00000108557	233.4990585	473.8848155	-1.744408865	0.978788838	-1.021119702	129980	56.45	17	17681473	17811453	protein_coding	retinoic acid induced 1	56.45	RAI1
ENSG00000108639	1389.104107	2163.383766	-1.944266149	0.987549046	-0.639134883	4969	62.13	17	78168558	78173527	protein_coding	synaptogyrin 2	62.13	SYNGR2
ENSG00000108679	6102.670767	10405.14605	-3.156877994	0.996349494	-0.76978455	8871	58.85	17	78971238	78980109	protein_coding	galectin 3 binding protein	58.85	LGALS3BP
ENSG00000108821	566.5074052	1539.669788	-1.624279979	0.981113008	-1.442454251	18343	58.47	17	50183289	50201632	protein_coding	collagen type I alpha 1 chain	58.47	COL1A1
ENSG00000108823	1161.373228	2068.274114	-2.38751808	0.988489748	-0.83259572	11717	55.92	17	50164214	50175931	protein_coding	sarcoglycan alpha	55.92	SGCA
ENSG00000108840	1184.243174	1727.473955	-1.583830788	0.978889259	-0.544698603	46956	55.66	17	44076746	44123702	protein_coding	histone deacetylase 5	55.66	HDAC5
ENSG00000109107	2766.138409	3631.480837	-1.248381711	0.950950536	-0.392684621	4149	56.05	17	28573115	28577264	protein_coding	aldolase, fructose-bisphosphate C	56.05	ALDOC
ENSG00000109332	5075.290691	3986.679077	2.251285806	0.963533704	0.348302981	74513	38.48	4	102794383	102868896	protein_coding	ubiquitin conjugating enzyme E2 D3	38.48	UBE2D3
ENSG00000109501	1008.752784	1529.878866	-1.545802094	0.976619116	-0.600844772	33416	57.04	4	6269849	6303265	protein_coding	wolframin ER transmembrane glycoprotein	57.04	WFS1
ENSG00000109736	58.41042321	166.7444025	-1.672261214	0.980509285	-1.513340589	4298	67.55	4	2930561	2934859	protein_coding	major facilitator superfamily domain containing 10	67.55	MFSD10
ENSG00000110047	491.3181867	723.1430626	-1.281809537	0.953631119	-0.557623447	36654	54.84	11	64851642	64888296	protein_coding	EH domain containing 1	54.84	EHD1
ENSG00000110237	1504.42486	2166.528615	-1.89869505	0.988247791	-0.52617324	60802	55.27	11	73308289	73369091	protein_coding	Rho guanine nucleotide exchange factor 17	55.27	ARHGEF17
ENSG00000110628	76.26248812	171.7025413	-1.4585385	0.974890494	-1.170865886	25525	61.48	11	2899721	2925246	protein_coding	solute carrier family 22 member 18	61.48	SLC22A18
ENSG00000110651	6475.891777	8811.256746	-2.075534403	0.988076012	-0.444268929	21242	63.44	11	2376177	2397419	protein_coding	CD81 molecule	63.44	CD81
ENSG00000110697	232.3329106	500.5487698	-1.847606081	0.985732597	-1.107317111	14495	62.46	11	67491768	67506263	protein_coding	phosphatidylinositol transfer protein membrane associated 1	62.46	PITPNM1
ENSG00000110717	1188.477634	2289.217848	-2.427599472	0.983409967	-0.945740006	6027	57.63	11	68030617	68036644	protein_coding	NADH:ubiquinone oxidoreductase core subunit S8	57.63	NDUFS8
ENSG00000110799	5536.887357	7200.92606	-1.417064654	0.969288159	-0.379107281	175896	46.59	12	5948874	6124770	protein_coding	von Willebrand factor	46.59	VWF
ENSG00000111077	1244.629782	2053.289414	-1.885643193	0.987282793	-0.722220318	17403	60.28	12	53046969	53064372	protein_coding	tensin 2	60.28	TNS2
ENSG00000111676	1004.156333	1671.016418	-1.972309401	0.985651235	-0.734742014	17858	55.53	12	6924463	6942321	protein_coding	atrophin 1	55.53	ATN1
ENSG00000111897	9016.856328	6967.77029	2.287139266	0.975260273	0.371927472	28468	35.93	6	122443354	122471822	protein_coding	serine incorporator 1	35.93	SERINC1
ENSG00000112561	333.4564173	564.5778601	-1.29400689	0.954985487	-0.759674341	52281	54.14	6	41683978	41736259	protein_coding	transcription factor EB	54.14	TFEB
ENSG00000112759	2912.756012	3920.113429	-1.455357324	0.974615941	-0.428510541	14646	56.86	6	44219505	44234151	protein_coding	solute carrier family 29 member 1 (Augustine blood group)	56.86	SLC29A1
ENSG00000112787	258.1668161	530.964627	-1.870302683	0.986255205	-1.040312178	95637	62.75	12	132489551	132585188	protein_coding	fibrosin like 1	62.75	FBRSL1
ENSG00000113140	8939.669642	11813.90574	-1.44423239	0.973388856	-0.402192582	26069	47.91	5	151661096	151687165	protein_coding	secreted protein acidic and cysteine rich	47.91	SPARC
ENSG00000113504	2243.493732	3551.985797	-2.246523358	0.981189798	-0.66287866	61659	62.04	5	1050376	1112035	protein_coding	solute carrier family 12 member 7	62.04	SLC12A7
ENSG00000113558	4888.314299	3793.174991	2.178642742	0.972370006	0.365931118	28103	41.26	5	134148935	134177038	protein_coding	S-phase kinase associated protein 1	41.26	SKP1
ENSG00000113758	188.0694143	331.7953297	-1.253669089	0.951186868	-0.819028337	17793	59.54	5	177456608	177474401	protein_coding	drebrin 1	59.54	DBN1
ENSG00000114353	2185.774808	3140.015786	-2.184174339	0.986681336	-0.522627039	33063	57.36	3	50226292	50259355	protein_coding	G protein subunit alpha i2	57.36	GNAI2
ENSG00000114853	1357.598268	1902.251189	-1.503588105	0.976101613	-0.486651135	12170	62.11	3	42653684	42665854	protein_coding	zinc finger and BTB domain containing 47	62.11	ZBTB47
ENSG00000114867	4272.114894	5157.552135	-1.304034595	0.956121958	-0.271736052	20863	52.77	3	184314495	184335358	protein_coding	eukaryotic translation initiation factor 4 gamma 1	52.77	EIF4G1
ENSG00000114923	4857.027159	8515.745957	-3.621484274	1	-0.810059358	14653	60.88	2	219627327	219641980	protein_coding	solute carrier family 4 member 3	60.88	SLC4A3
ENSG00000115138	8.735083815	31.61742626	-1.567992568	0.977825812	-1.855826482	8050	51.16	2	25160853	25168903	protein_coding	proopiomelanocortin	51.16	POMC
ENSG00000115266	14.82374981	31.95429263	-1.264282652	0.951899326	-1.108099314	26942	63.5	19	1446302	1473244	protein_coding	APC2, WNT signaling pathway regulator	63.5	APC2
ENSG00000115268	802.0840602	1308.032137	-1.520362814	0.976106856	-0.705572639	2136	62.7	19	1438358	1440494	protein_coding	ribosomal protein S15	62.7	RPS15
ENSG00000115286	89.97727487	211.5457577	-1.330302258	0.959576047	-1.233337176	12062	59.57	19	1383527	1395589	protein_coding	NADH:ubiquinone oxidoreductase core subunit S7	59.57	NDUFS7
ENSG00000115457	390.0995062	733.5298899	-1.435100672	0.972108923	-0.911013582	31608	50.33	2	216632828	216664436	protein_coding	insulin like growth factor binding protein 2	50.33	IGFBP2
ENSG00000115461	7388.858571	10421.44604	-1.7242419	0.978840451	-0.496132054	23420	50.77	2	216672105	216695525	protein_coding	insulin like growth factor binding protein 5	50.77	IGFBP5
ENSG00000115648	663.8335109	948.6443814	-1.253181627	0.951161643	-0.515045905	69890	51.32	2	237485428	237555318	protein_coding	melanophilin	51.32	MLPH
ENSG00000116685	731.2474121	1002.274228	-1.235388512	0.950577409	-0.454845773	NA	NA	NA	NA	NA	NA	NA	NA	NA
ENSG00000116786	1108.680757	1755.724596	-1.979269352	0.985459619	-0.663222557	50437	52	1	15684332	15734769	protein_coding	pleckstrin homology and RUN domain containing M2	52	PLEKHM2
ENSG00000116809	163.475674	277.0764282	-1.234786366	0.95056157	-0.761208009	34263	51.22	1	15941869	15976132	protein_coding	zinc finger and BTB domain containing 17	51.22	ZBTB17
ENSG00000116871	3631.6337	4714.160314	-1.702584779	0.979029802	-0.376382127	25270	54.66	1	36155579	36180849	protein_coding	MAP7 domain containing 1	54.66	MAP7D1
ENSG00000117266	3367.562724	4910.319798	-2.365411753	0.991593631	-0.54411217	28198	55.71	1	205504595	205532793	protein_coding	cyclin dependent kinase 18	55.71	CDK18
ENSG00000117984	1421.389155	3074.474062	-3.307551381	1	-1.113038038	11821	64.73	11	1752752	1764573	protein_coding	cathepsin D	64.73	CTSD
ENSG00000118046	460.7436156	764.0512142	-1.542746367	0.976506955	-0.72970517	50877	57.49	19	1177558	1228435	protein_coding	serine/threonine kinase 11	57.49	STK11
ENSG00000118194	102274.5069	120402.0445	-1.464531747	0.97531588	-0.235413308	18754	54.6	1	201359008	201377762	protein_coding	troponin T2, cardiac type	54.6	TNNT2
ENSG00000119242	665.138042	955.0575351	-1.279647015	0.953395978	-0.52193386	54171	47.49	12	123918660	123972831	protein_coding	coiled-coil domain containing 92	47.49	CCDC92
ENSG00000119431	149.366954	318.1675048	-1.700113929	0.979092428	-1.090925496	3580	54.87	9	113373419	113376999	protein_coding	haloacid dehalogenase like hydrolase domain containing 3	54.87	HDHD3
ENSG00000119729	8651.960087	6641.047095	2.636300755	1	0.38161628	41315	44.36	2	46541806	46583121	protein_coding	ras homolog family member Q	44.36	RHOQ
ENSG00000120896	318.6342771	539.4484482	-1.359708867	0.963581451	-0.759583624	30802	55.84	8	22544986	22575788	protein_coding	sorbin and SH3 domain containing 3	55.84	SORBS3
ENSG00000122367	50602.94073	59695.34757	-1.587005114	0.97909748	-0.23839727	67619	53	10	86668449	86736068	protein_coding	LIM domain binding 3	53	LDB3
ENSG00000122386	12.35389683	44.77211542	-1.414233909	0.968864044	-1.857634298	7957	58.51	16	3112560	3120517	protein_coding	zinc finger protein 205	58.51	ZNF205
ENSG00000122390	376.6136567	613.7811099	-1.319506223	0.958044171	-0.704638929	43352	50.69	16	3443611	3486963	protein_coding	N(alpha)-acetyltransferase 60, NatF catalytic subunit	50.69	NAA60
ENSG00000122490	177.9650274	402.0556124	-1.801013781	0.982893551	-1.17580131	49244	52.94	18	79902420	79951664	protein_coding	PQ loop repeat containing 1	52.94	PQLC1
ENSG00000122515	426.3049232	705.1366329	-1.532322561	0.976235907	-0.726017116	21300	56.51	7	44748581	44769881	protein_coding	zinc finger MIZ-type containing 2	56.51	ZMIZ2
ENSG00000122971	1029.344267	2017.478811	-2.686190023	0.973569665	-0.970827945	14273	55.49	12	120725735	120740008	protein_coding	acyl-CoA dehydrogenase, C-2 to C-3 short chain	55.49	ACADS
ENSG00000123064	221.6389851	383.7617403	-1.267688468	0.95219275	-0.791999221	28305	54.08	12	113157174	113185479	protein_coding	DEAD-box helicase 54	54.08	DDX54
ENSG00000123143	1252.510713	1943.665786	-2.075537122	0.988075873	-0.633957225	38814	53.14	19	14433053	14471867	protein_coding	protein kinase N1	53.14	PKN1
ENSG00000123159	402.9796398	697.471018	-1.634758225	0.981445457	-0.79142632	18389	53.7	19	14477760	14496149	protein_coding	GIPC PDZ domain containing family member 1	53.7	GIPC1
ENSG00000123384	2038.035261	3257.160887	-1.828091916	0.985398683	-0.676435971	84858	56.25	12	57128493	57213351	protein_coding	LDL receptor related protein 1	56.25	LRP1
ENSG00000123689	733.0897622	1556.299287	-1.93270393	0.988355367	-1.086057764	968	57.59	1	209675420	209676388	protein_coding	G0/G1 switch 2	57.59	G0S2
ENSG00000123933	448.8802913	830.2079396	-1.782791072	0.98053214	-0.887141975	14862	62.91	4	2247432	2262294	protein_coding	MAX dimerization protein 4	62.91	MXD4
ENSG00000123989	609.5947859	1419.470221	-2.805910773	0.999587772	-1.219430115	4840	61.17	2	219538947	219543787	protein_coding	chondroitin polymerizing factor	61.17	CHPF
ENSG00000123999	12.13947829	30.82026191	-1.290929516	0.954641621	-1.3441727	6551	58.09	2	219569162	219575713	protein_coding	inhibin alpha subunit	58.09	INHA
ENSG00000124006	1427.237373	2659.468879	-2.68187692	0.974658394	-0.897912856	21130	58.29	2	219550729	219571859	protein_coding	obscurin like 1	58.29	OBSL1
ENSG00000124067	1195.360965	1638.378872	-1.370774093	0.964604535	-0.454822679	26127	56.91	16	67943474	67969601	protein_coding	solute carrier family 12 member 4	56.91	SLC12A4
ENSG00000125148	313.3040256	649.5801078	-1.517823664	0.976097736	-1.051944147	1298	59.05	16	56608199	56609497	protein_coding	metallothionein 2A	59.05	MT2A
ENSG00000125503	1098.30933	1749.090105	-1.675176993	0.980327648	-0.671320177	26646	57.6	19	55090913	55117559	protein_coding	protein phosphatase 1 regulatory subunit 12C	57.6	PPP1R12C
ENSG00000125520	509.7098952	795.4841843	-1.280192414	0.953455031	-0.642156886	3644	69.47	20	63739861	63743505	protein_coding	SLC2A4 regulator	69.47	SLC2A4RG
ENSG00000125534	608.8615419	1372.579389	-1.973718409	0.985599285	-1.172703501	1482	71.07	20	63520724	63522206	protein_coding		71.07	PPDPF
ENSG00000125652	438.4907917	721.9676838	-1.279773452	0.953409623	-0.719387715	2818	64.35	19	6372433	6375251	protein_coding	alkB homolog 7	64.35	ALKBH7
ENSG00000125656	286.9443693	470.772596	-1.299637388	0.955618788	-0.714259278	7456	54.14	19	6361452	6368908	protein_coding	caseinolytic mitochondrial matrix peptidase proteolytic subunit	54.14	CLPP
ENSG00000125817	732.4190387	1057.524097	-1.284944754	0.953976125	-0.529949338	2839	66.2	20	3783851	3786690	protein_coding	centromere protein B	66.2	CENPB
ENSG00000125826	492.4451492	783.8248318	-1.511678194	0.976092275	-0.670568239	23468	51.12	20	407498	430966	protein_coding	RANBP2-type and C3HC4-type zinc finger containing 1	51.12	RBCK1
ENSG00000125912	196.9798964	372.2095472	-1.496222243	0.976101429	-0.918066664	24012	60.96	19	3185563	3209575	protein_coding	nicalin	60.96	NCLN
ENSG00000125967	575.6810997	947.2133855	-1.624401295	0.981117837	-0.718419621	17376	59.54	20	33657087	33674463	protein_coding	N-terminal EF-hand calcium binding protein 3	59.54	NECAB3
ENSG00000125970	1079.139639	1484.185079	-1.476363995	0.975842501	-0.459789449	114662	44.86	20	33993646	34108308	protein_coding	RALY heterogeneous nuclear ribonucleoprotein	44.86	RALY
ENSG00000126217	409.1107219	686.9642828	-1.298373833	0.955475912	-0.747743744	205361	54.36	13	112894378	113099739	protein_coding	MCF.2 cell line derived transforming sequence like	54.36	MCF2L
ENSG00000126254	320.1517652	535.6937931	-1.274215946	0.952823745	-0.742652615	8656	53.79	19	35629030	35637686	protein_coding	RNA binding motif protein 42	53.79	RBM42
ENSG00000126457	1774.403958	2287.842926	-1.464255075	0.97529855	-0.366653517	13243	58.23	19	49675786	49689029	protein_coding	protein arginine methyltransferase 1	58.23	PRMT1
ENSG00000126458	889.7693439	1361.033595	-1.453597412	0.974449484	-0.61319938	4909	59.1	19	49635292	49640201	protein_coding	RAS related	59.1	RRAS
ENSG00000126461	181.6787657	400.6101662	-1.783979536	0.980668489	-1.140809223	16517	58.2	19	49642125	49658642	protein_coding	SR-related CTD associated factor 1	58.2	SCAF1
ENSG00000126464	119.4949182	236.2748912	-1.359351522	0.96354357	-0.983517058	34796	55.5	19	49591643	49626439	protein_coding	proline rich 12	55.5	PRR12
ENSG00000126603	83.24148517	173.5071861	-1.351042525	0.962559785	-1.059620805	24836	62.05	16	4314761	4339597	protein_coding	GLIS family zinc finger 2	62.05	GLIS2
ENSG00000126705	226.7250866	472.3960954	-1.735406523	0.978815478	-1.059053009	70396	56.39	1	27534035	27604431	protein_coding	AT-hook DNA binding motif containing 1	56.39	AHDC1
ENSG00000126895	33.8232927	85.22786301	-1.41255972	0.968620573	-1.333308046	4635	62.86	X	153902531	153907166	protein_coding	arginine vasopressin receptor 2	62.86	AVPR2
ENSG00000126934	831.3186928	1283.081826	-1.663809117	0.980996742	-0.626139622	33808	57.42	19	4090321	4124129	protein_coding	mitogen-activated protein kinase kinase 2	57.42	MAP2K2
ENSG00000127054	702.6042197	1039.643945	-1.562312237	0.977464389	-0.565305376	13106	60.46	1	1311585	1324691	protein_coding	integrator complex subunit 11	60.46	INTS11

ENSG00000127220	34.94785351	83.90365436	-1.515554165	0.976093514	-1.263529799	18105	54.9	19	17292131	17310236	protein_coding	abhydrolase domain containing 8	54.9	ABHD8
ENSG00000127445	461.2055591	717.8053097	-1.30022732	0.955685718	-0.638182693	14425	56.31	19	9835257	9849682	protein_coding	peptidylprolyl cis/trans isomerase, NIMA-interacting 1	56.31	PIN1
ENSG00000127533	8.931264348	50.66812144	-1.396630073	0.966672583	-2.504142012	3746	61.46	19	16888860	16892606	protein_coding	F2R like thrombin or trypsin receptor 3	61.46	F2RL3
ENSG00000127580	26.96030407	73.13245999	-1.541311036	0.976459486	-1.439675123	5822	63.87	16	684622	690444	protein_coding	WD repeat domain 24	63.87	WDR24
ENSG00000127663	295.6836772	493.3483108	-1.332650065	0.959924192	-0.738551965	184482	57.26	19	4969113	5153595	protein_coding	lysine demethylase 4B	57.26	KDM4B
ENSG00000128185	347.1704933	558.2694776	-1.353172216	0.962829813	-0.685317346	5804	57.73	22	20314276	20320080	protein_coding	DiGeorge syndrome critical region gene 6 like	57.73	DGCR6L
ENSG00000128274	434.7639542	724.1864346	-1.233015962	0.950513014	-0.73612882	29177	52.39	22	42692121	42721298	protein_coding	alpha 1,4-galactosyltransferase (P blood group)	52.39	A4GALT
ENSG00000128283	98.39594994	232.7142251	-1.850158755	0.985747571	-1.241888561	8958	58.87	22	37560447	37569405	protein_coding	CDC42 effector protein 1	58.87	CDC42EP1
ENSG00000128309	253.4236141	451.3773617	-1.379688739	0.965277287	-0.832783102	10187	55.28	22	37019635	37029822	protein_coding	mercaptopyruvate sulfurtransferase	55.28	MPST
ENSG00000128311	486.5514106	827.8364237	-1.513552937	0.976091996	-0.766753474	9324	57.04	22	37010859	37020183	protein_coding	thiosulfate sulfurtransferase	57.04	TST
ENSG00000128591	25774.36861	36944.90646	-1.455189521	0.974600498	-0.519438389	28897	60.86	7	128830377	128859274	protein_coding	filamin C	60.86	FLNC
ENSG00000128609	8053.502845	6060.828295	2.866477277	0.990996654	0.410101444	21258	35.76	7	123536997	123558255	protein_coding	NADH:ubiquinone oxidoreductase subunit A5	35.76	NDUFA5
ENSG00000128626	309.6755248	526.3040597	-1.435254868	0.972132259	-0.765139159	2614	55.6	19	38930548	38933162	protein_coding	mitochondrial ribosomal protein S12	55.6	MRPS12
ENSG00000129925	255.1612965	501.3448403	-1.721554711	0.978845096	-0.974393761	16340	61.61	16	370773	387113	protein_coding	transmembrane protein 8A	61.61	TMEM8A
ENSG00000129932	27.21970268	72.31325134	-1.550940153	0.976841329	-1.409608736	10118	56.59	19	3490822	3500940	protein_coding	deoxyhypusine hydroxylase	56.59	DOHH
ENSG00000129946	76.50081908	188.8758397	-1.483373608	0.975999708	-1.30389107	44413	58.69	19	416583	460996	protein_coding	SHC adaptor protein 2	58.69	SHC2
ENSG00000129968	117.6161333	342.5852664	-2.072587841	0.988231607	-1.54237714	8737	65.16	19	1876810	1885547	protein_coding	abhydrolase domain containing 17A	65.16	ABHD17A
ENSG00000130158	367.5449179	663.283315	-1.508648928	0.976094978	-0.851704662	63186	53.76	19	11199295	11262481	protein_coding	dedicator of cytokinesis 6	53.76	DOCK6
ENSG00000130159	1681.157727	2397.748442	-1.871894679	0.986339064	-0.512225222	23258	50.87	19	11505916	11529174	protein_coding	ECSIT signalling integrator	50.87	ECSIT
ENSG00000130202	286.2533519	530.4463851	-1.499340517	0.976104214	-0.889914351	43053	52.84	19	44846175	44889228	protein_coding	nectin cell adhesion molecule 2	52.84	NECTIN2
ENSG00000130203	114.9878758	310.9829542	-1.593903611	0.979533422	-1.435353751	3639	60.96	19	44905754	44909393	protein_coding	apolipoprotein E	60.96	APOE
ENSG00000130222	63.98581186	251.3046394	-1.847494947	0.985732008	-1.973613361	1542	66.23	9	89605013	89606555	protein_coding	growth arrest and DNA damage inducible gamma	66.23	GADD45G
ENSG00000130255	915.5014432	1504.370424	-1.574077618	0.97823224	-0.716525782	16929	58.81	19	5674947	5691876	protein_coding	ribosomal protein L36	58.81	RPL36
ENSG00000130303	408.393756	654.7175565	-1.321457495	0.958310082	-0.680911857	2709	58.38	19	17402939	17405648	protein_coding	bone marrow stromal cell antigen 2	58.38	BST2
ENSG00000130304	525.562828	912.2469513	-1.856216857	0.985809385	-0.795561184	37399	53.88	19	17468769	17506168	protein_coding	solute carrier family 27 member 1	53.88	SLC27A1
ENSG00000130312	624.4200277	1019.288787	-1.60400076	0.980123369	-0.70697414	14234	54.25	19	17292609	17306843	protein_coding	mitochondrial ribosomal protein L34	54.25	MRPL34
ENSG00000130382	376.7224025	589.910905	-1.293690419	0.954950059	-0.64699525	69569	55.57	19	6210379	6279948	protein_coding	MLLT1, super elongation complex subunit	55.57	MLLT1
ENSG00000130402	1066.46622	1582.697575	-1.542475907	0.976497888	-0.56954734	83934	53.01	19	38647649	38731583	protein_coding	actinin alpha 4	53.01	ACTN4
ENSG00000130508	509.3974956	866.8582207	-1.306992769	0.956468019	-0.767004186	112965	49.9	2	1631887	1744852	protein_coding	peroxidasin	49.9	PXDN
ENSG00000130511	41.47525006	107.5050111	-1.477560385	0.975876135	-1.374081325	15698	64.53	19	18418864	18434562	protein_coding	single stranded DNA binding protein 4	64.53	SSBP4
ENSG00000130529	253.2029538	435.3878647	-1.227619006	0.950329228	-0.782006961	54095	50.3	19	49157741	49211836	protein_coding	transient receptor potential cation channel subfamily M member 4	50.3	TRPM4
ENSG00000130560	1489.103805	1983.998412	-1.572462203	0.978123245	-0.413966543	28411	51.82	9	135932969	135961380	protein_coding	UBA domain containing 1	51.82	UBAC1
ENSG00000130589	80.93846184	186.7682421	-1.28038276	0.953475686	-1.206351824	16153	66.08	20	63558086	63574239	protein_coding	helicase with zinc finger 2	66.08	HELZ2
ENSG00000130590	37.02486475	77.32810035	-1.230014994	0.950419396	-1.062498162	5895	61.55	20	63974113	63980008	protein_coding	sterile alpha motif domain containing 10	61.55	SAMD10
ENSG00000130600	2445.207631	5781.744246	-2.340105737	0.993751842	-1.241547818	NA	NA	NA	NA	NA	NA	NA	NA	NA
ENSG00000130653	138.7356812	273.2400778	-1.342147382	0.961331996	-0.977830229	90581	54.81	9	137459953	137550534	protein_coding	patatin like phospholipase domain containing 7	54.81	PNPLA7
ENSG00000130702	306.3136444	782.9215127	-1.991396607	0.985548843	-1.353858054	59357	61.89	20	62307955	62367312	protein_coding	laminin subunit alpha 5	61.89	LAMA5
ENSG00000130723	1503.371811	2107.405775	-1.386643886	0.965796806	-0.48726627	106104	49.02	9	131394093	131500197	protein_coding	proline rich coiled-coil 2B	49.02	PRRC2B
ENSG00000130726	1066.085613	1652.242054	-1.850145487	0.98574748	-0.632101759	6631	59.3	19	58544091	58550722	protein_coding	tripartite motif containing 28	59.3	TRIM28
ENSG00000130734	276.7235942	469.593663	-1.447354349	0.973774	-0.762967283	9523	55.3	19	10543895	10553418	protein_coding	autophagy related 4D cysteine peptidase	55.3	ATG4D
ENSG00000130758	84.62061136	179.1771703	-1.473516263	0.97574938	-1.082305815	23831	52.96	19	40191744	40215575	protein_coding	mitogen-activated protein kinase kinase kinase 10	52.96	MAP3K10
ENSG00000130787	194.2286889	390.9144502	-1.508215439	0.976095528	-1.009096602	28507	59.16	12	122834453	122862960	protein_coding	huntingtin interacting protein 1 related	59.16	HIP1R
ENSG00000130821	2656.111419	3371.489486	-1.397831325	0.966794213	-0.344070432	8494	64.7	X	153688099	153696593	protein_coding	solute carrier family 6 member 8	64.7	SLC6A8
ENSG00000130881	98.76319702	205.7702355	-1.365624629	0.964163892	-1.05898887	31264	54.69	19	33177603	33208867	protein_coding	LDL receptor related protein 3	54.69	LRP3
ENSG00000131408	540.4405443	1034.537643	-2.153319152	0.988828972	-0.936778325	53329	51.27	19	50329653	50382982	protein_coding	nuclear receptor subfamily 1 group H member 2	51.27	NR1H2
ENSG00000131584	239.42706	452.1688676	-1.313231213	0.95722758	-0.91727545	17233	67.3	1	1292376	1309609	protein_coding	ArfGAP with coiled-coil, ankyrin repeat and PH domains 3	67.3	ACAP3
ENSG00000131669	385.0485261	594.3101351	-1.24987457	0.951010037	-0.62617571	12799	59.68	9	93121489	93134288	protein_coding	ninjurin 1	59.68	NINJ1
ENSG00000131899	187.6832585	314.254689	-1.293516709	0.954930631	-0.743634305	19288	59.89	17	18225587	18244875	protein_coding	LLGL1, scribble cell polarity complex component	59.89	LLGL1
ENSG00000132024	201.6962611	345.7333149	-1.315404589	0.957503979	-0.777475288	24678	53.88	19	13906201	13930879	protein_coding	coiled-coil and C2 domain containing 1A	53.88	CC2D1A
ENSG00000132153	841.6762918	1168.38377	-1.30036094	0.955700897	-0.473176838	47286	50.03	3	47802909	47850195	protein_coding	DExH-box helicase 30	50.03	DHX30
ENSG00000132329	586.3693107	1307.682455	-2.022448804	0.987774124	-1.157130751	53221	52.41	2	237858893	237912114	protein_coding	receptor activity modifying protein 1	52.41	RAMP1
ENSG00000132361	1634.264807	3033.428563	-2.932564855	1	-0.892307567	23277	62.54	17	2689386	2712663	protein_coding	clustered mitochondria homolog	62.54	CLUH
ENSG00000132471	2319.41167	3040.782894	-1.568363687	0.977850198	-0.390683909	10808	55.06	17	75845699	75856507	protein_coding	WW domain binding protein 2	55.06	WBP2
ENSG00000132819	403.7440359	669.2596815	-1.415583912	0.969064529	-0.729125156	17926	59.97	20	57391407	57409333	protein_coding	RNA binding motif protein 38	59.97	RBM38
ENSG00000133243	212.7138909	529.7328716	-2.068272866	0.988474088	-1.316350786	49443	55.3	19	1985438	2034881	protein_coding	BTB domain containing 2	55.3	BTBD2
ENSG00000133250	27.04917829	62.77821176	-1.381316308	0.965396201	-1.214679166	4486	62.76	19	8509678	8514164	protein_coding	zinc finger protein 414	62.76	ZNF414
ENSG00000133265	160.4781261	313.5723074	-1.498589043	0.976104121	-0.966421491	18150	51.91	19	55262231	55280381	protein_coding	HSPA (Hsp70) binding protein 1	51.91	HSPBP1
ENSG00000133275	125.7973015	283.8564891	-1.721942121	0.978844314	-1.174060747	40189	61.03	19	1941149	1981338	protein_coding	casein kinase 1 gamma 2	61.03	CSNK1G2
ENSG00000133315	532.8946887	865.6607997	-1.588558709	0.979197787	-0.699951377	167548	58.06	11	63998558	64166106	protein_coding	MACRO domain containing 1	58.06	MACROD1
ENSG00000133612	324.769171	510.3738558	-1.285374519	0.954023704	-0.652139738	58605	57.73	7	151085831	151144436	protein_coding	ArfGAP with GTPase domain, ankyrin repeat and PH domain 3	57.73	AGAP3
ENSG00000133619	261.4241802	481.059742	-1.337381856	0.960629873	-0.879823483	19794	57.32	7	149714781	149734575	protein_coding	KRAB-A domain containing 1	57.32	KRBA1
ENSG00000134463	1202.760437	1688.866241	-1.294284435	0.955016592	-0.489705753	21704	47.51	10	11742366	11764070	protein_coding	enoyl-CoA hydratase domain containing 3	47.51	ECHDC3
ENSG00000134571	63328.46339	92263.225	-3.81154677	1	-0.542901648	21305	57.91	11	47331397	47352702	protein_coding	myosin binding protein C, cardiac	57.91	MYBPC3
ENSG00000134590	2875.088391	4003.47756	-1.632242475	0.98138363	-0.477647409	1180	60.29	X	135032366	135033546	protein_coding	retrotransposon Gag like 8C	60.29	RTL8C
ENSG00000134775	7032.575406	8823.593192	-1.680282209	0.980011222	-0.327313161	482341	43.06	18	36297714	36780055	protein_coding	formin homology 2 domain containing 3	43.06	FHOD3
ENSG00000134871	2121.812676	3830.381535	-2.196650746	0.985588589	-0.852190809	207215	47.83	13	110305812	110513027	protein_coding	collagen type IV alpha 2 chain	47.83	COL4A2
ENSG00000135535	3375.820513	2338.442893	2.271190823	0.968391439	0.529690003	15943	38.11	6	109366514	109382457	protein_coding	CD164 molecule	38.11	CD164
ENSG00000136378	157.4147116	420.2442745	-1.749400282	0.97879215	-1.416657786	52228	56.03	15	78759203	78811431	protein_coding	ADAM metallopeptidase with thrombospondin type 1 motif 7	56.03	ADAMTS7
ENSG00000136574	2235.002337	2814.079612	-1.441530876	0.973031794	-0.332386804	83043	48.32	8	11676959	11760002	protein_coding	GATA binding protein 4	48.32	GATA4
ENSG00000136732	1370.764995	1794.57442	-1.242041547	0.950747735	-0.388660496	40742	50.2	2	126655933	126696675	protein_coding	glycophorin C (Gerbich blood group)	50.2	GYPC
ENSG00000136830	526.3597432	913.2339301	-1.805185168	0.983431186	-0.794935308	73650	54.31	9	127505339	127578989	protein_coding	family with sequence similarity 129 member B	54.31	FAM129B
ENSG00000136848	879.7517164	1364.15703	-1.732321686	0.978824641	-0.632841395	218473	53.14	9	121567057	121785530	protein_coding	DAB2 interacting protein	53.14	DAB2IP
ENSG00000137076	5540.885698	6860.400978	-1.502325016	0.97610291	-0.308176296	35447	51.19	9	35696948	35732395	protein_coding	talin 1	51.19	TLN1
ENSG00000137497	895.0278034	1325.414668	-1.455810966	0.974657733	-0.566439386	77829	46.62	11	72002864	72080693	protein_coding	nuclear mitotic apparatus protein 1	46.62	NUMA1
ENSG00000137818	6789.628675	9868.219635	-1.916137514	0.98886074	-0.53945715	3410	47.38	15	69452784	69456194	protein_coding	ribosomal protein lateral stalk subunit P1	47.38	RPLP1
ENSG00000138080	130.836442	316.9157018	-1.490158075	0.976076105	-1.276334709	7841	61.63	2	27078567	27086408	protein_coding	elastin microfibril interfacer 1	61.63	EMILIN1
ENSG00000138100	3255.518857	4517.982349	-2.705841116	0.972840807	-0.472791139	25047	47.7	2	27282392	27307439	protein_coding	tripartite motif containing 54	47.7	TRIM54
ENSG00000138834	491.6971433	896.4721215	-1.885287777	0.987255229	-0.866488746	64134	56.84	16	1706183	1770317	protein_coding	mitogen-activated protein kinase 8 interacting protein 3	56.84	MAPK8IP3
ENSG00000138835	1636.04144	2250.665872	-1.378125153	0.965163159	-0.460142603	153012	48.79	9	113444731	113597743	protein_coding	regulator of G protein signaling 3	48.79	RGS3
ENSG00000139329	7605.932989	4946.741701	2.578520244	0.99999992	0.620646659	9202	35.52	12	91102629	91111831	protein_coding	lumican	35.52	LUM
ENSG00000139722	150.1557461	262.3147261	-1.248216699	0.950944331	-0.804839117	31116	47.3	12	122865328	122896444	protein_coding	VPS37B, ESCRT-I subunit	47.3	VPS37B
ENSG00000140400	717.493933	1028.219623	-1.239842796	0.950689963	-0.519109912	13423	57.24	15	75355207	75368630	protein_coding	mannosidase alpha class 2C member 1	57.24	MAN2C1
ENSG00000140548	276.1215398	564.3008259	-1.583189496	0.9788465	-1.031161027	80814	50.7	15	90001392	90082206	protein_coding	zinc finger protein 710	50.7	ZNF710
ENSG00000140564	837.192787	1350.275829	-1.946947563	0.987338199	-0.689622358	14866	58.47	15	90868592	90883458	protein_coding	furin, paired basic amino acid cleaving enzyme	58.47	FURIN
ENSG00000140691	35.82089878	88.84946368	-1.342674935	0.961408695	-1.310561531	9086	58.18	16	31458080	31467166	protein_coding	armadillo repeat containing 5	58.18	ARMC5
ENSG00000140859	582.810476	1058.536604	-1.838151157	0.985660818	-0.860972444	104836	50.69	16	57758217	57863053	protein_coding	kinesin family member C3	50.69	KIFC3
ENSG00000140983	474.8806729	935.5399619	-1.95204096	0.986934259	-0.978234239	6088	66.37	16	668086	674174	protein_coding	ras homolog family member T2	66.37	RHOT2
ENSG00000140988	2883.143954	4015.653664	-1.382294833	0.965468091	-0.477991976	2808	57.46	16	1962052	1964860	protein_coding	ribosomal protein S2	57.46	RPS2
ENSG00000141524	306.2036255	779.2981863	-2.04294801	0.989077954	-1.347684094	21949	61.98	17	78110458	78132407	protein_coding	transmembrane channel like 6	61.98	TMC6
ENSG00000141552	639.2845043	1013.944963	-1.443118986	0.97324398	-0.665449315	10201	55.3	17	81890790	81900991	protein_coding	anaphase promoting complex subunit 11	55.3	ANAPC11
ENSG00000141564	269.2031561	447.3721081	-1.256857621	0.951366839	-0.732779988	421552	49.12	17	80544819	80966371	protein_coding	regulatory associated protein of MTOR complex 1	49.12	RPTOR
ENSG00000141905	1493.072834	2301.090084	-1.775690089	0.979818046	-0.62403292	154814	55.09	19	3314403	3469217	protein_coding	nuclear factor I C	55.09	NFIC
ENSG00000141959	399.4263966	799.8764393	-2.062227658	0.98879707	-1.001847479	27325	63.48	21	44300051	44327376	protein_coding	phosphofructokinase, liver type	63.48	PFKL
ENSG00000141985	102.701405	223.0218876	-1.62013534	0.98093827	-1.118729387	40177	57.17	19	4360370	4400547	protein_coding	SH3 domain containing GRB2 like 1, endophilin A2	57.17	SH3GL1
ENSG00000142089	1593.205294	2457.746495	-1.570370371	0.977983079	-0.625403937	7868	54.34	11	319669	327537	protein_coding	interferon induced transmembrane protein 3	54.34	IFITM3
ENSG00000142156	1346.500673	2569.250706	-2.507111059	0.9979417	-0.932132724	23313	64.67	21	45981737	46005050	protein_coding	collagen type VI alpha 1 chain	64.67	COL6A1
ENSG00000142173	1225.891338	2917.317612	-3.18593011	0.999354675	-1.250811358	34752	55.88	21	46098097	46132849	protein_coding	collagen type VI alpha 2 chain	55.88	COL6A2
ENSG00000142186	748.1347309	1074.212708	-1.408072299	0.968000282	-0.521909681	13627	57.59	11	65525077	65538704	protein_coding	SCY1 like pseudokinase 1	57.59	SCYL1
ENSG00000142208	1451.630162	1970.421631	-1.272553322	0.952655741	-0.440830432	26402	64.08	14	104769349	104795751	protein_coding	AKT serine/threonine kinase 1	64.08	AKT1
ENSG00000142327	729.1140003	1359.237297	-2.337791036	0.993807137	-0.898581036	15568	63.56	2	240565804	240581372	protein_coding	arginyl aminopeptidase like 1	63.56	RNPEPL1
ENSG00000142330	118.6625405	292.4917297	-1.654014911	0.98139962	-1.301531257	30989	54.59	2	240586716	240617705	protein_coding	calpain 10	54.59	CAPN10

ENSG00000142459	211.5131094	350.4321351	-1.267722318	0.952195777	-0.728387995	34743	58.04	19	7830233	7864976	protein_coding	ecotropic viral integration site 5 like	58.04	EVI5L
ENSG00000142634	144.2188861	277.419937	-1.3189516	0.957969801	-0.943811368	20448	56.77	1	15409895	15430343	protein_coding	EF-hand domain family member D2	56.77	EFHD2
ENSG00000142694	5.523855615	21.51610371	-1.869172008	0.98620043	-1.961669332	2123	70.2	1	36322031	36324154	protein_coding	eva-1 homolog B	70.2	EVA1B
ENSG00000142798	1632.070632	3778.780053	-3.388824075	1	-1.211217053	115052	56.09	1	21822245	21937297	protein_coding	heparan sulfate proteoglycan 2	56.09	HSPG2
ENSG00000142910	2175.426061	2875.902372	-1.32308008	0.958535204	-0.402716719	11201	55.48	1	31576485	31587686	protein_coding	tubulointerstitial nephritis antigen like 1	55.48	TINAGL1
ENSG00000143382	282.2470442	483.9848346	-1.324738097	0.958768916	-0.778003369	11568	60.13	1	150549369	150560937	protein_coding	ADAMTS like 4	60.13	ADAMTSL4
ENSG00000143434	890.6096509	1346.588512	-1.360950838	0.963711516	-0.596443912	14979	57.48	1	151131685	151146664	protein_coding	semaphorin 6C	57.48	SEMA6C
ENSG00000143537	974.2932538	1427.987074	-1.526600678	0.976153685	-0.551554938	12209	58.25	1	155050566	155062775	protein_coding	ADAM metallopeptidase domain 15	58.25	ADAM15
ENSG00000143742	4062.901893	2767.222037	2.230581283	0.965526517	0.55407212	12653	40.6	1	225777813	225790466	protein_coding	signal recognition particle 9	40.6	SRP9
ENSG00000143761	5389.075023	6550.906777	-2.045402813	0.989130275	-0.281656948	16552	52.33	1	228082660	228099212	protein_coding	ADP ribosylation factor 1	52.33	ARF1
ENSG00000143774	855.0107076	1535.385675	-1.892287436	0.987796984	-0.8445867	9022	61.76	1	228139962	228148984	protein_coding	guanylate kinase 1	61.76	GUK1
ENSG00000144230	45.23030266	150.563839	-1.400885942	0.967121936	-1.735013764	6775	59.4	2	127645864	127652639	protein_coding	G protein-coupled receptor 17	59.4	GPR17
ENSG00000144579	1180.638265	1625.794722	-1.566284011	0.977714595	-0.461578104	7685	63.19	2	218398256	218405941	protein_coding	CTD small phosphatase 1	63.19	CTDSP1
ENSG00000144711	1364.739461	1881.705728	-1.535650134	0.976304482	-0.463415472	175897	54.36	3	12897220	13073117	protein_coding	IQ motif and Sec7 domain 1	54.36	IQSEC1
ENSG00000144712	1019.722494	1415.860331	-1.293109457	0.954885134	-0.473502363	75444	50.87	3	12796472	12871916	protein_coding	cullin associated and neddylation dissociated 2 (putative)	50.87	CAND2
ENSG00000144746	8867.816004	6859.499572	2.690421475	1	0.370475506	21122	42.5	3	69084944	69106066	protein_coding	ADP ribosylation factor like GTPase 6 interacting protein 5	42.5	ARL6IP5
ENSG00000145214	41.54283442	105.8216837	-1.526424393	0.976151823	-1.34896372	28008	60.54	4	958887	986895	protein_coding	diacylglycerol kinase theta	60.54	DGKQ
ENSG00000145384	3.02733805	18.82135357	-1.449466469	0.974016663	-2.636248697	5140	32.06	4	119317250	119322390	protein_coding	fatty acid binding protein 2	32.06	FABP2
ENSG00000145494	1364.968252	1842.173414	-1.343921504	0.961587724	-0.432541481	15205	48.37	5	1801400	1816605	protein_coding	NADH:ubiquinone oxidoreductase subunit S6	48.37	NDUFS6
ENSG00000146540	389.5033434	778.2295427	-1.715280212	0.978865655	-0.998560036	141274	57.69	7	996986	1138260	protein_coding	chromosome 7 open reading frame 50	57.69	C7orf50
ENSG00000146674	1857.873138	2915.11817	-1.249842012	0.951008701	-0.649902374	9629	49.16	7	45912245	45921874	protein_coding	insulin like growth factor binding protein 3	49.16	IGFBP3
ENSG00000146729	14270.79657	11230.7012	2.350332426	0.977529353	0.345617859	48388	45.28	7	55951793	56000181	protein_coding	nipsnap homolog 2	45.28	NIPSNAP2
ENSG00000146830	339.097106	561.561549	-1.296416089	0.955255535	-0.727745684	9941	62.01	7	100679507	100689448	protein_coding	GRB10 interacting GYF protein 1	62.01	GIGYF1
ENSG00000146926	154.46346	367.1777626	-1.730574249	0.978828974	-1.249213094	12134	53.7	7	151175698	151187832	protein_coding	ankyrin repeat and SOCS box containing 10	53.7	ASB10
ENSG00000148334	1059.453646	1591.81633	-1.735959692	0.978813669	-0.587353415	7769	59.55	9	128120693	128128462	protein_coding	prostaglandin E synthase 2	59.55	PTGES2
ENSG00000148343	569.2874442	876.4948983	-1.446437673	0.973663475	-0.622588413	35461	53.65	9	129036621	129072082	protein_coding	mitoguardin 2	53.65	MIGA2
ENSG00000148400	158.8588605	350.0082302	-1.555256628	0.977058344	-1.139643287	51418	63.39	9	136494444	136545862	protein_coding	notch 1	63.39	NOTCH1
ENSG00000149091	315.9232371	516.7726162	-1.304799599	0.956210676	-0.709955568	47649	56.98	11	46332905	46380554	protein_coding	diacylglycerol kinase zeta	56.98	DGKZ
ENSG00000149564	953.7317283	1405.339049	-1.397822518	0.966793303	-0.559262816	9707	54.05	11	124752583	124762290	protein_coding	endothelial cell adhesion molecule	54.05	ESAM
ENSG00000149761	86.29403658	250.0057078	-1.85012387	0.985747331	-1.534628263	4745	59.06	11	64225941	64230686	protein_coding	nudix hydrolase 22	59.06	NUDT22
ENSG00000149809	979.3701909	1521.56542	-1.385942562	0.965742461	-0.635630173	4539	60.18	11	65111845	65116384	protein_coding	transmembrane 7 superfamily member 2	60.18	TM7SF2
ENSG00000149823	393.7756988	716.81149	-1.678266734	0.980134932	-0.864219682	22536	50.69	11	65089324	65111860	protein_coding	VPS51, GARP complex subunit	50.69	VPS51
ENSG00000149925	29433.8885	42239.32097	-3.262118189	0.999999884	-0.521108494	17367	54.43	16	30053090	30070457	protein_coding	aldolase, fructose-bisphosphate A	54.43	ALDOA
ENSG00000149930	560.0598435	856.5988335	-1.48581082	0.976034477	-0.613038722	18620	55.16	16	29973641	29992261	protein_coding	TAO kinase 2	55.16	TAOK2
ENSG00000151640	449.9948786	912.4199685	-1.740543162	0.978798375	-1.019789439	18876	61.67	10	132186900	132205776	protein_coding	dihydropyrimidinase like 4	61.67	DPYSL4
ENSG00000153130	3204.220809	1974.744735	2.222755177	0.967384149	0.698307394	128440	40.94	4	140257286	140385726	protein_coding	short coiled-coil protein	40.94	SCOC
ENSG00000153443	49.17421861	128.993238	-1.422610459	0.97015165	-1.391321406	6144	64.7	16	4608883	4615027	protein_coding	UBA like domain containing 1	64.7	UBALD1
ENSG00000153531	9288.209188	12453.40568	-1.777303056	0.979963743	-0.423067966	53914	55.43	13	113399610	113453524	protein_coding	ADP-ribosylhydrolase like 1	55.43	ADPRHL1
ENSG00000154133	358.274183	575.8748434	-1.341155704	0.961187618	-0.684691213	14809	52.98	11	124883691	124898500	protein_coding	roundabout guidance receptor 4	52.98	ROBO4
ENSG00000154358	5359.774249	9852.824688	-2.872330282	0.999999814	-0.878365151	170744	53.87	1	228208130	228378874	protein_coding	obscurin, cytoskeletal calmodulin and titin-interacting RhoGEF	53.87	OBSCN
ENSG00000156453	358.469786	600.4956671	-1.385435291	0.965703327	-0.744302311	26135	54.92	5	141853111	141879246	protein_coding	protocadherin 1	54.92	PCDH1
ENSG00000156860	206.0814088	363.4633702	-1.297148776	0.955337939	-0.818595615	12383	59.46	16	30658431	30670814	protein_coding	fibrosin	59.46	FBRS
ENSG00000156885	1216.126925	2260.902805	-1.933505597	0.988308513	-0.894605164	697	71.49	16	31427731	31428428	protein_coding	cytochrome c oxidase subunit 6A2	71.49	COX6A2
ENSG00000157510	1355.817522	1775.757874	-1.256820184	0.951364547	-0.389271861	69931	46.32	5	149271871	149341802	protein_coding	actin filament associated protein 1 like 1	46.32	AFAP1L1
ENSG00000157637	353.0246624	738.5149089	-2.062179457	0.988799389	-1.064858072	50547	56.73	17	81245000	81295547	protein_coding	solute carrier family 38 member 10	56.73	SLC38A10
ENSG00000158863	641.0279661	951.1080035	-1.49009025	0.976075658	-0.569221878	15739	57.17	8	22089159	22104898	protein_coding	family with sequence similarity 160 member B2	57.17	FAM160B2
ENSG00000159069	810.8852269	1828.534912	-3.156376706	0.9962667	-1.173118538	4261	68.96	9	136940435	136944696	protein_coding	F-box and WD repeat domain containing 5	68.96	FBXW5
ENSG00000159640	110.4298753	263.0390584	-1.555327319	0.9770621	-1.252146513	21319	58.35	17	63477061	63498380	protein_coding	angiotensin I converting enzyme	58.35	ACE
ENSG00000159840	431.6317896	807.5189534	-1.712539138	0.978883301	-0.903694999	10031	58.32	7	143381080	143391111	protein_coding	zyxin	58.32	ZYX
ENSG00000159842	2896.094747	3801.990328	-1.725000701	0.978839248	-0.39264606	225503	55.2	17	1003518	1229021	protein_coding	active BCR-related	55.2	ABR
ENSG00000160014	2934.304763	3591.426811	-1.226347859	0.950274866	-0.291538395	9719	57.51	19	46601074	46610793	protein_coding	calmodulin 3	57.51	CALM3
ENSG00000160271	757.4253769	1072.800835	-1.255577649	0.951291063	-0.502206602	51614	58.92	9	133097720	133149334	protein_coding	ral guanine nucleotide dissociation stimulator	58.92	RALGDS
ENSG00000160285	819.801889	1132.597582	-1.311091387	0.956961793	-0.466288135	40683	53.33	21	46188141	46228824	protein_coding	lanosterol synthase	53.33	LSS
ENSG00000160360	260.7620687	479.0646733	-1.314509081	0.957389305	-0.877486406	32129	66.05	9	136327476	136359605	protein_coding	G protein signaling modulator 1	66.05	GPSM1
ENSG00000160392	668.9992458	1032.960384	-1.574158393	0.978237699	-0.626708435	28991	50.5	19	40319536	40348527	protein_coding	chromosome 19 open reading frame 47	50.5	C19orf47
ENSG00000160445	1319.002174	1906.675893	-1.715283176	0.978865639	-0.531612686	42628	51.42	9	128729786	128772414	protein_coding	zyg-11 related cell cycle regulator	51.42	ZER1
ENSG00000160446	20.97348038	59.76644908	-1.488749234	0.976065171	-1.510769545	3258	61.4	9	128720869	128724127	protein_coding	zinc finger DHHC-type containing 12	61.4	ZDHHC12
ENSG00000160539	1333.949812	2027.716705	-1.594912003	0.979594854	-0.604151717	19568	54.56	9	131289694	131309262	protein_coding	phospholipid phosphatase 7 (inactive)	54.56	PLPP7
ENSG00000160685	361.2581565	587.1443411	-1.492165496	0.976088036	-0.700685052	15892	60.41	1	155002630	155018522	protein_coding	zinc finger and BTB domain containing 7B	60.41	ZBTB7B
ENSG00000160703	632.9377754	949.3492201	-1.51134725	0.976092491	-0.584875209	17448	53.41	11	119166568	119184016	protein_coding	NLR family member X1	53.41	NLRX1
ENSG00000160789	1433.043763	2603.179455	-3.153919811	0.99585053	-0.861192102	57516	53.04	1	156082573	156140089	protein_coding	lamin A/C	53.04	LMNA
ENSG00000160877	234.4681012	406.7751426	-1.392521699	0.966285309	-0.79483986	23038	55.78	19	13118103	13141141	protein_coding	nucleus accumbens associated 1	55.78	NACC1
ENSG00000160948	819.1876687	1219.735476	-1.501311301	0.976103708	-0.574302401	4962	63.23	8	144423601	144428563	protein_coding	VPS28, ESCRT-I subunit	63.23	VPS28
ENSG00000160972	115.543541	374.9758128	-2.070665803	0.98833882	-1.698360926	24152	57.4	8	144477969	144502121	protein_coding	protein phosphatase 1 regulatory subunit 16A	57.4	PPP1R16A
ENSG00000161011	2618.192792	3630.862977	-1.71898619	0.97885102	-0.471741151	31680	54.99	5	179806398	179838078	protein_coding	sequestosome 1	54.99	SQSTM1
ENSG00000161677	36.93687978	94.3537664	-1.353200267	0.962833271	-1.353018108	5355	61.76	19	50505998	50511353	protein_coding	Josephin domain containing 2	61.76	JOSD2
ENSG00000161714	1017.005898	1515.005244	-1.547318285	0.976680414	-0.574994741	24387	57.8	17	45108967	45133354	protein_coding	phospholipase C delta 3	57.8	PLCD3
ENSG00000162244	1591.502084	2362.286824	-1.528965257	0.976182802	-0.569795098	2342	53.82	3	51993600	51995942	protein_coding	ribosomal protein L29	53.82	RPL29
ENSG00000162337	485.4770662	975.8241291	-2.19952168	0.985268962	-1.007218012	136666	54.62	11	68312609	68449275	protein_coding	LDL receptor related protein 5	54.62	LRP5
ENSG00000162390	545.2032009	884.5059685	-1.256493941	0.951344579	-0.698077846	96935	51.2	1	54542257	54639192	protein_coding	acyl-CoA thioesterase 11	51.2	ACOT11
ENSG00000162413	1786.496452	2752.415373	-1.631506296	0.981362667	-0.623565158	23883	54.46	1	6590724	6614607	protein_coding	kelch like family member 21	54.46	KLHL21
ENSG00000162458	1130.074649	1720.01187	-1.76568294	0.979147946	-0.606000446	29987	53.53	1	15756607	15786594	protein_coding	filamin binding LIM protein 1	53.53	FBLIM1
ENSG00000162585	87.96418278	175.4694471	-1.222328395	0.950064483	-0.996231737	28259	60.96	1	2184461	2212720	protein_coding	Fanconi anemia core complex associated protein 20	60.96	FAAP20
ENSG00000162729	220.8871816	398.3912375	-1.349719313	0.962387084	-0.850876217	7603	58.9	1	160091340	160098943	protein_coding	immunoglobulin superfamily member 8	58.9	IGSF8
ENSG00000162910	182.2643715	324.9232278	-1.296251487	0.955237054	-0.834066306	2633	56.68	1	228106679	228109312	protein_coding	mitochondrial ribosomal protein L55	56.68	MRPL55
ENSG00000163050	4279.482017	6093.570261	-1.803711227	0.983244909	-0.509851575	90009	49.11	1	226897536	226987545	protein_coding	coenzyme Q8A	49.11	COQ8A
ENSG00000163956	2223.414523	3289.049021	-2.377544105	0.989944972	-0.564893568	26183	57.65	4	3506376	3532559	protein_coding	LDL receptor related protein associated protein 1	57.65	LRPAP1
ENSG00000164054	481.9220568	744.2448162	-1.250763872	0.951048012	-0.626977435	37028	50.15	3	48467798	48504826	protein_coding	shisa family member 5	50.15	SHISA5
ENSG00000164880	330.2364576	711.7222979	-2.084701231	0.987736851	-1.107815035	34090	63.54	7	1470277	1504367	protein_coding	integrator complex subunit 1	63.54	INTS1
ENSG00000164889	265.7309488	510.0609192	-1.571319055	0.978046479	-0.940703302	19317	59.83	7	151057210	151076527	protein_coding	solute carrier family 4 member 2	59.83	SLC4A2
ENSG00000164896	883.8865315	1419.286656	-1.861665673	0.985921789	-0.683232921	4273	61.75	7	151076593	151080866	protein_coding	Fas activated serine/threonine kinase	61.75	FASTK
ENSG00000164897	60.20659316	157.3159803	-1.791144112	0.981566548	-1.38567184	2466	67.61	7	151081080	151083546	protein_coding	transmembrane and ubiquitin like domain containing 1	67.61	TMUB1
ENSG00000165175	265.4938584	459.1306737	-1.242242851	0.950753257	-0.790226331	5105	53.8	X	38801432	38806537	protein_coding	MID1 interacting protein 1	53.8	MID1IP1
ENSG00000165238	1234.035758	2410.049494	-2.563698995	0.998699317	-0.965678575	135656	52.37	9	93184916	93320572	protein_coding	WNK lysine deficient protein kinase 2	52.37	WNK2
ENSG00000165458	1838.500349	2446.465494	-1.662241941	0.981075661	-0.412169484	15404	58.64	11	72223701	72239105	protein_coding	inositol polyphosphate phosphatase like 1	58.64	INPPL1
ENSG00000165644	47.25323952	107.3746122	-1.250495514	0.95103605	-1.184167777	2061	70.61	10	75233969	75236030	protein_coding	catechol-O-methyltransferase domain containing 1	70.61	COMTD1
ENSG00000165672	10926.62299	8578.513278	2.05460899	0.955886199	0.349048042	11130	46.03	10	119167703	119178833	protein_coding	peroxiredoxin 3	46.03	PRDX3
ENSG00000165804	87.4785239	252.2248956	-1.864518946	0.98600941	-1.5277099	14676	55.48	14	21090046	21104722	protein_coding	zinc finger protein 219	55.48	ZNF219
ENSG00000165886	128.3451181	319.9685544	-1.900253015	0.988344356	-1.317901708	72341	49.52	10	97498868	97571209	protein_coding	ubiquitin domain containing 1	49.52	UBTD1
ENSG00000165912	2140.536722	3016.113907	-1.483858105	0.976007656	-0.494718329	8918	59.66	11	47177525	47186443	protein_coding	protein kinase C and casein kinase substrate in neurons 3	59.66	PACSIN3
ENSG00000165915	466.1852068	846.7195654	-1.794301158	0.981989138	-0.860981001	9369	60.6	11	47407132	47416501	protein_coding	solute carrier family 39 member 13	60.6	SLC39A13
ENSG00000165917	51.89366579	118.2376101	-1.232651303	0.950502654	-1.188058657	11421	53.41	11	47437757	47449178	protein_coding	receptor associated protein of the synapse	53.41	RAPSN
ENSG00000165949	1106.074523	1645.810659	-1.555506547	0.97707163	-0.57334978	11862	48.7	14	94104836	94116698	protein_coding	interferon alpha inducible protein 27	48.7	IFI27
ENSG00000166165	2074.521608	2833.367099	-1.317719744	0.957805825	-0.449738849	3452	70.4	14	103519659	103523111	protein_coding	creatine kinase B	70.4	CKB
ENSG00000166289	144.3399888	376.3126484	-1.903328998	0.988514949	-1.382460733	10401	56.89	19	29665056	29675457	protein_coding	pleckstrin homology and FYVE domain containing 1	56.89	PLEKHF1
ENSG00000166295	6114.461901	4983.912719	1.988003514	0.954200185	0.294946738	19860	43.46	10	72216000	72235860	protein_coding	anaphase promoting complex subunit 16	43.46	ANAPC16
ENSG00000166816	996.4490241	1602.867559	-1.848612704	0.985738119	-0.685787316	4911	62.44	16	75111860	75116771	protein_coding	lactate dehydrogenase D	62.44	LDHD
ENSG00000166925	414.9090574	785.8091258	-1.70036039	0.979085816	-0.921383771	15920	58.03	7	100463359	100479279	protein_coding	TSC22 domain family member 4	58.03	TSC22D4
ENSG00000167173	136.9130531	293.2224001	-1.423217346	0.97024778	-1.098735322	16526	58.35	15	75195643	75212169	protein_coding	chromosome 15 open reading frame 39	58.35	C15orf39
ENSG00000167244	922.4309035	1781.519725	-1.960537994	0.986300849	-0.949595705	12126	63.23	11	2129112	2141238	protein_coding	insulin like growth factor 2	63.23	IGF2
ENSG00000167393	887.0487563	1599.200161	-2.087206315	0.987701289	-0.850265214	52992	58.29	X	333963	386955	protein_coding	protein phosphatase 2 regulatory subunit B''beta	58.29	PPP2R3B
ENSG00000167522	805.4776611	1154.774709	-1.394847846	0.966499393	-0.519694933	222934	51.19	16	89267627	89490561	protein_coding	ankyrin repeat domain 11	51.19	ANKRD11

ENSG00000167543	145.3403712	327.9437847	-1.654827514	0.981375085	-1.174013037	7105	63.48	17	29566052	29573157	protein_coding	tumor protein p53 inducible protein 13	63.48	TP53I13
ENSG00000167549	1145.629935	1574.54121	-1.501609617	0.976013545	-0.458790421	8151	60.29	17	29614756	29622907	protein_coding	coronin 6	60.29	CORO6
ENSG00000167566	243.4305111	502.0751165	-1.725388728	0.978838632	-1.044393221	37604	52.89	12	49791146	49828750	protein_coding	NCK associated protein 5 like	52.89	NCKAP5L
ENSG00000167658	20633.6661	30816.3065	-2.752080906	0.98980996	-0.578693782	9413	59.53	19	3976056	3985469	protein_coding	eukaryotic translation elongation factor 2	59.53	EEF2
ENSG00000167671	901.6958779	1372.572456	-1.641267274	0.981540933	-0.606169478	12823	59.86	19	4444999	4457822	protein_coding	UBX domain protein 6	59.86	UBXN6
ENSG00000167674	217.8704374	355.8882279	-1.296802168	0.955298928	-0.70795376	29924	57.07	19	4472287	4502211	protein_coding	HDGF like 2	57.07	HDGFL2
ENSG00000167676	2871.091347	4231.876122	-1.705357301	0.978973212	-0.559698164	16285	59.39	19	4502180	4518465	protein_coding	perilipin 4	59.39	PLIN4
ENSG00000167680	52.92153793	176.2130945	-2.042618206	0.98906908	-1.735394243	17215	57.84	19	4542593	4559808	protein_coding	semaphorin 6B	57.84	SEMA6B
ENSG00000167700	82.0181865	172.6456775	-1.406300427	0.967769719	-1.073798464	2139	66.96	8	144509074	144511213	protein_coding	major facilitator superfamily domain containing 3	66.96	MFSD3
ENSG00000167701	195.1133828	397.4715662	-1.558540078	0.977240127	-1.026538923	4201	68.44	8	144502973	144507174	protein_coding	glutamic--pyruvic transaminase	68.44	GPT
ENSG00000167702	18.3617938	43.26641347	-1.271219374	0.952524431	-1.236540529	8159	63.6	8	144466043	144474202	protein_coding	kinesin family member C2	63.6	KIFC2
ENSG00000167705	575.5958607	853.2999874	-1.314390357	0.957374214	-0.567996809	3932	64.3	17	1646145	1650077	protein_coding	Rab interacting lysosomal protein	64.3	RILP
ENSG00000167792	6357.084187	9550.262342	-2.7704563	0.995503104	-0.58717517	5683	55.96	11	67606852	67612535	protein_coding	NADH:ubiquinone oxidoreductase core subunit V1	55.96	NDUFV1
ENSG00000167874	163.3163248	339.4462921	-1.430165961	0.971349017	-1.055514319	1034	63.09	17	7855065	7856099	protein_coding	transmembrane protein 88	63.09	TMEM88
ENSG00000167900	8.364988374	27.00827941	-1.357819604	0.96337647	-1.690966294	13158	53.2	17	78174075	78187233	protein_coding	thymidine kinase 1	53.2	TK1
ENSG00000167930	633.9357253	960.3293545	-1.444707163	0.973448738	-0.599192704	34425	52.35	16	234546	268971	protein_coding	family with sequence similarity 234 member A	52.35	FAM234A
ENSG00000167965	223.5715789	381.8366013	-1.267276887	0.952155981	-0.7722186	5168	61.98	16	2204248	2209416	protein_coding	MTOR associated protein, LST8 homolog	61.98	MLST8
ENSG00000167967	64.51246225	164.1632766	-1.727024645	0.978835951	-1.347481645	12176	61.58	16	2223566	2235742	protein_coding	E4F transcription factor 1	61.58	E4F1
ENSG00000167969	872.2994422	1609.747109	-2.015164305	0.987119199	-0.883938687	12905	55.22	16	2239395	2252300	protein_coding	enoyl-CoA delta isomerase 1	55.22	ECI1
ENSG00000167996	174.2079837	319.4698969	-1.223662082	0.950141254	-0.874869253	7942	50.42	11	61959718	61967660	protein_coding	ferritin heavy chain 1	50.42	FTH1
ENSG00000168056	674.1136716	1134.316536	-1.860870497	0.985901325	-0.750759497	20125	60.22	11	65538805	65558930	protein_coding	latent transforming growth factor beta binding protein 3	60.22	LTBP3
ENSG00000168159	1223.594045	1763.56851	-1.519960062	0.976105178	-0.527372632	8705	56.59	1	228487061	228495766	protein_coding	ring finger protein 187	56.59	RNF187
ENSG00000168397	416.1558801	612.4833886	-1.22662381	0.950287553	-0.557546695	36644	52.33	2	241637213	241673857	protein_coding	autophagy related 4B cysteine peptidase	52.33	ATG4B
ENSG00000168477	898.6855452	1444.244139	-1.503930744	0.976101201	-0.684426338	74180	54.31	6	32041154	32115334	protein_coding	tenascin XB	54.31	TNXB
ENSG00000168542	3278.787926	5789.433278	-1.228461259	0.950363004	-0.820259541	38426	33.8	2	188974320	189012746	protein_coding	collagen type III alpha 1 chain	33.8	COL3A1
ENSG00000168936	729.1262922	1120.082649	-1.536418311	0.976322687	-0.619364559	5406	62.46	4	1715952	1721358	protein_coding	transmembrane protein 129	62.46	TMEM129
ENSG00000169504	14250.30768	10127.80358	1.968687105	0.956852401	0.492671738	98967	41.67	1	24745357	24844324	protein_coding	chloride intracellular channel 4	41.67	CLIC4
ENSG00000169564	288.327038	548.1310471	-1.527134292	0.976159838	-0.926814721	1749	55.2	2	70087454	70089203	protein_coding	poly(rC) binding protein 1	55.2	PCBP1
ENSG00000169692	245.9304033	587.0026034	-2.100744987	0.9880122	-1.255116802	14280	62.68	9	136673143	136687423	protein_coding	1-acylglycerol-3-phosphate O-acyltransferase 2	62.68	AGPAT2
ENSG00000169696	111.23963	225.7644412	-1.3611657	0.963733416	-1.021147422	40599	60.76	17	81976807	82017406	protein_coding	ASPSCR1, UBX domain containing tether for SLC2A4	60.76	ASPSCR1
ENSG00000169718	317.8032794	549.5989483	-1.612510309	0.980575035	-0.790245232	8381	64.45	17	82057506	82065887	protein_coding	dihydrouridine synthase 1 like	64.45	DUS1L
ENSG00000169727	663.9737357	1084.59459	-1.641736631	0.981543228	-0.7079578	6779	66.61	17	82050691	82057470	protein_coding	G protein pathway suppressor 1	66.61	GPS1
ENSG00000169957	387.2029918	610.0421115	-1.384166396	0.96560727	-0.655818736	2820	64.52	16	30524001	30526821	protein_coding	zinc finger protein 768	64.52	ZNF768
ENSG00000170421	1469.792637	2014.480603	-1.417690381	0.969383521	-0.454795285	52767	50.58	12	52897187	52949954	protein_coding	keratin 8	50.58	KRT8
ENSG00000170604	98.77783415	195.0997333	-1.315491117	0.957515149	-0.981952565	2563	68.49	19	45883607	45886170	protein_coding	interferon regulatory factor 2 binding protein 1	68.49	IRF2BP1
ENSG00000170638	61.65226736	148.1023331	-1.367254061	0.964309145	-1.264368509	13683	62.8	22	50185915	50199598	protein_coding	TraB domain containing	62.8	TRABD
ENSG00000170906	1287.562307	1842.939458	-1.397507023	0.966760714	-0.517366431	6529	54.99	19	54102728	54109257	protein_coding	NADH:ubiquinone oxidoreductase subunit A3	54.99	NDUFA3
ENSG00000171033	9216.224912	7333.564173	1.939659683	0.955543153	0.329661394	89128	34.75	8	78516139	78605267	protein_coding	cAMP-dependent protein kinase inhibitor alpha	34.75	PKIA
ENSG00000171159	111.7241054	261.3457724	-1.728303272	0.978833674	-1.226019329	3668	56.88	9	128160260	128163928	protein_coding	chromosome 9 open reading frame 16	56.88	C9orf16
ENSG00000171206	696.0017269	1005.003461	-1.360650034	0.963680882	-0.530037679	13911	57.48	10	102644496	102658407	protein_coding	tripartite motif containing 8	57.48	TRIM8
ENSG00000171222	47.28410398	161.5548822	-1.813936498	0.984414167	-1.772597187	5855	47.71	20	35953617	35959472	protein_coding	SCAN domain containing 1	47.71	SCAND1
ENSG00000171298	566.9035308	1132.912184	-2.30372125	0.991327914	-0.998860877	18323	58.33	17	80101556	80119879	protein_coding	glucosidase alpha, acid	58.33	GAA
ENSG00000171443	35.51721559	93.28920563	-1.352858847	0.962790354	-1.393191674	3116	62.18	19	55600022	55603138	protein_coding	zinc finger protein 524	62.18	ZNF524
ENSG00000171680	194.5975435	430.2208841	-1.887984489	0.987465201	-1.14458406	53969	58.85	1	6466092	6520061	protein_coding	pleckstrin homology and RhoGEF domain containing G5	58.85	PLEKHG5
ENSG00000171992	15293.82974	18739.29809	-1.535695232	0.976305549	-0.293117198	58140	52.53	5	150601080	150659220	protein_coding	synaptopodin	52.53	SYNPO
ENSG00000172037	6577.948193	10027.91661	-3.605970338	1	-0.608312354	12004	57.34	3	49121114	49133118	protein_coding	laminin subunit beta 2	57.34	LAMB2
ENSG00000172046	1201.400495	1599.263823	-1.328119097	0.959255671	-0.41269079	12892	56.09	3	49108046	49120938	protein_coding	ubiquitin specific peptidase 19	56.09	USP19
ENSG00000172115	23505.17061	16986.92339	2.823406688	0.99967242	0.468553572	5270	43.01	7	25120091	25125361	protein_coding	cytochrome c, somatic	43.01	CYCS
ENSG00000172270	12644.45244	23282.96252	-3.726731793	1	-0.880770074	12216	62.58	19	571277	583493	protein_coding	basigin (Ok blood group)	62.58	BSG
ENSG00000172346	2076.753537	3552.503045	-2.620016778	0.992580251	-0.774505875	16978	55.8	22	41560763	41577741	protein_coding	cold shock domain containing C2	55.8	CSDC2
ENSG00000172354	739.6724413	1304.207037	-1.925347969	0.988701525	-0.818213894	5643	65.91	7	100673531	100679174	protein_coding	G protein subunit beta 2	65.91	GNB2
ENSG00000172366	290.6238219	564.1631462	-1.559177037	0.977276911	-0.956959462	NA	NA	NA	NA	NA	NA	NA	NA	NA
ENSG00000172534	466.6181233	735.5107811	-1.435270164	0.972134575	-0.656504146	24254	59.51	X	153947553	153971807	protein_coding	host cell factor C1	59.51	HCFC1
ENSG00000172638	861.2594803	1327.28596	-1.658905433	0.981226987	-0.623959366	7151	60.57	11	65866441	65873592	protein_coding	EGF containing fibulin like extracellular matrix protein 2	60.57	EFEMP2
ENSG00000172663	281.7442656	481.8923041	-1.277341534	0.953149352	-0.774324514	7562	56.67	11	67461710	67469272	protein_coding	transmembrane protein 134	56.67	TMEM134
ENSG00000172725	112.4073522	220.7235351	-1.311103526	0.956963286	-0.973504068	8311	64.53	11	67435510	67443821	protein_coding	coronin 1B	64.53	CORO1B
ENSG00000172889	284.9691523	826.3768298	-2.392673362	0.987721166	-1.535994047	13822	67.31	9	136658856	136672678	protein_coding	EGF like domain multiple 7	67.31	EGFL7
ENSG00000173020	723.4649086	1064.108854	-1.39238783	0.966273509	-0.556650794	20146	61.02	11	67266410	67286556	protein_coding	G protein-coupled receptor kinase 2	61.02	GRK2
ENSG00000173153	891.7963479	1550.410253	-2.025503697	0.98803819	-0.79786382	11171	57.05	11	64305572	64316743	protein_coding	estrogen related receptor alpha	57.05	ESRRA
ENSG00000173175	4225.741525	6642.815054	-2.488986907	0.994663298	-0.652590223	167462	51.27	3	123282296	123449758	protein_coding	adenylate cyclase 5	51.27	ADCY5
ENSG00000173264	144.4932051	258.7685401	-1.379158944	0.965238571	-0.840660581	19438	56.27	11	64270062	64289500	protein_coding	G protein-coupled receptor 137	56.27	GPR137
ENSG00000173272	190.7628924	493.3565286	-1.727210126	0.978835636	-1.370850038	27843	51.73	2	131464900	131492743	protein_coding	mitotic spindle organizing protein 2A	51.73	MZT2A
ENSG00000173327	241.5405198	450.4045204	-1.400732982	0.967104808	-0.898956077	17627	57.13	11	65597755	65615382	protein_coding	mitogen-activated protein kinase kinase kinase 11	57.13	MAP3K11
ENSG00000173511	1238.95195	1797.943253	-1.266349639	0.952073833	-0.53722725	4255	61.25	11	64234538	64238793	protein_coding	vascular endothelial growth factor B	61.25	VEGFB
ENSG00000173581	125.1280844	229.1769067	-1.223387254	0.950125561	-0.873056044	12099	61.17	19	55641062	55653161	protein_coding	coiled-coil domain containing 106	61.17	CCDC106
ENSG00000173621	23.54420501	58.41427702	-1.350936544	0.962546107	-1.310949011	3828	70.38	11	66856647	66860475	protein_coding	leucine rich repeat and fibronectin type III domain containing 4	70.38	LRFN4
ENSG00000173641	61909.12043	75407.12871	-1.326881679	0.959076012	-0.284548954	5566	61.04	1	16014028	16019594	protein_coding	heat shock protein family B (small) member 7	61.04	HSPB7
ENSG00000173801	2613.288855	4770.124591	-2.903946013	0.999999999	-0.86816035	32327	54.96	17	41754604	41786931	protein_coding	junction plakoglobin	54.96	JUP
ENSG00000173918	629.2404157	984.2711879	-1.428837832	0.971139938	-0.645444527	26974	54.12	17	79022814	79049788	protein_coding	C1q and TNF related 1	54.12	C1QTNF1
ENSG00000173991	28934.14589	52059.23452	-4.429339551	1	-0.847381041	2368	62.39	17	39664187	39666555	protein_coding	titin-cap	62.39	TCAP
ENSG00000174276	16.96411117	45.29765044	-1.371850628	0.964689991	-1.416950379	1305	67.92	11	65116403	65117708	protein_coding	zinc finger HIT-type containing 2	67.92	ZNHIT2
ENSG00000174917	968.6350496	1502.109809	-1.553875001	0.976986043	-0.63296517	2475	61.19	19	5678421	5680896	protein_coding	chromosome 19 open reading frame 70	61.19	C19orf70
ENSG00000175182	325.4459658	520.0068748	-1.369210695	0.964476384	-0.676112666	12495	55.67	3	184335926	184348421	protein_coding	family with sequence similarity 131 member A	55.67	FAM131A
ENSG00000175221	104.356025	250.0774214	-1.530747947	0.976209015	-1.260860911	25588	62.67	19	867630	893218	protein_coding	mediator complex subunit 16	62.67	MED16
ENSG00000175274	439.6440846	692.4884343	-1.286527275	0.954151519	-0.655453918	65403	54.85	11	44885903	44951306	protein_coding	tumor protein p53 inducible protein 11	54.85	TP53I11
ENSG00000175416	1535.628648	2216.943464	-1.819835196	0.98492206	-0.5297426	24114	53.09	5	176392455	176416569	protein_coding	clathrin light chain B	53.09	CLTB
ENSG00000175550	476.9261742	769.8348138	-1.482122966	0.975978402	-0.690782953	2304	65.51	11	65919257	65921561	protein_coding	DR1 associated protein 1	65.51	DRAP1
ENSG00000175602	33.15910909	110.9309304	-1.734311082	0.978818904	-1.742184532	1523	67.65	11	65890112	65891635	protein_coding	coiled-coil domain containing 85B	67.65	CCDC85B
ENSG00000175662	6924.120024	8719.342369	-1.5620641	0.977449146	-0.332588595	128911	48.26	17	17843511	17972422	protein_coding	target of myb1 like 2 membrane trafficking protein	48.26	TOM1L2
ENSG00000175701	277.9180881	698.1997153	-2.419116134	0.984073646	-1.328980034	33891	40.57	2	110211529	110245420	protein_coding	long intergenic non-protein coding RNA 116	40.57	LINC00116
ENSG00000175756	909.7950104	1489.027399	-1.855269388	0.985796022	-0.710756873	1765	66.87	1	1373730	1375495	protein_coding	aurora kinase A interacting protein 1	66.87	AURKAIP1
ENSG00000175899	15982.34945	18924.71642	-1.306895426	0.956456513	-0.243792178	48565	37.18	12	9067664	9116229	protein_coding	alpha-2-macroglobulin	37.18	A2M
ENSG00000175920	207.505823	489.0329707	-1.883472407	0.98711565	-1.236779913	31172	62.3	4	3463311	3494483	protein_coding	docking protein 7	62.3	DOK7
ENSG00000176058	93.42201399	189.5579821	-1.353253002	0.962839685	-1.020804756	12576	62.56	9	137191617	137204193	protein_coding	taperin	62.56	TPRN
ENSG00000176101	328.8784105	624.2014385	-1.641378229	0.981541475	-0.924457377	1723	65.89	9	137188647	137190370	protein_coding	SS nuclear autoantigen 1	65.89	SSNA1
ENSG00000176248	183.446485	373.3157717	-1.511690249	0.976092267	-1.025037199	13765	61.65	9	137174784	137188549	protein_coding	anaphase promoting complex subunit 2	61.65	ANAPC2
ENSG00000176946	786.9070423	1214.145123	-1.505478982	0.976099197	-0.625675748	53044	49.81	2	241584405	241637449	protein_coding	THAP domain containing 4	49.81	THAP4
ENSG00000176978	479.2076192	1119.135195	-2.456251927	0.985895803	-1.223661577	4635	68.7	9	137110542	137115177	protein_coding	dipeptidyl peptidase 7	68.7	DPP7
ENSG00000177045	19.27426585	60.80860164	-1.441078196	0.97296997	-1.657599504	4441	67.67	19	45764785	45769226	protein_coding	SIX homeobox 5	67.67	SIX5
ENSG00000177169	457.5951759	1000.312079	-2.493398437	0.995649975	-1.128306417	28516	62.39	12	131894651	131923167	protein_coding	unc-51 like autophagy activating kinase 1	62.39	ULK1
ENSG00000177191	31.83302661	81.77858586	-1.248297939	0.950947386	-1.361198784	3371	64.26	19	41425359	41428730	protein_coding	UDP-GlcNAc:betaGal beta-1,3-N-acetylglucosaminyltransferase 8	64.26	B3GNT8
ENSG00000177303	267.6150943	522.6611751	-1.591978906	0.979414258	-0.965716505	15322	61.19	17	75500261	75515583	protein_coding	CASK interacting protein 2	61.19	CASKIN2
ENSG00000177548	88.69910699	204.3715828	-1.481879977	0.97597382	-1.204203123	32105	52.63	16	28904421	28936526	protein_coding	rabaptin, RAB GTPase binding effector protein 2	52.63	RABEP2
ENSG00000177600	2411.662479	3502.739542	-1.625036394	0.981143	-0.538455705	3233	57.05	11	809647	812880	protein_coding	ribosomal protein lateral stalk subunit P2	57.05	RPLP2
ENSG00000177666	1416.030875	2252.683071	-2.390700575	0.988016065	-0.669791633	6671	64.55	11	818902	825573	protein_coding	patatin like phospholipase domain containing 2	64.55	PNPLA2
ENSG00000177697	2723.257895	3798.988885	-1.59074895	0.979337161	-0.480281878	6988	63.43	11	832843	839831	protein_coding	CD151 molecule (Raph blood group)	63.43	CD151
ENSG00000177700	694.6784439	1418.088946	-2.231717946	0.981294848	-1.029530789	5189	61.14	11	837356	842545	protein_coding	RNA polymerase II subunit L	61.14	POLR2L
ENSG00000177728	618.130043	940.4502838	-1.438600921	0.972622968	-0.605441293	58931	51.96	17	75441159	75500090	protein_coding	transmembrane protein 94	51.96	TMEM94
ENSG00000177731	2448.277053	3188.146292	-1.456843288	0.974748469	-0.380951005	14080	59.66	17	18244836	18258916	protein_coding	FLII, actin remodeling protein	59.66	FLII
ENSG00000178209	1742.794742	3916.798701	-2.996624894	0.999992883	-1.168272319	61587	63.62	8	143915147	143976734	protein_coding	plectin	63.62	PLEC
ENSG00000178605	300.7971221	670.3530999	-1.904392071	0.988567216	-1.156130453	14290	57.63	X	304529	318819	protein_coding	GTP binding protein 6 (putative)	57.63	GTPBP6

ENSG00000178719	815.3301185	1164.214521	-1.414747954	0.968939705	-0.513900702	3357	66.68	8	143990058	143993415	protein_coding	glutamate ionotropic receptor NMDA type subunit associated protein 1	66.68	GRINA
ENSG00000178814	276.2586923	684.6496262	-2.152610207	0.988879719	-1.30934601	12699	65.95	8	144051266	144063965	protein_coding	5-oxoprolinase, ATP-hydrolysing	65.95	OPLAH
ENSG00000178950	173.0397827	318.4174011	-1.459567047	0.974971813	-0.879815421	83097	56.5	4	849276	932373	protein_coding	cyclin G associated kinase	56.5	GAK
ENSG00000179091	7860.381177	9504.191734	-1.269920848	0.952399597	-0.273964665	2498	63.31	8	144095027	144097525	protein_coding	cytochrome c1	63.31	CYC1
ENSG00000179094	640.4526425	1301.524447	-1.938915869	0.987950373	-1.023038609	16034	58.98	17	8140472	8156506	protein_coding	period circadian clock 1	58.98	PER1
ENSG00000179115	389.793815	585.2526235	-1.247698266	0.950924863	-0.586348298	11558	53.91	19	12922479	12934037	protein_coding	phenylalanyl-tRNA synthetase alpha subunit	53.91	FARSA
ENSG00000179262	1290.696539	1814.192368	-1.727017705	0.978835963	-0.491177598	7787	54.44	19	12945855	12953642	protein_coding	RAD23 homolog A, nucleotide excision repair protein	54.44	RAD23A
ENSG00000179271	452.0139559	726.5655677	-1.262137108	0.951728801	-0.684725681	4117	55.03	19	12953119	12957236	protein_coding	GADD45G interacting protein 1	55.03	GADD45GIP1
ENSG00000179344	64.43250263	282.2152397	-1.926733118	0.988649811	-2.130935359	8916	45.39	6	32659467	32668383	protein_coding	major histocompatibility complex, class II, DQ beta 1	45.39	HLA-DQB1
ENSG00000179364	460.6384465	853.7680041	-1.950301238	0.98707182	-0.890209269	97584	57.4	14	105300563	105398147	protein_coding	phosphofurin acidic cluster sorting protein 2	57.4	PACS2
ENSG00000179526	302.0040626	542.3693707	-1.356821429	0.963263391	-0.84470775	9491	60.75	8	144098633	144108124	protein_coding	SHANK associated RH domain interactor	60.75	SHARPIN
ENSG00000179611	271.4199624	442.236678	-1.226076982	0.950262426	-0.704291852	2775	62.82	13	43968424	43971199	processed_pseudogene		62.82	DGKZP1
ENSG00000179632	2149.285207	2647.366474	-1.304715218	0.956200837	-0.300700981	3112	63.19	8	144104499	144107611	protein_coding	MAF1 homolog, negative regulator of RNA polymerase III	63.19	MAF1
ENSG00000179776	1899.414251	2611.034608	-1.532817264	0.97624528	-0.459066997	38164	49.55	16	66366622	66404786	protein_coding	cadherin 5	49.55	CDH5
ENSG00000179832	166.4988837	338.7311296	-1.494844855	0.976098285	-1.024628072	113924	53.17	8	144148016	144261940	protein_coding	maestro heat like repeat family member 1	53.17	MROH1
ENSG00000179950	1161.790047	1566.839268	-1.483952344	0.976009145	-0.431507814	13515	62.04	8	143816344	143829859	protein_coding	poly(U) binding splicing factor 60	62.04	PUF60
ENSG00000180155	2426.942548	3398.090613	-2.01047206	0.986709053	-0.485584367	6613	64.18	8	142771197	142777810	protein_coding	Ly6/neurotoxin 1	64.18	LYNX1
ENSG00000180509	27.34813294	93.65000751	-1.481693841	0.975970313	-1.775836767	NA	NA	NA	NA	NA	NA	NA	NA	NA
ENSG00000180891	670.9990859	995.1167692	-1.515142331	0.976093023	-0.568555023	94080	51.38	17	57861243	57955323	protein_coding	CUE domain containing 1	51.38	CUEDC1
ENSG00000180900	139.9911784	352.1148332	-1.940039722	0.987869078	-1.330710086	24459	62.5	8	143790920	143815379	protein_coding	scribbled planar cell polarity protein	62.5	SCRIB
ENSG00000181061	3739.255269	2710.068659	1.99086271	0.953629961	0.464421561	20233	42.66	3	42784298	42804531	protein_coding	HIG1 hypoxia inducible domain family member 1A	42.66	HIGD1A
ENSG00000181409	22.88280247	78.24941146	-1.683848439	0.979800209	-1.77381615	48782	61.42	17	81117295	81166077	protein_coding	apoptosis associated tyrosine kinase	61.42	AATK
ENSG00000182054	13202.64271	18178.41829	-2.822591245	0.999910317	-0.461399939	19459	52.54	15	90083045	90102504	protein_coding	isocitrate dehydrogenase (NADP(+)) 2, mitochondrial	52.54	IDH2
ENSG00000182087	500.6581947	863.5941619	-1.635953317	0.98147023	-0.786527496	11531	65.7	19	1009648	1021179	protein_coding	transmembrane protein 259	65.7	TMEM259
ENSG00000182095	207.9560435	416.430907	-1.648991461	0.981509682	-1.001798536	118624	54.68	7	5306790	5425414	protein_coding	trinucleotide repeat containing 18	54.68	TNRC18
ENSG00000182154	703.8840925	1401.232917	-2.268054688	0.984175912	-0.993286998	1356	72.44	9	137551199	137552555	protein_coding	mitochondrial ribosomal protein L41	72.44	MRPL41
ENSG00000182175	167.0263881	325.5952978	-1.256095817	0.951320738	-0.963003816	53931	52.94	15	93035273	93089204	protein_coding	repulsive guidance molecule family member a	52.94	RGMA
ENSG00000182809	2771.386384	6933.471278	-4.285983353	1	-1.322969962	7208	69.57	14	105472962	105480170	protein_coding	cysteine rich protein 2	69.57	CRIP2
ENSG00000182871	224.0994788	720.0890928	-2.356920186	0.992578969	-1.684036121	108583	59.66	21	45405137	45513720	protein_coding	collagen type XVIII alpha 1 chain	59.66	COL18A1
ENSG00000182979	300.3870663	532.7486632	-1.718578157	0.978852324	-0.826632372	50909	59.42	14	105419820	105470729	protein_coding	metastasis associated 1	59.42	MTA1
ENSG00000183072	638.2517473	1087.119252	-1.725396271	0.97883862	-0.768312718	3248	61.5	5	173232109	173235357	protein_coding	NK2 homeobox 5	61.5	NKX2-5
ENSG00000183087	1255.232057	1755.306137	-1.355225275	0.963077756	-0.483768566	43518	57.59	13	113820549	113864067	protein_coding	growth arrest specific 6	57.59	GAS6
ENSG00000183207	496.2257296	782.2893759	-1.519104344	0.976101705	-0.656705831	22547	53.06	19	48993448	49015995	protein_coding	RuvB like AAA ATPase 2	53.06	RUVBL2
ENSG00000183458	6.207412245	28.14325882	-1.666578386	0.980847002	-2.180725527	24157	62.84	16	14911551	14935708	transcribed_unprocessed_pseudogene		62.84	
ENSG00000183751	138.8819086	270.4493896	-1.389940076	0.966062342	-0.961499962	10896	64.29	16	1972037	1982933	protein_coding	transducin beta like 3	64.29	TBL3
ENSG00000183963	1516.983118	2560.478739	-2.151764364	0.9889393	-0.75520855	40652	54.79	22	31064105	31104757	protein_coding	smoothelin	54.79	SMTN
ENSG00000184113	199.7435498	520.2354454	-1.656501822	0.981319137	-1.381015782	4521	63.73	22	19523024	19527545	protein_coding	claudin 5	63.73	CLDN5
ENSG00000184281	129.0476417	281.7864064	-1.513631162	0.976092034	-1.126698239	3390	62.64	11	2400488	2403878	protein_coding	tumor suppressing subtransferable candidate 4	62.64	TSSC4
ENSG00000184343	416.9722673	709.7651629	-1.530359436	0.976203101	-0.767390331	9320	64.63	X	153776412	153785732	protein_coding	SRSF protein kinase 3	64.63	SRPK3
ENSG00000184489	3124.213401	7110.672468	-3.352584084	1	-1.186493004	40461	62.4	8	141391993	141432454	protein_coding	protein tyrosine phosphatase type IVA, member 3	62.4	PTP4A3
ENSG00000184601	461.7621486	998.5146353	-2.065719528	0.988616381	-1.112633656	10831	62.78	14	104579684	104590515	protein_coding	chromosome 14 open reading frame 180	62.78	C14orf180
ENSG00000184640	1067.707654	1526.150525	-1.493493886	0.976093846	-0.515380583	220027	54.75	17	77280569	77500596	protein_coding	septin 9	54.75	SEPT9
ENSG00000184682	20.34585126	52.84263814	-1.349939735	0.962416375	-1.37696785	NA	NA	NA	NA	NA	NA	NA	NA	NA
ENSG00000184702	1517.405229	2055.591944	-1.304626709	0.956190563	-0.437947489	10308	66.81	22	19714464	19724772	protein_coding	septin 5	66.81	SEPT5
ENSG00000184916	123.6010668	286.0558325	-1.559749618	0.97731044	-1.210605565	27843	65.88	14	105140981	105168824	protein_coding	jagged 2	65.88	JAG2
ENSG00000184967	39.03190146	103.6610217	-1.525236713	0.976140083	-1.409147868	8025	63.79	12	132144448	132152473	protein_coding	nucleolar complex associated 4 homolog	63.79	NOC4L
ENSG00000184990	195.5490205	422.4347533	-1.453709378	0.974460313	-1.111198219	15394	52.32	14	104753100	104768494	protein_coding	SIVA1 apoptosis inducing factor	52.32	SIVA1
ENSG00000185000	304.8600512	538.7330259	-1.637884987	0.981503889	-0.821423397	12326	63.02	8	144314584	144326910	protein_coding	diacylglycerol O-acyltransferase 1	63.02	DGAT1
ENSG00000185024	141.6263055	262.9073293	-1.23218366	0.950488485	-0.892465107	106303	56.66	14	105209286	105315589	protein_coding	BRF1, RNA polymerase III transcription initiation factor subunit	56.66	BRF1
ENSG00000185049	62.59271581	125.6082344	-1.238413601	0.950653936	-1.004864366	59189	48.3	4	1982714	2041903	protein_coding	negative elongation factor complex member A	48.3	NELFA
ENSG00000185122	1100.992496	2109.276662	-2.564694176	0.998627618	-0.937943702	23131	56.87	8	144291591	144314722	protein_coding	heat shock transcription factor 1	56.87	HSF1
ENSG00000185187	104.3157831	202.6875693	-1.350636923	0.962507463	-0.958300156	11739	65.22	11	405716	417455	protein_coding	single Ig and TIR domain containing	65.22	SIGIRR
ENSG00000185236	511.4889068	1116.97047	-2.439745981	0.98347014	-1.126816189	14453	59.05	19	8389981	8404434	protein_coding	RAB11B, member RAS oncogene family	59.05	RAB11B
ENSG00000185340	74.5596064	193.1016195	-1.533367148	0.976255891	-1.37289413	6203	64.97	22	29306582	29312785	protein_coding	growth arrest specific 2 like 1	64.97	GAS2L1
ENSG00000185359	242.4331209	422.5270848	-1.469051879	0.975562256	-0.801457012	19812	60.6	17	81683326	81703138	protein_coding	hepatocyte growth factor-regulated tyrosine kinase substrate	60.6	HGS
ENSG00000185504	108.5072298	258.6725099	-1.755667704	0.978847147	-1.25333557	14076	61.6	17	81539885	81553961	protein_coding	Fanconi anemia core complex associated protein 100	61.6	FAAP100
ENSG00000185507	22.66412926	63.48013876	-1.327649999	0.959187468	-1.485894547	3446	67.07	11	612553	615999	protein_coding	interferon regulatory factor 7	67.07	IRF7
ENSG00000185633	368.7917895	724.4514531	-1.793687776	0.98190642	-0.974082478	5812	62.05	12	57234903	57240715	protein_coding	NDUFA4, mitochondrial complex associated like 2	62.05	NDUFA4L2
ENSG00000185787	5032.696658	3945.53379	2.289371678	0.97618485	0.351111148	87646	45.4	15	78810487	78898133	protein_coding	mortality factor 4 like 1	45.4	MORF4L1
ENSG00000186111	366.2522982	630.723312	-1.618643547	0.980870997	-0.784169445	70296	60.55	19	3630183	3700479	protein_coding	phosphatidylinositol-4-phosphate 5-kinase type 1 gamma	60.55	PIP5K1C
ENSG00000186174	191.9110849	358.0291644	-1.424899072	0.970514897	-0.899639067	31733	59.25	11	118893875	118925608	protein_coding	B-cell CLL/lymphoma 9 like	59.25	BCL9L
ENSG00000186350	836.5342533	1314.015833	-1.577483768	0.978462905	-0.651486139	123487	62.16	9	134317098	134440585	protein_coding	retinoid X receptor alpha	62.16	RXRA
ENSG00000186635	617.6064764	950.2771177	-1.37972778	0.965280131	-0.621660409	108530	54.91	11	72685069	72793599	protein_coding	ArfGAP with RhoGAP domain, ankyrin repeat and PH domain 1	54.91	ARAP1
ENSG00000186827	8.792749341	33.05064245	-1.445407594	0.973537047	-1.910292069	2812	70.39	1	1211326	1214138	protein_coding	TNF receptor superfamily member 4	70.39	TNFRSF4
ENSG00000186940	24.70385682	83.01155541	-1.223063744	0.95010711	-1.748575881	455	62.06	9	79391304	79391759	processed_pseudogene		62.06	CHCHD2P9
ENSG00000186994	62.37641191	168.2321654	-1.483268689	0.975997988	-1.431381098	20678	55.19	19	8322584	8343262	protein_coding	KN motif and ankyrin repeat domains 3	55.19	KANK3
ENSG00000187244	690.4681952	1491.767595	-2.478235963	0.991850461	-1.111375927	12361	58.93	19	44809059	44821420	protein_coding	basal cell adhesion molecule (Lutheran blood group)	58.93	BCAM
ENSG00000187498	3368.966183	5085.630592	-1.582049178	0.978770503	-0.594120725	158186	44.9	13	110148963	110307149	protein_coding	collagen type IV alpha 1 chain	44.9	COL4A1
ENSG00000187608	39.84688704	126.3282937	-1.673507428	0.980431946	-1.664638869	13403	56.43	1	1001138	1014541	protein_coding	ISG15 ubiquitin-like modifier	56.43	ISG15
ENSG00000187642	1152.197393	1949.876767	-1.933980823	0.988279273	-0.758995049	6889	67.76	1	975204	982093	protein_coding	PPARGC1 and ESRR induced regulator, muscle 1	67.76	PERM1
ENSG00000187954	312.0003279	558.216003	-1.641888347	0.981543971	-0.839275939	16095	61.94	8	144449582	144465677	protein_coding	cysteine and histidine rich 1	61.94	CYHR1
ENSG00000188026	631.3326442	917.1956531	-1.2683526	0.952253199	-0.538829167	63664	50.55	12	123470054	123533718	protein_coding	Rab interacting lysosomal protein like 1	50.55	RILPL1
ENSG00000188157	140.6622935	344.8871123	-1.550335684	0.976813002	-1.293888574	35995	65.71	1	1020123	1056118	protein_coding	agrin	65.71	AGRN
ENSG00000188290	32.14778999	71.57390661	-1.226927617	0.950301094	-1.154714164	1210	74.32	1	998962	1000172	protein_coding	hes family bHLH transcription factor 4	74.32	HES4
ENSG00000188807	205.707275	364.3002748	-1.267854787	0.952207625	-0.824535266	25951	59.34	1	9588922	9614873	protein_coding	transmembrane protein 201	59.34	TMEM201
ENSG00000188986	464.7682267	698.8736496	-1.307235068	0.956496675	-0.588520208	18373	56.65	9	137255173	137273546	protein_coding	negative elongation factor complex member B	56.65	NELFB
ENSG00000189319	590.3233466	906.8089259	-1.252558939	0.95113122	-0.619293192	124977	52.68	10	124619292	124744269	protein_coding	family with sequence similarity 53 member B	52.68	FAM53B
ENSG00000196365	1163.754017	1926.573912	-2.251175732	0.981554291	-0.727251388	28738	57.82	19	5691834	5720572	protein_coding	lon peptidase 1, mitochondrial	57.82	LONP1
ENSG00000196411	369.0445712	615.4377129	-1.336788377	0.960541528	-0.737817784	24956	54.77	7	100802565	100827521	protein_coding	EPH receptor B4	54.77	EPHB4
ENSG00000196498	493.7087716	958.9726747	-1.944723111	0.987513402	-0.95782943	243174	57.37	12	124324415	124567589	protein_coding	nuclear receptor corepressor 2	57.37	NCOR2
ENSG00000196535	2054.874484	2680.141921	-1.256493339	0.951344542	-0.383259123	109288	53.74	17	29071124	29180412	protein_coding	myosin XVIIIA	53.74	MYO18A
ENSG00000196576	721.2483338	1314.103043	-1.997864028	0.985818702	-0.86551042	32648	64.97	22	50274979	50307627	protein_coding	plexin B2	64.97	PLXNB2
ENSG00000196642	79.59868325	235.9756986	-1.692534332	0.979360435	-1.567821824	33244	57.06	9	136807943	136841187	protein_coding	RAB, member RAS oncogene family like 6	57.06	RABL6
ENSG00000196700	226.6263252	387.4844222	-1.25976135	0.951556365	-0.773822855	13163	63.43	20	63956702	63969865	protein_coding	zinc finger protein 512B	63.43	ZNF512B
ENSG00000196924	2578.163032	3681.675435	-1.260784013	0.951628547	-0.514018952	26114	62.28	X	154348524	154374638	protein_coding	filamin A	62.28	FLNA
ENSG00000196961	496.3050023	860.7872265	-1.838303243	0.985662681	-0.794429674	40145	54.57	19	49766968	49807113	protein_coding	adaptor related protein complex 2 alpha 1 subunit	54.57	AP2A1
ENSG00000197136	142.2439511	265.4209892	-1.292613992	0.954829723	-0.899915158	21666	60.5	11	65615773	65637439	protein_coding	pecanex homolog 3	60.5	PCNX3
ENSG00000197226	850.9802995	1175.178508	-1.283356317	0.953800836	-0.465682278	45793	54.22	5	179862066	179907859	protein_coding	TBC1 domain family member 9B	54.22	TBC1D9B
ENSG00000197256	5133.310452	6212.467743	-1.67764563	0.98017341	-0.275276941	33524	52.98	19	11164267	11197791	protein_coding	KN motif and ankyrin repeat domains 2	52.98	KANK2
ENSG00000197324	1230.260776	1757.522102	-1.747012396	0.978787288	-0.514578679	9967	55.77	14	22871613	22881580	protein_coding	LDL receptor related protein 10	55.77	LRP10
ENSG00000197530	129.6237473	402.7853877	-2.14181324	0.98951662	-1.635681299	15195	66.41	1	1615415	1630610	protein_coding	mindbomb E3 ubiquitin protein ligase 2	66.41	MIB2
ENSG00000197562	71.73005536	154.4228877	-1.276986507	0.953111711	-1.106236948	39915	56.81	16	589357	629272	protein_coding	RAB40C, member RAS oncogene family	56.81	RAB40C
ENSG00000197616	8126.633626	13667.67011	-1.790303616	0.981456038	-0.750037572	26287	53.35	14	23381990	23408277	protein_coding	myosin heavy chain 6	53.35	MYH6
ENSG00000197785	67.07391355	144.8329757	-1.331600639	0.959768189	-1.110566427	22536	59.87	1	1512151	1534687	protein_coding	ATPase family, AAA domain containing 3A	59.87	ATAD3A
ENSG00000197858	505.112843	1040.475989	-2.394059153	0.987513662	-1.042566044	3626	64.3	8	144082590	144086216	protein_coding	glycosylphosphatidylinositol anchor attachment 1	64.3	GPAA1
ENSG00000197879	3107.516315	4140.532694	-1.79545795	0.982145699	-0.414054422	28714	60.07	17	1464098	1492812	protein_coding	myosin IC	60.07	MYO1C

ENSG00000197912	1608.930584	2094.476595	-1.417165571	0.969303526	-0.38048768	66851	52.29	16	89490917	89557768	protein_coding	SPG7, paraplegin matrix AAA peptidase subunit	52.29	SPG7
ENSG00000198276	432.3861284	751.3034458	-1.41861829	0.969526492	-0.797075479	16586	62.54	20	63939829	63956415	protein_coding	uridine-cytidine kinase 1 like 1	62.54	UCKL1
ENSG00000198324	266.8755962	443.2291709	-1.270881013	0.952491387	-0.731885447	8470	57.94	12	111360651	111369121	protein_coding	family with sequence similarity 109 member A	57.94	FAM109A
ENSG00000198355	385.8405181	678.4584457	-1.426188011	0.970719796	-0.814255804	3567	69.51	22	49960513	49964080	protein_coding	Pim-3 proto-oncogene, serine/threonine kinase	69.51	PIM3
ENSG00000198467	10925.6818	13830.99745	-1.728317373	0.978833647	-0.340181892	9028	56.38	9	35681992	35691020	protein_coding	tropomyosin 2	56.38	TPM2
ENSG00000198502	186.2788612	505.2099174	-1.221656103	0.950021626	-1.439418992	12944	42.84	6	32517343	32530287	protein_coding	major histocompatibility complex, class II, DR beta 5	42.84	HLA-DRB5
ENSG00000198517	340.7123437	688.7601195	-1.463083358	0.975223895	-1.015447395	12329	63.41	7	1530714	1543043	protein_coding	MAF bZIP transcription factor K	63.41	MAFK
ENSG00000198523	119102.6638	89015.25001	2.20606354	0.971204865	0.420081256	12432	34.41	6	118548298	118560730	protein_coding	phospholamban	34.41	PLN
ENSG00000198719	84.09442283	173.006518	-1.244124907	0.950806898	-1.040744364	24359	53.53	6	170282206	170306565	protein_coding	delta like canonical Notch ligand 1	53.53	DLL1
ENSG00000198816	192.3975777	403.5367188	-1.652179448	0.98144793	-1.068609319	5734	60.98	19	7515292	7521026	protein_coding	zinc finger protein 358	60.98	ZNF358
ENSG00000198837	802.8081554	1098.740972	-1.224372203	0.950178973	-0.452724134	17195	56.48	1	153929501	153946696	protein_coding	DENN domain containing 4B	56.48	DENND4B
ENSG00000198898	6688.542378	4742.882376	2.46500721	0.991351806	0.495927751	110979	38.99	7	116811070	116922049	protein_coding	capping actin protein of muscle Z-line alpha subunit 2	38.99	CAPZA2
ENSG00000198911	2579.624368	3220.324507	-1.29585795	0.955192906	-0.320045071	74229	48.21	22	41833079	41907308	protein_coding	sterol regulatory element binding transcription factor 2	48.21	SREBF2
ENSG00000198925	1480.649489	2034.20478	-1.654560005	0.981383578	-0.458234766	19945	55.14	2	219209772	219229717	protein_coding	autophagy related 9A	55.14	ATG9A
ENSG00000198931	215.7792717	388.2985209	-1.340285554	0.961060003	-0.847609929	2605	64.35	16	88809339	88811944	protein_coding	adenine phosphoribosyltransferase	64.35	APRT
ENSG00000203485	274.0178634	563.1444301	-1.845859472	0.985723409	-1.039235033	32929	63.24	14	104689606	104722535	protein_coding	inverted formin, FH2 and WH2 domain containing	63.24	INF2
ENSG00000203883	49.28881133	129.7443749	-1.384122812	0.965603977	-1.396339897	2059	70.68	20	64047582	64049641	protein_coding	SRY-box 18	70.68	SOX18
ENSG00000204301	321.4503055	756.3146655	-1.980718534	0.985440986	-1.234390872	29224	51.81	6	32194843	32224067	protein_coding	notch 4	51.81	NOTCH4
ENSG00000204370	5710.546817	4116.1508	2.758578715	0.999999934	0.47233306	33240	41.79	11	112086773	112120013	protein_coding	succinate dehydrogenase complex subunit D	41.79	SDHD
ENSG00000204463	3406.006811	4082.012127	-1.365800651	0.964179867	-0.261199148	13677	52.36	6	31639028	31652705	protein_coding	BCL2 associated athanogene 6	52.36	BAG6
ENSG00000204469	2175.83076	3039.527441	-1.875623246	0.986560285	-0.482280698	17051	53.44	6	31620720	31637771	protein_coding	proline rich coiled-coil 2A	53.44	PRRC2A
ENSG00000204525	3775.667699	4932.145439	-1.488786279	0.976065473	-0.385483546	3381	59.28	6	31268749	31272130	protein_coding	major histocompatibility complex, class I, C	59.28	HLA-C
ENSG00000204592	5589.267715	7310.249677	-1.724809646	0.978839552	-0.387261402	4738	55.48	6	30489467	30494205	protein_coding	major histocompatibility complex, class I, E	55.48	HLA-E
ENSG00000205356	193.0263562	355.1605721	-1.423620914	0.970311812	-0.879673582	37627	56.22	7	98214624	98252251	protein_coding	tectonin beta-propeller repeat containing 1	56.22	TECPR1
ENSG00000205363	646.3803237	1016.341025	-1.530850404	0.976210576	-0.652929381	17316	54.89	15	73735431	73752747	protein_coding	chromosome 15 open reading frame 59	54.89	C15orf59
ENSG00000206503	4060.247822	5535.171965	-1.538554893	0.976377743	-0.447060352	4624	55.18	6	29941260	29945884	protein_coding	major histocompatibility complex, class I, A	55.18	HLA-A
ENSG00000211450	59.09508387	148.891651	-1.405821619	0.967709018	-1.333152836	2304	51.67	11	57741250	57743554	protein_coding	selenoprotein H	51.67	SELENOH
ENSG00000212864	47.28488986	136.2000008	-1.636187461	0.981474808	-1.52627557	1334	68.39	9	137220247	137221581	protein_coding	ring finger protein 208	68.39	RNF208
ENSG00000213442	1747.42573	2520.815875	-1.435480021	0.97216624	-0.528659606	527	60.23	12	104265309	104265836	processed_pseudogene		60.23	RPL18AP3
ENSG00000213445	136.7895509	278.9331572	-1.467767404	0.975498938	-1.02796141	12833	60.73	11	65638097	65650930	protein_coding	signal-induced proliferation-associated 1	60.73	SIPA1
ENSG00000213465	273.3027225	463.0330865	-1.327395996	0.959150586	-0.760615455	8071	55.34	11	65014113	65022184	protein_coding	ADP ribosylation factor like GTPase 2	55.34	ARL2
ENSG00000213639	10022.54317	7123.228234	1.973250426	0.956453392	0.49264551	51300	38.98	2	28751640	28802940	protein_coding	protein phosphatase 1 catalytic subunit beta	38.98	PPP1CB
ENSG00000213923	1031.54622	1411.984812	-1.344097316	0.961612952	-0.452916105	27393	55.79	22	38290691	38318084	protein_coding	casein kinase 1 epsilon	55.79	CSNK1E
ENSG00000214063	247.3725947	487.1981098	-1.472880634	0.975725404	-0.977822857	24308	65.01	11	842808	867116	protein_coding	tetraspanin 4	65.01	TSPAN4
ENSG00000214357	890.9043999	1400.147012	-1.388548433	0.965948031	-0.652235781	50274	50.74	5	172641266	172691540	protein_coding	neuralized E3 ubiquitin protein ligase 1B	50.74	NEURL1B
ENSG00000215908	53.90400706	112.1239941	-1.283140765	0.953777115	-1.056630614	38979	52.04	1	16618253	16657232	transcribed_un|	ciliary rootlet coiled-coil, rootletin pseudogene 2	52.04	CROCCP2
ENSG00000224051	432.0987503	927.765442	-2.195336526	0.98572543	-1.10239905	4141	65.26	1	1324756	1328897	protein_coding	ceramide-1-phosphate transfer protein	65.26	CPTP
ENSG00000225663	433.653274	892.8052321	-1.919150614	0.988845795	-1.04180348	10941	59.8	17	81822361	81833302	protein_coding	MAPK regulated corepressor interacting protein 1	59.8	MCRIP1
ENSG00000227097	1300.211078	2596.097567	-2.419427972	0.984040985	-0.997598753	209	61.9	11	82689559	82689768	processed_pseudogene		61.9	RPS28P7
ENSG00000228300	28.30370873	79.02562622	-1.444819238	0.973462854	-1.481329456	3811	63.69	19	1275438	1279249	protein_coding	chromosome 19 open reading frame 24	63.69	C19orf24
ENSG00000230715	8.042153728	31.55328456	-1.504939641	0.976099924	-1.972136372	402	65.26	7	128652841	128653243	processed_pseudogene		65.26	
ENSG00000231686	12.73979448	35.48242611	-1.423829535	0.970344915	-1.477762654	987	63.97	X	146619597	146620584	processed_pseudogene		63.97	
ENSG00000231925	837.4738376	1208.866405	-1.366044083	0.964201977	-0.529538791	14693	50.01	6	33299694	33314387	protein_coding	TAP binding protein	50.01	TAPBP
ENSG00000233276	393.747533	726.6976821	-1.561890634	0.9774385	-0.88408442	1424	63.79	3	49357176	49358600	protein_coding	glutathione peroxidase 1	63.79	GPX1
ENSG00000234745	5119.095428	6897.831616	-1.24909055	0.950977905	-0.430254011	87697	42.89	6	31269491	31357188	protein_coding	major histocompatibility complex, class I, B	42.89	HLA-B
ENSG00000242802	67.82462656	155.4169209	-1.376740323	0.965061456	-1.19626248	18690	58.29	7	4775622	4794312	protein_coding	adaptor related protein complex 5 zeta 1 subunit	58.29	AP5Z1
ENSG00000243679	8.179954698	33.77667643	-1.684318266	0.979773759	-2.045862618	753	67.24	7	128653969	128654722	processed_pseudogene		67.24	
ENSG00000247596	536.2256811	1027.038116	-1.885616596	0.987280729	-0.937577504	10650	59.9	3	52228610	52239260	protein_coding	twinfilin actin binding protein 2	59.9	TWF2
ENSG00000249780	4.692886366	35.8578012	-1.34302993	0.961460034	-2.933739593	339	47.35	4	12640298	12640637	processed_pseudogene		47.35	
ENSG00000250479	2299.293667	3120.713612	-1.334363922	0.960179647	-0.440685227	2609	64.1	22	23765834	23768443	protein_coding	coiled-coil-helix-coiled-coil-helix domain containing 10	64.1	CHCHD10
ENSG00000251322	291.439307	613.6516338	-1.429083456	0.971178742	-1.074224409	58883	57.13	22	50674415	50733298	protein_coding	SH3 and multiple ankyrin repeat domains 3	57.13	SHANK3
ENSG00000255302	8029.473759	6204.741812	2.551972309	0.999995963	0.37193426	2297	41.64	15	48877886	48880183	protein_coding	EP300 interacting inhibitor of differentiation 1	41.64	EID1
ENSG00000261236	143.9797784	270.8684031	-1.372649191	0.964752554	-0.91172591	29268	62.27	8	144262102	144291370	protein_coding	block of proliferation 1	62.27	BOP1
ENSG00000262814	11.54166451	47.8516697	-1.597357685	0.97974177	-2.051717965	4169	57.67	17	81703357	81707526	protein_coding	mitochondrial ribosomal protein L12	57.67	MRPL12
ENSG00000272333	211.8916727	366.3398435	-1.27005617	0.952412513	-0.789855727	20859	59.77	19	35718019	35738878	protein_coding	lysine methyltransferase 2B	59.77	KMT2B
ENSG00000275023	1544.676471	2024.367598	-1.423165173	0.970239508	-0.390166587	24261	56.24	17	38705542	38729803	protein_coding	MLLT6, PHD finger containing	56.24	MLLT6

Supplementary Table S2. Predicted GO terms for biological process and molecular function of the genes

GO Biological Process

Term	Overlap	P-value	Adjusted P-value	Z-score	Combined Scor	Genes
cardiac muscle contraction (GO:0060048)	13/57	7.14712690213e-08	0.000453842558285	-2.683750501	44.1583510356	MYBPC3;KCNH2;GPX1;ACE;GAA;GRK2;TNNT2;KCNQ1;TCAP;ALDOA;OBSL1;MYH6;MYH7
cardiac muscle cell contraction (GO:0086003)	9/36	3.31103442812e-06	0.0080348186545	-2.36987178811	29.9036344566	MYBPC3;KCNH2;GRK2;GAA;TNNT2;KCNQ1;TCAP;MYH6;MYH7
basement membrane organization (GO:0071711)	17/144	1.3918583496e-05	0.0080348186545	-3.1224851307	34.9165207274	COL18A1;LAMA5;SPARC;VWF;LAMB2;FURIN;HSPG2;COL1A1;COL3A1;COL4A2;ADAM15;COL4A1;COL6A2;BSG;COL6A1;PXDN;AGRN
biofilm matrix organization (GO:0098784)	17/143	1.26853018082e-05	0.0080348186545	-3.10669347986	35.0281758063	COL18A1;LAMA5;SPARC;VWF;LAMB2;FURIN;HSPG2;COL1A1;COL3A1;COL4A2;ADAM15;COL4A1;COL6A2;BSG;COL6A1;PXDN;AGRN
extracellular matrix organization in marginal zone involved in cerebral cortex radial glia guided migration (GO:0021820)	17/143	1.26853018082e-05	0.0080348186545	-3.09977717801	34.9501940425	COL18A1;LAMA5;SPARC;VWF;LAMB2;FURIN;HSPG2;COL1A1;COL3A1;COL4A2;ADAM15;COL4A1;COL6A2;BSG;COL6A1;PXDN;AGRN
cellulose microfibril organization (GO:0010215)	17/143	1.26853018082e-05	0.0080348186545	-3.10551910715	35.0149346758	COL18A1;LAMA5;SPARC;VWF;LAMB2;FURIN;HSPG2;COL1A1;COL3A1;COL4A2;ADAM15;COL4A1;COL6A2;BSG;COL6A1;PXDN;AGRN
extracellular matrix organization involved in endocardium development (GO:0061148)	17/143	1.26853018082e-05	0.0080348186545	-3.09906633408	34.9421792299	COL18A1;LAMA5;SPARC;VWF;LAMB2;FURIN;HSPG2;COL1A1;COL3A1;COL4A2;ADAM15;COL4A1;COL6A2;BSG;COL6A1;PXDN;AGRN
extracellular matrix organization (GO:0030198)	17/143	1.26853018082e-05	0.0080348186545	-3.10527723319	35.0122075308	COL18A1;LAMA5;SPARC;VWF;LAMB2;FURIN;HSPG2;COL1A1;COL3A1;COL4A2;ADAM15;COL4A1;COL6A2;BSG;COL6A1;PXDN;AGRN
endocytosis involved in viral entry into host cell (GO:0075509)	13/80	4.35097093759e-06	0.0080348186545	-2.94198959106	36.3191896327	CD81;INPPL1;TSC2;AP2A1;CSNK1E;DNM2;EHD1;GRK2;HOOK2;HYAL2;TOM1;GAS6;CSNK1G2
extracellular matrix assembly (GO:0085029)	18/149	5.65818460904e-06	0.0080348186545	-3.15211886175	38.0851844426	COL18A1;LAMA5;SPARC;VWF;LAMB2;FURIN;HSPG2;COL1A1;COL3A1;COL4A2;ADAM15;COL4A1;COL6A2;BSG;COL6A1;PXDN;AGRN;GAS6

GO Molecular Function

Term	Overlap	P-value	Adjusted P-value	Z-score	Combined Scor	Genes
cadherin binding involved in cell-cell adhesion (GO:0098641)	36/294	9.19632470815e-11	7.16393694765e-08	-2.87398300992	66.416690041	NOTCH3;TWF2;CORO1B;FAM129B;SLC9A3R2;PUF60;PPP1R13L;PCBP1;BSG;EFHD2;FLNA;RPS2;SH3GL1;SCYL1;LYPLA2;JUP;DAB2IP;SCRIB;EEF2;HCFC1;RAB11B;EHD1;CTTN;GIPC1;FSCN1;PLIN3;CDC42EP1;MYH9;RPL29;TLN1;ALDOA;CC2D1A;DBN1;PFKP;EIF4G1;PLEC
cadherin binding (GO:0045296)	35/275	5.71569092837e-11	7.16393694765e-08	-2.81329709452	66.3522332504	NOTCH3;TWF2;CORO1B;FAM129B;SLC9A3R2;PUF60;PPP1R13L;PCBP1;BSG;EFHD2;FLNA;RPS2;SH3GL1;SCYL1;LYPLA2;JUP;DAB2IP;SCRIB;EEF2;HCFC1;RAB11B;EHD1;CTTN;GIPC1;FSCN1;PLIN3;MYH9;RPL29;TLN1;ALDOA;CC2D1A;DBN1;PFKP;EIF4G1;PLEC
protein kinase binding (GO:0019901)	32/352	1.15548013616e-06	0.000600079350711	-2.98492621931	40.8069102199	HSPB1;AP2A1;PTPN23;PKD1;RPTOR;HSF1;DVL1;FLNA;MAP2K7;SLC12A7;TNS2;SLC12A4;FBXW5;BAD;DAB2IP;RAD23A;EEF2;TOM1L2;PER1;PFKL;TOLLIP;DGKQ;CDC37;TRIP6;EEF1A2;DOK7;STUB1;SQSTM1;LLGL1;MYH6;BCAR1;TELO2
actin filament binding (GO:0051015)	22/208	4.97214701632e-06	0.00193665126286	-2.69725355245	32.9379401225	MYBPC3;FLII;TPM2;HIP1R;ACTN4;SPTB;TRIOBP;CORO1B;SMTN;MAEA;TNNT2;GIPC1;CORO6;MYO18A;FSCN1;MYH14;FLNA;MYH9;SYNPO;ALDOA;OBSL1;DBN1
kinase binding (GO:0019900)	11/62	9.92231502989e-06	0.00309179336331	-2.19229645459	25.2568430239	PER1;PFKL;TOLLIP;CDC37;DGKQ;TRIP6;DAB2IP;FLNA;RAD23A;STUB1;TNS2
phosphatidylinositol 3-kinase catalytic subunit binding (GO:0036313)	11/64	1.36129883527e-05	0.00353483930891	-2.14992550041	24.0888105897	PER1;PFKL;TOLLIP;CDC37;DGKQ;TRIP6;DAB2IP;FLNA;RAD23A;STUB1;TNS2
actin lateral binding (GO:0003786)	14/109	3.05884345426e-05	0.00680811157391	-2.26594714495	23.5542680921	MYBPC3;TPM2;HIP1R;ACTN4;SPTB;CORO1B;TRIOBP;CORO6;FSCN1;MYO18A;MYH14;FLNA;MYH9;OBSL1
mitogen-activated protein kinase kinase binding (GO:0031434)	26/305	3.50994989436e-05	0.00683562741927	-2.89593590844	29.7045520352	ARAF;HSPB1;AP2A1;PTPN23;PKD1;RPTOR;HSF1;DVL1;MAP2K7;SLC12A7;SLC12A4;ACE;FBXW5;BAD;DAB2IP;EEF2;TOM1L2;TAOK2;EEF1A2;DOK7;PIN1;SQSTM1;LLGL1;MYH6;BCAR1;TELO2
integrin binding involved in cell-matrix adhesion (GO:0098640)	11/78	9.09122929083e-05	0.015737928039	-1.9649903343	18.285444179	LAMA5;COL3A1;TNXB;CD151;VWF;ADAM15;LTBP4;TSPAN4;EMILIN1;ACTN4;TLN1
eukaryotic translation initiation factor 2alpha kinase activity (GO:0004694)"	22/296	0.000906085723927	0.0273959114111	-2.93048588509	20.5320878444	CAMK2B;CDK18;SRPK3;MAP2K2;DMPK;ARAF;CSNK1E;BCKDK;STK11;AKT2;WNK2;FASTK;TAOK2;MKNK2;AKT1;MAP3K10;ULK1;PIM3;PKN1;SQSTM1;MAP3K11;CSNK1G2

Supplementary Table S3. Predicted upstream regulators identified in HFpEF proxy using Ingenuity Pathway Analysis. (Activation Z-score ≥±2, p-value of overlap≤0.05).

Upstream Regulat	Molecule Type	Predicted Ac	Activati	p-value of	Target molecules in DE gene set
TP53	transcription regulator	Inhibited	-3,641	5,03E-09	A2M,ACE,ACOT11,ACTN4,ADAMTSL4,AKT1,APOE,ATG4B,CAMK2B,CARD10,CD81,CKB,CKM,CLIC4,CLPP,COL18A1,COL1A1,COL3A1,COL4A1,COL4A2,COL6A2,COMT,COQ8A,CTDSP1,CTSD,DLD,DNM2,DPYSL4,ECH1,EMILIN1,ERCC1,GADD45G,GAS6,GPI,GPX1,GSTP1,H19,HDAC5,HLA-B,HSF1,HSPB1,HSPB7,HSPG2,IDH2,IGF2,IGFBP2,IGFBP3,IGFBP5,IRF7,ISG15,KANK3,KLHL21,KRT8,LAMA5,LAMB2,LSS,MAP2K2,MAP2K7,MTA1,MYH9,MYL9,MYO1C,NCOR2,NDUFA4L2,NINJ1,NLRX1,NOTCH1,PFKP,PLXNB2,POLD1,POLR2E,PPM1F,PPP1R13L,PRDX3,PTGDS,PTP4A3,RAD23A,RALY,RUVBL2,SCRIB,SEPT5,SHISA5,SIVA1,SPHK2,STK11,TINAGL1,TOM1,TP53I11,TRIM28,TSC2,TSPO,ULK1,UNC5B,VEGFB,YPEL3,ZYX
ERBB2	kinase	Inhibited	-2,783	9,59E-07	AKT1,AKT2,ALDOC,BCAT2,CAPN15,CAPZA2,CDC37,CHCHD10,COL18A1,COL1A1,COL3A1,COL4A1,COL6A1,COL6A2,DGAT1,DLL1,FSCN1,GAS6,GPAA1,GPC1,H19,HSPB1,IGF2,IGFBP3,IGFBP5,JAG2,JUP,KIFC3,LGALS1,LTBP3,LUM,MAPK8IP3,MKNK2,MYL9,NDUFV1,NOTCH1,NOTCH3,NOTCH4,NR1H2,PHLDB1,PIN1,POLD1,POLR2E,POLR2L,PQLC1,SKP1,SMTN,SPARC,TAPBP,TK1,VWF,WFS1
MKNK1	kinase	Inhibited	-3,742	7,85E-06	AGRN,AP3D1,ARVCF,ATP1A3,FLNA,GPS1,ISG15,LAMB2,MAPK8IP3,NCDN,PLXNB2,RNH1,SPARC,STRN4
FAS	transmembrane receptor	Activated	2,091	1,17E-05	AKT2,ARFGAP1,ARHGEF1,BAD,CABIN1,CAMK2B,CLTB,COL18A1,COL1A1,COL3A1,COL4A1,COL4A2,COL6A1,CSNK1G2,DGCR2,DGKZ,ERCC1,FSCN1,GADD45G,GNAI2,GTPBP6,HLA-DQB1,LUM,NUMA1,PLEC,RGS3,SIPA1,SPG7,TWF2,TYK2
KDM5A	transcription regulator	Activated	2,84	3,54E-05	DMPK,GADD45GIP1,MAP2K7,MRPL12,MRPL55,MYH6,MYH7,NDUFA3,NDUFA5,NDUFS7,NDUFV1,SDHD,TCAP,TNNT2,UQCRC1
SRF	transcription regulator	Inhibited	-2,58	3,55E-05	A2M,CENPB,CKM,CLDN5,FLNA,G0S2,GADD45G,GATA4,HSPG2,LDB3,LRP1,MCF2L,MYH6,MYH7,MYH9,MYL9,NKX2-5,RAMP1,RNH1,SCYL1,SMTN,SREBF2,TCAP,TLN1,TNNT2,TST,ZYX
MAP4K4	kinase	Activated	2,673	4,70E-05	ACACB,ACADS,ARAF,BCAT2,CYC1,DLD,HYAL2,MGLL,NDUFA3,NDUFA5,NDUFB7,NDUFS8,NDUFV1,UQCRC1
ESR1	ligand-dependent nuclear receptor	Activated	3,578	8,04E-05	ACE,AKT1,AKT2,ANAPC2,AP1M1,APOE,ARAF,ARFGAP1,CAMK2B,CKB,COMT,COPE,CTSD,DNM2,ESRRA,FLNA,H19,HIP1R,HLA-B,HLA-C,HLA-E,HOOK2,IFI27,IFITM3,IGF2,IGFBP2,IGFBP3,IGFBP5,KDM4B,LAMB2,LGALS1,LGALS3BP,LTBP4,MAP2K2,MAP2K7,MAPK8IP3,MCF2L,MIB2,NOTCH1,NOTCH3,NR1H2,NUCB1,OBSCN,PHLDB1,PPP2R1A,PPP6R2,RGS3,RHOQ,SGSM3,SHANK3,SHC2,SIGIRR,SLC12A4,SLC2A4RG,SLC6A8,SPG7,SQSTM1,SYNPO,TAPBP,TIMM13,TOM1,TP53I11,TSC2,TSPO,TYK2,UBALD1,USP19,VPS28,YPEL3,ZBTB7B
HTT	transcription regulator	Inhibited	-3,286	1,65E-04	ADCY5,AGRN,AKT1,AKT2,ALDOA,ALDOC,APOE,ATP1A3,CAMK2B,CKM,COL18A1,COL4A1,COL4A2,COL6A1,COX6A2,CSNK1E,CTDSP1,CTSD,CTTN,CYC1,CYCS,DLL1,ECI1,EEF2,FAM173A,GADD45G,GPC1,GPI,GRK2,IGF2,IGFBP5,JUP,NDUFA3,NDUFA5,NDUFS7,NFIC,PER1,PITPNM1,PTPN23,SGTA,SQSTM1,STRN4,TCF3
IGF1R	transmembrane receptor	Inhibited	-2,246	3,10E-04	ACADS,ACTN4,ALDOA,ATP5D,COL18A1,COL1A1,COL3A1,COL4A1,COL4A2,COX6A2,CTSD,CYCS,FLNC,FTH1,H19,IGF2,IGFBP3,IGFBP5,MYH7,NDUFA3,UQCRC1
VEGFA	growth factor	Inhibited	-3,098	3,92E-04	ACE,ADAM15,AKT1,BCAT2,CDH5,CLIC4,COL1A1,DLL1,EPHB4,HLA-DQB1,IGFBP5,LMNA,MMP15,MRPS12,NOTCH1,NOTCH4,SDHD,SMTN,SPARC,TIMM13,TSPO,UNC5B
miR-296-5p	mature microrna	Activated	2,186	4,48E-04	AKT1,COL1A1,HGS,LYPLA2,SCRIB
PLAG1	transcription regulator	Inhibited	-2,767	5,00E-04	CLTB,CRIP2,FLNC,IGF2,PLEC,PTP4A3,SRPK3,TSPAN4
EBF1	transcription regulator	Inhibited	-2,646	5,19E-04	ACACB,AKT2,ARAF,COL1A1,INPPL1,IRF7,MKNK2,PFKP,PPP1CB,PYGB,RHOQ,RPTOR,TCF3
MYOD1	transcription regulator	Inhibited	-2,29	7,40E-04	ACACB,ALDOA,CKM,COL4A1,GADD45G,IGF2,IGFBP5,KIFC3,LDB3,MYH7,RAPSN,RBM38,RRAS,SGCA,TNNT2,TNS1
RICTOR	other	Activated	2,441	7,54E-04	ATP5D,BAD,BSG,COX6A2,CYC1,ISG15,NDUFA3,NDUFA4L2,NDUFA5,NDUFB7,NDUFS6,NDUFS7,NDUFS8,NDUFV1,RPL28,RPLP2,RPS15,RPS2,SDHD,UQCRC1
UHRF2	enzyme	Inhibited	-2,236	1,10E-03	AKT1,CKB,CRIP2,JAG2,SIVA1
HIF1A	transcription regulator	Inhibited	-2,904	1,40E-03	ADAM15,AKT1,ALDOA,ALDOC,APOE,ATG9A,CABIN1,CD164,CTSD,EIF4G1,FSCN1,FURIN,GPI,HSPB1,IGF2,IGFBP2,IGFBP3,IGFBP5,JUP,KDM4B,KIFC2,LGALS1,NOTCH1,PFKL,POLRMT,SLC29A1
STAT4	transcription regulator	Inhibited	-4,088	1,72E-03	ALDOC,BAD,BSG,FSCN1,FURIN,GADD45G,HLA-DQB1,ISG15,JAG2,LRP1,LTBP3,MAPK8IP3,PER1,PPP1R13L,RAMP1,VSIR,ZNF524
FOS	transcription regulator	Inhibited	-2,66	1,96E-03	A2M,AP3D1,ARF1,CD164,COL18A1,COL1A1,COL6A1,CYCS,E4F1,ERCC1,FBLIM1,FTH1,GAK,GATA4,GSTP1,HLA-B,IGFBP5,KRT8,LGALS3BP,LTBP3,MAFK,MT2A,MYH6,MYH9,NDUFS7,NOTCH4,PKN1,POMC,PTGDS,RGMA,RNH1,RXRA
Smad2/3-Smad4	complex	Inhibited	-2	2,19E-03	COL1A1,IGFBP3,IGFBP5,IRF7
PRL	cytokine	Inhibited	-2,852	2,56E-03	A2M,BST2,CLDN5,COL1A1,COL3A1,CTSD,DVL1,GPSM1,HELZ2,IGF2,IGFBP3,IGFBP5,IRF7,ISG15,PIM3,RABAC1,RASD1,SHISA5,SPARC
B2M	transmembrane receptor	Inhibited	-2,236	2,67E-03	HLA-A,HLA-DQB1,HLA-DRB5,HLA-E,TAPBP
mir-122	microrna	Activated	2,259	2,96E-03	ALDOA,BCAT2,CLIC4,FBLIM1,GIT1,GPT,PLIN3,SEPT9,SH3GL1
WNT11	other	Inhibited	-2,232	3,11E-03	GATA4,MYH6,MYH7,NKX2-5,TNNT2
HOXA10	transcription regulator	Activated	2,138	3,22E-03	APOE,ARL2,CHERP,CLDN5,COL3A1,GAS6,HLA-DQB1,IDH2,IGFBP3,IGFBP5,PPP1R13L,SCOC,SLC27A1,TNXB
DLL4	other	Activated	2,236	3,61E-03	EGFL7,EPHB4,ESAM,LGALS1,UNC5B
HEY2	transcription regulator	Activated	2,193	3,61E-03	GATA4,MYH6,MYH7,NKX2-5,SMTN
TGFB1	growth factor	Inhibited	-2,906	4,32E-03	ABCD1,ACE,AKT1,APOE,ATG4B,BAD,BSG,CALM1 (includes others),CAND2,CAPN15,CCDC85B,CDH5,CKM,CLIC4,COL18A1,COL1A1,COL3A1,COL4A1,COL4A2,COL6A1,COL6A2,CRIP2,CTSD,CTTN,CYC1,EMILIN1,ERF,ESAM,FAM53B,FLNA,FSCN1,FTH1,FURIN,GNAI2,GPT,HLA-DQB1,HLA-DRB5,HSPB1,HSPG2,IFI27,IGF2,IGFBP2,IGFBP3,IGFBP5,INHA,ITPK1,JAG2,JUP,KRT8,LTBP3,MAP3K11,MAPK8IP3,MXD4,MYH7,MYH9,MYO1C,NOTCH1,NOTCH3,PCBP1,PITPNM1,POLD1,POMC,PTGDS,PTP4A3,RNH1,RXRA,SHISA5,SLC12A4,SLC12A7,SLC22A18,SLC4A2,SMTN,SPARC,TNNT2,TPM2,ULK1,VPS28,VWF,WFS1,ZYX
IPMK	kinase	Inhibited	-2	5,89E-03	FLNA,FLNC,MYL9,PER1
SYVN1	transporter	Inhibited	-3,317	6,66E-03	CALM1 (includes others),CD151,GNB2,HLA-A,HLA-C,HSPB1,LGALS3BP,MED25,MGRN1,PCBP1,PNPLA2
NFE2L2	transcription regulator	Inhibited	-4,358	7,89E-03	ALDOA,APBA3,ARF1,CDC34,CLPP,COL1A1,COL3A1,COL4A2,CTSD,EPHB4,FTH1,GADD45G,GNA11,GNAI2,GPX1,GSTP1,KEAP1,KIFC2,LMNA,MGLL,PCBP1,PIP5K1C,PKIA,SQSTM1

IL4	cytokine	Inhibited	-3,806	8,59E-03	ABCA2,ALDOC,APOE,APRT,ATP1A3,BCAT2,BOP1,CENPB,CLDN5,CLUH,COL18A1,COL1A1,COL6A2,EPHB4,FLNA,GADD45G,GCAT,GPT,IRF7,ISG15,LGALS1,LGALS3BP,LRP1,LYL1,MYH9,NUMA1,PFKL,PFKP,PIN1,PLEC,PRMT1,SLC29A1,SPHK2,SQSTM1,STAB1,STUB1,SYNGR2,TAPBP,TINAGL1,TLN1,TM7SF2,TNFRSF4,UQCRC1
miR-199a-5p	mature microrna	Activated	2,646	9,44E-03	BCAM,COL1A1,COL4A1,IFI27,ISG15,MYL9,SMTN
GLI1	transcription regulator	Inhibited	-2,745	9,58E-03	AKT1,CDC34,COL1A1,GATA4,IGF2,JAG2,JUP,LMNA,NPDC1,POMC,RXRA,SOX18,TOM1,ULK1
ACOX1	enzyme	Activated	2,714	1,01E-02	COL4A2,CRAT,FABP2,GSTP1,HLA-DRB5,IGFBP2,KRT8,LGALS3BP,LONP1,MGLL,SQSTM1
miR-124-3p	mature microrna	Activated	3,219	1,13E-02	ARAF,ARHGEF1,CD164,CTDSP1,DNM2,FAM129B,GAS2L1,HDAC5,MYH9,NFIC,PPP1R13L,RNPEPL1,SYNGR2,TLN1,TSC22D4
miR-125b-5p	mature microrna	Activated	2,183	1,37E-02	ANAPC16,CDH5,DUS1L,IGFBP3,MAP2K7,MAP3K11,RABL6,VSIR
DNMT3B	enzyme	Activated	2,53	1,51E-02	ABCD1,ADCY5,CKM,GATA4,HLA-DQB1,IFI27,MYH14,MYH6,MYH7,MYH7B,MYL9,TNNT2
SMARCA4	transcription regulator	Inhibited	-4,239	1,60E-02	A2M,ACE,ACTN4,ADAM15,ATG9A,CCDC85B,CKM,COL1A1,CORO6,CSNK1G2,FBLIM1,GADD45G,GCAT,GPX1,GSTP1,HLA-B,HLA-C,HLA-E,IFI27,IFITM3,IGFBP5,KIFC3,LGALS1,LMNA,LUM,MXD4,PCDH1,PHLDB1,PKIA,RAMP1,TNNT2
IKBKG	kinase	Inhibited	-2,022	1,60E-02	GPT,GRN,HLA-A,IRF7,ISG15,KCNH2,MYH6,MYH7,PLD3,RABEP2
INSR	kinase	Inhibited	-2,785	1,63E-02	ACADS,ACTN4,ATP5D,COX6A2,CRAT,CYC1,CYCS,ECI1,FLNC,FURIN,GRN,H19,IGF2,LGALS1,LRP10,LSS,MYH7,NDUFA3,RUVBL2,SLC29A1,SREBF2,UQCRC1
TRIM24	transcription regulator	Activated	2,646	1,73E-02	AGRN,IRF7,ISG15,LGALS3BP,NUMA1,PACSIN3,PLEC,SHISA5
TP73	transcription regulator	Inhibited	-2,48	2,06E-02	COL18A1,COL1A1,CTSD,IDH2,IGFBP3,IGFBP5,JAG2,KCNQ1,KLHL21,LDHD,PTP4A3,ROBO4,SHISA5,SLC4A3,SPHK2,STK11,TP53I11,TSC2,ULK1
RB1	transcription regulator	Inhibited	-3,178	2,33E-02	AKT1,CKM,DMPK,GADD45GIP1,KANK2,MAP2K7,MRPL12,MRPL55,MYH6,MYH7,NDUFA3,NDUFA5,NDUFS7,NDUFV1,NKX2-5,POMC,SDHD,TCAP,TNNT2,ULK1,UQCRC1
IgG	complex	Activated	2,714	2,73E-02	APOE,CLDN5,COL3A1,GPX1,GUK1,HLA-E,HSPB1,IFITM3,ISG15,LGALS1,TAPBP
SCAP	other	Inhibited	-2,219	3,33E-02	ACACB,ALDOC,LSS,SREBF2,TM7SF2
IFI16	transcription regulator	Inhibited	-2,425	4,17E-02	BAD,CD81,GATA4,GPX1,ISG15,NKX2-5
IFNL1	cytokine	Inhibited	-2,449	4,17E-02	BST2,HLA-C,IFI27,IFITM3,ISG15,LGALS3BP
EGLN	group	Activated	2,646	4,46E-02	ALDOC,BCKDK,DPYSL4,HLA-B,PFKP,RTL8C,SLC27A1
RUVBL1	transcription regulator	Inhibited	-2	4,57E-02	ADAM15,IGFBP3,KDM4B,KIFC2
Growth hormone	group	Inhibited	-2,185	4,91E-02	APOE,COL18A1,COL1A1,COL3A1,COMT,FTH1,GPT,GSTP1,IGF2,IGFBP2,IGFBP3,IGFBP5,PRDX3

Column descriptions: ID; Regulator Effects network ID, Consistency Score; The Consistency Score is a measure of how causally consistent and densely connected a Regulator Effects network is, Node total; Sum of the number of regulators, targets and disease/Function nodes, Regulator total; Number of regulators in the network, Regulators; List of regulators participating in the network, Target total; Number of Targets from your dataset interacting with the regulators, Targets; List of the targets in the network, Diseases and Functions total; Number of Functions and Diseases in the network, Diseases and Functions; List of the Diseases and Functions, Known Regulator-Disease-Function Relationship; Percent of possible regulator to disease/function relationships that are already
known. For example, in a particular Regulator Effects network that consists of 4 regulators and 2 disease and functions nodes there are 8 possible relationships. If none of the relationships were known intheIngenuityKnowledgeBasethentheresultswouldbe“0%(0/8)”. Networkswitha0%valueare ones where there is no know prior relationship between the regulators and the diseases/functions.

Supplementary Table S4. Regulatory effect networks identified using Ingenuity Pathway Analysis.

ID	Consistency Score	Node Total	Regulator Total	Regulators	Target Total	Target Molecules in DE gene set	Disease & Function Total	Diseases & Functions (A; predicted activation, I; predicted inhibition)	Known Regulator-Disease/Function Relationship
**1**	9.500	26	5	FOS,HEY2, IFI16,SRF, WNT11	16	CKM,FLNA,GATA4,GPX1,HSPG2,LDB3,MYH6,MYH7,MYH9,NKX2-5,PKN1,RXRA,SMTN,TCAP,TNNT2,ZYX	5	Congenital heart disease (A), Failure of heart (A), Familial heart disease (A), Heart rate (I), Necrosis of cardiac muscle (A)	16% (4/25)
**2**	7.216	23	6	DNMT3B,HEY2,IFI16, RB1,SRF,WNT11	14	ADCY5,DMPK,FLNA,GATA4,GPX1,HSPG2,LDB3,MYH6,MYH7,MYH9, NKX2-5,SMTN,TCAP,TNNT2	3	Congenital heart disease (A), Familial heart disease (A), Heart rate (I)	22% (4/18)
**3**	6.500	25	4	HEY2,IFI16,SMARCA4,WNT11	16	ACE,ACTN4,ADAM15,CD81,COL1A1,GATA4,GPX1,LGALS1,LMNA,LUM,MYH6,MYH7,NKX2-5,RAMP1,SMTN,TNNT2	5	Congenital heart disease (A), Contraction of heart (I), Development of vasculature (I), Fibrosis of heart (A), Necrosis of cardiac muscle (A)	45% (9/20)
**4**	5.196	19	5	FOS,HEY2, SRF,TGFB1,WNT11	12	AKT1,FLNA,GATA4,GNAI2,HSPB1, HSPG2,MYH6,NKX2-5,NOTCH1,PKN1,RXRA,ZYX	2	Conotruncal heart malformations (A), Necrosis of cardiac muscle (A)	10% (1/10)
**5**	2.121	11	2	DLL4,Growth hormone	8	APOE,COL18A1,EGFL7,EPHB4,ESAM,IGF2,IGFBP3,LGALS1	1	Migration of endothelial cells (I)	50% (1/2)
**6**	2.121	12	1	GLI1	8	AKT1,COL1A1,GATA4,IGF2,JUP,LMNA,RXRA,SOX18	3	Cardiac contractility (I), Function of cardiac muscle (I), Vasculogenesis (I)	33% (1/3)
**7**	0.756	11	2	HIF1A,HTT	7	ADCY5,APOE,CKM,GRK2,IGF2,JUP,NOTCH1	2	Cardiac contractility (I), Hypoplasia of heart ventricle (A)	0% (0/4)
**8**	-4.536	9	1	GLI1	7	AKT1,COL1A1,GATA4,IGF2,JUP,RXRA,SOX18	1	Angiogenesis (I)	100% (1/1)
**9**	-5.367	7	1	TP53	5	APOE,COL3A1,GPX1,HSPG2,SPHK2	1	Damage of blood vessel (A)	0% (0/1)
**10**	-5.814	7	1	IgG	5	APOE,COL3A1,GPX1,HSPB1,LGALS1	1	Angiogenesis (I)	100% (1/1)
**11**	-6.000	6	1	TP73	4	COL18A1,IGFBP3,PTP4A3,STK11	1	Migration of endothelial cells (I)	0% (0/1)
**12**	-7.506	5	1	MYOD1	3	CKM,MYH7,TNNT2	1	Contractility of cardiac muscle (I)	0% (0/1)
**13**	-7.506	5	1	TGFB1	3	JUP,NOTCH1,RXRA	1	Hypoplasia of trabeculae carne (A)	0% (0/1)
**14**	-7.538	13	1	ERBB2	11	AKT1,COL18A1,COL4A1,GAS6,GPC1,HSPB1,IGF2,IGFBP3,LGALS1,SPARC,VWF	1	Cell movement of endothelial cells (I)	100% (1/1)
**15**	-12.522	7	1	VEGFA	5	ADAM15,AKT1,CDH5,EPHB4,SPARC	1	Migration of endothelial cells (I)	100% (1/1)
**16**	-19.230	7	1	IGF1R	5	COL18A1,COL4A1,COL4A2,IGF2,IGFBP3	1	Migration of endothelial cells (I)	0% (0/1)

Column descriptions: ID; Regulator Effects network ID, **Consistency Score;** The Consistency Score is a measure of how causally consistent and densely connected a Regulator Effects network is, **Node total;** Sum of the number of regulators, targets and disease/Function nodes, **Regulator total; Re** Number of regulators in the network**, Regulators;** List of regulators participating in the network, **Target total;** Number of Targets from your dataset interacting with the regulators, **Targets;** List of the targets in the network, **Diseases and Functions total;** Number of Functions and Diseases in the network, **Diseases and Functions;** List of the Diseases and Functions, **Known Regulator-Disease-Function Relationship;** Percent of possible regulator to disease/function relationships that are already known. For example, in a particular Regulator Effects network that consists of 4 regulators and 2 disease and functions nodes there are 8 possible relationships. If none of the relationships were known in the Ingenuity Knowledge Base then the results would be "0% (0/8)". Networks with a 0% value are ones where there is no know prior relationship between the regulators and the diseases/functions.
